# Mosquito Cell Atlas: A single-nucleus transcriptomic atlas of the adult *Aedes aegypti* mosquito

**DOI:** 10.1101/2025.02.25.639765

**Published:** 2025-02-25

**Authors:** Olivia V. Goldman, Alexandra E. DeFoe, Yanyan Qi, Yaoyu Jiao, Shih-Che Weng, Leah Houri-Zeevi, Priyanka Lakhiani, Takeshi Morita, Jacopo Razzauti, Adriana Rosas-Villegas, Yael N. Tsitohay, Madison M. Walker, Ben R. Hopkins, Mosquito Cell Atlas Consortium, Omar S. Akbari, Laura B. Duvall, Helen White-Cooper, Trevor R. Sorrells, Roshan Sharma, Hongjie Li, Leslie B. Vosshall, Nadav Shai

**Affiliations:** 1 Laboratory of Neurogenetics and Behavior, The Rockefeller University, New York, NY 10065, USA; 2 Kavli Neural Systems Institute, New York, NY 10065, USA; 3 Howard Hughes Medical Institute, New York, NY 10065, USA; 4 Huffington Center on Aging, Baylor College of Medicine, Houston, TX 77030, USA; 5 Department of Molecular and Human Genetics, Baylor College of Medicine, Houston, TX 77030, USA; 6 Department of Genetics, Yale School of Medicine, New Haven, CT 06510, USA; 7 School of Biological Sciences, Department of Cell and Developmental Biology, University of California, San Diego, La Jolla, CA 92093, USA; 8 Price Family Center for the Social Brain, The Rockefeller University, New York, NY 10065, USA; 9 Department of Evolution and Ecology, University of California Davis, Davis, CA 95616, USA; 10 Department of Biological Sciences, Columbia University, New York, NY 10027, USA; 11 School of Biosciences, Cardiff University, Museum Avenue, Cardiff, CF10 3AT, UK; 12 Wu Tsai Institute, Yale University, New Haven, CT 06510, USA; 13 Howard Hughes Medical Institute, New Haven, CT 06510, USA; 14 Program for Computational and Systems Biology, Sloan Kettering Institute, Memorial Sloan Kettering Cancer Center, New York, NY 10065, USA; 15 Single-cell Analytics Innovation Lab, Sloan Kettering Institute, Memorial Sloan Kettering Cancer Center, New York, NY 10065, USA

**Keywords:** *Aedes aegypti*, mosquito, snRNA-seq, cell atlas, sexual dimorphism

## Abstract

The female mosquito’s remarkable ability to hunt humans and transmit pathogens relies on her unique biology. Here, we present the Mosquito Cell Atlas (MCA), a comprehensive single-nucleus RNA sequencing dataset of more than 367,000 nuclei from 19 dissected tissues of adult female and male *Aedes aegypti*, providing cellular-level resolution of mosquito biology. We identify novel cell types and expand our understanding of sensory neuron organization of chemoreceptors to all sensory tissues. Our analysis uncovers male-specific cells and sexually dimorphic gene expression in the antenna and brain. In female mosquitoes, we find that glial cells in the brain, rather than neurons, undergo the most extensive transcriptional changes following blood feeding. Our findings provide insights into the cellular basis of mosquito behavior and sexual dimorphism. The MCA aims to serve as a resource for the vector biology community, enabling systematic investigation of cell-type specific expression across all mosquito tissues.

## Introduction

Mosquito-borne diseases affect hundreds of millions of people worldwide, with infection rates rising globally each year^1,2^. Furthermore, climate change-driven habitat expansion is predicted to put nearly 50% of the global population at risk for viral infection from *Aedes* mosquitoes by 2050^3,4^. *Aedes aegypti* is the primary vector for mosquito-borne viruses, including dengue, Zika, yellow fever, and chikungunya^5,6^. The most effective way to control mosquito-borne diseases remains the management of vector mosquito populations. However, despite advances in insecticidal and genetic control strategies^7^, adaptations of both mosquitoes and pathogens can render interventions less effective. There is a need for deeper insights into mosquito cellular and molecular biology to innovate methods of mitigating the spread of mosquito-borne disease.

The profound sexual dimorphism of *Aedes aegypti* mosquitoes is fundamental to the public health threat they pose to humans. Mosquitoes are attracted to human cues, including exhaled carbon dioxide (CO_2_), body heat, and skin odor^8–11^. Only females feed on blood, which provides the proteins and other nutrients that they require for reproduction. Humans are the preferred host for female *Aedes aegypti*, contributing to their effectiveness as a disease vector^12,13^. After consuming a blood meal, females undergo physiological and behavioral changes, including suppressed host seeking and generally reduced activity for 48–72 hours while they develop their eggs and find a suitable oviposition site guided by sensory attraction to freshwater^14–18^. While female mosquitoes have evolved specialized behavioral and reproductive mechanisms for host seeking, blood feeding, finding freshwater for egg laying, and egg development, males have a simpler behavioral repertoire focused on nectar feeding and mating.

Single-cell RNA sequencing (scRNA-seq) and atlasing have been instrumental in defining the molecular identity of known cell types and discovering new cell types. Cell atlases have been constructed for several whole organisms, including the roundworm *Caenorhabditis elegans*^19,20^, the planarian *Schmidtea mediterranea*^21^, the house mouse *Mus musculus*^22,23^, the gray mouse lemur *Microcebus murinus*^24^, and others. The Fly Cell Atlas represents a whole-organism cell atlas for *Drosophila melanogaster*^25^ and it has been an important resource for understanding insect cell types and gene expression patterns.

Prior studies have used bulk RNA sequencing (RNA-seq) to profile diverse mosquito tissues^26–32^. Recently scRNA-seq and single-nucleus RNA sequencing (snRNA-seq) has been used to profile different mosquito tissues, including the testes^33,34^, gut^35–39^, immune system^40–42^, olfactory organs^43,44^, brain^45^, fat body^39^, and larval ventral nerve cord^46^. All mosquito single-cell studies to date focused on a specific tissue or cell type, mainly in females. A global gene expression map with a larger number of tissues, spanning both sexes is urgently needed to allow systematic comparison and to produce unique insights^25,47^.

We sought to gain systems-level insights into the molecular and cellular differences underlying the extraordinary sexual dimorphism of this species. To achieve this, we developed the Mosquito Cell Atlas, a large-scale snRNA-seq project characterizing every major tissue from the adult female and male *Aedes aegypti* mosquito. To construct this atlas, we profiled 367,745 nuclei from 19 tissues, providing cellular resolution of the entire mosquito transcriptome. For the female brain, we include multiple timepoints before and after blood feeding, correlated with distinct phases of egg development and post blood feeding behavioral changes, to discover transcriptional changes correlated with behavioral shifts linked to reproductive state. We found specialized protein expression patterns and novel antimicrobial peptide-expressing cells in the female salivary glands. In the antennae, we discovered male-specific *ppk317*-expressing cells and sexually dimorphic olfactory sensory neurons. We observe that mosquito legs and proboscis house polymodal sensory neurons that co-express receptors for different sensory modalities and, similar to other sensory appendages, across gene families as shown in the antenna in previous studies^43,44^. In the brain, we identified sexually dimorphic gene expression in Kenyon cells and extensive transcriptional changes in glial cells following blood feeding.

This atlas represents a valuable resource for the vector biology community, bridging the gap between model organism studies and mosquito-specific biology. We hope that the mosquito cell atlas will spur interest from scientists working in other species who wish to gain comparative insights into its unique biology. While a century of work with *Drosophila melanogaster* flies has provided foundational knowledge of insects, creating the tools and datasets directly related to mosquitoes allows us to move away from homology-based research that seeks to align mosquito and fly biology. More broadly, these data offer new avenues for studying the molecular biology underlying the specific adaptations and specializations that make mosquitoes such effective and deadly pathogen vectors.

## Results

### snRNA-seq mapping of the entire adult female and male *Aedes aegypti* mosquito

**Figure 1.**
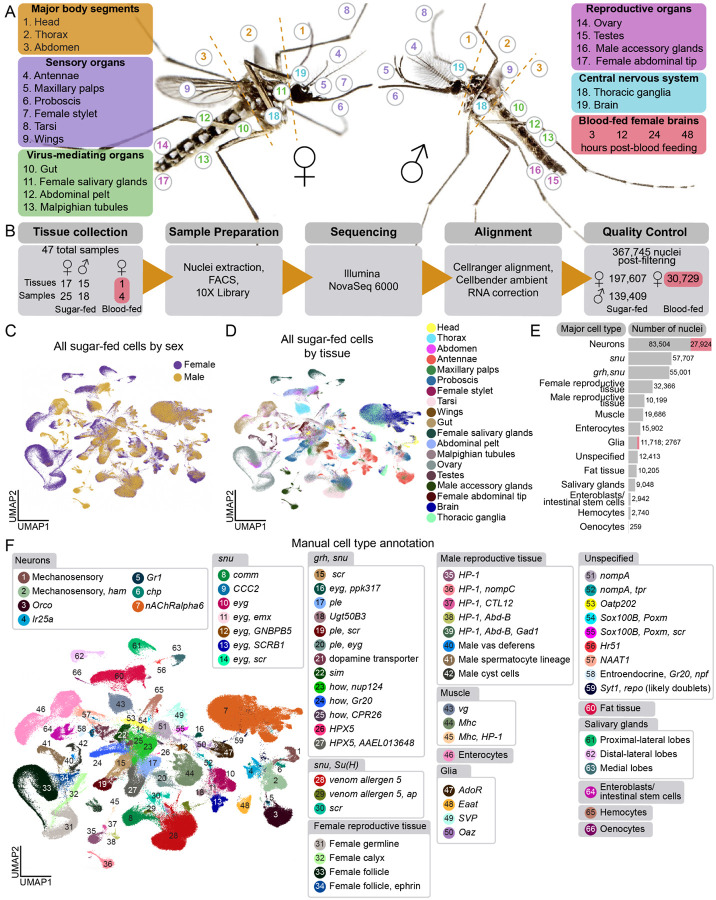
Mosquito Cell Atlas tissues and data. **(A)** Photos of *Aedes aegypti* female (left) and male (right). Numbers indicate location of collected tissues (listed in legend boxes). Photos by Alex Wild. **(B)** Schematic of Mosquito Cell Atlas workflow. **(C-D)** Uniform manifold approximation and projection for dimension reduction (UMAP) of nuclei from all sugar-fed tissues from both sexes colored by sex (C) and tissue (D). **(E)** Number of sugar-fed nuclei (gray) and blood-fed nuclei (red) collected across all samples for each major cell type, sorted by abundance. **(F)** UMAP of nuclei from all sugar-fed tissues, colored and numbered by manual annotation of major cell types as listed in legend at the right of the figure panel. For a complete look up table of gene symbols to gene IDs used in this manuscript, see Table S3. For annotation thresholds see Table S4.

Tissues for the Mosquito Cell Atlas (MCA) were selected based on biological importance and feasibility to dissect with the aim of mapping all female and male cell types from sugar-fed, aged-matched animals. In addition, we collected brains from females at various times following a blood meal to profile gene expression changes across the reproductive cycle. Our approach used physical dissection to obtain tissue for snRNA-seq, rather than generating genetically-labeled strains and isolating cells by fluorescent marker expression. This somewhat limited our ability to finely subdivide the mosquito into the largest number of individual tissues and organs. For example, we did not obtain separate data from tissues such as the heart, male salivary gland, or hemocytes. This is a common limitation in dissection-based whole animal single cell atlases. Collecting major body segments – head including head appendages, thorax including legs but not wings, and abdomen – allowed verification of gene expression signatures for individual tissues and cells from tissues not separately dissected. We chose 19 tissues related to five broad categories of mosquito biology: (I) major body segments, (II) sensation and host seeking, (III) viral infection, (IV) reproduction, and (V) central nervous system (Figure 1A). Since isolating intact cells from cuticular tissues such as antennae and maxillary palps is challenging in both mosquitoes and flies^25,43^, snRNA-seq rather than scRNA-seq was chosen for all samples for consistency.

Female mosquito egg development requires a blood meal and these animals suppress their host seeking and biting behavior for several days after the blood meal until the eggs are laid^14–18^. Previous mosquito RNA-seq studies identified hundreds of gene expression changes associated with blood feeding in many tissues, including the brain^27,29,48–51^. To assess how transcripts in the brain change after a blood meal with single-cell resolution, we sequenced female brains obtained from animals 3, 12, 24, and 48 hours post-blood feeding (Figure 1A). We selected these timepoints to represent the various stages of egg maturation and suppression of attraction to humans.

We dissected 44 samples from 17 sugar-fed female tissues, 15 sugar-fed male tissues, and 4 samples of brains from blood-fed females at the timepoints indicated above. Nuclei were extracted and collected using fluorescence-activated cell sorting (FACS), and single-nucleus transcriptomes were generated using 10X Genomics technology and Illumina sequencing unless otherwise stated (Figure 1B). Because data collection methods were identical, we included snRNA-seq data from female antenna and maxillary palp samples previously published in Herre, Goldman *et al*.^43^. Data from all samples were aligned to the *Aedes aegypti* L5 genome^52^ and individually assessed for quality control and filtered appropriately (Figure S1). We retained a total of 367,745 nuclei comprising 197,607 sugar-fed female nuclei, 139,409 sugar-fed male nuclei, and 30,729 blood-fed female nuclei from a total of 47 samples, 44 dissected for this study and 3 samples from our previous study^43^ (Figure 1B–1C and Table S1). We combined male and female samples of the same tissue to compare tissues between sexes (Table S2). We then combined the data from all male and female sugar-fed tissues to create a complete mosquito cell atlas (Figure 1D).

The hallmark of a cell atlas is the ability to annotate distinct cell types. In non-model organisms, the lack of knowledge of expected cell types or established gene markers makes annotation challenging. We developed two complementary strategies to address these challenges. First, we relied on experts in mosquito biology and entomology to annotate data using published work and validated *Aedes aegypti* gene markers wherever possible. Second, we took an unbiased approach and computationally identified gene markers using standard differential gene expression tools in scRNA-seq data analysis^53,54^. In cases when marker genes were not characterized, we relied on orthology information from genes studied in *Drosophila melanogaster*, assessed using Ensembl Metazoa BioMart database^55^, BLAST (nucleotide or protein)^56^, or Vectorbase^57^. Many of our annotations use marker genes that may imply function based on *Drosophila melanogaster* literature (list of gene identifiers and ortholog names used can be found in Table S3). *Aedes aegypti* and *Drosophila melanogaster* are separated by 260 million years of evolution^58,59^, with distinct behaviors, life cycles, and physiology. Relying on *Drosophila melanogaster* homology to interpret *Aedes aegypti* genes can be problematic. We caution that in most cases, the orthologous gene function between *Drosophila melanogaster* and *Aedes aegypti* has not been confirmed. For annotation of cell types, we sought to use multiple orthologous genes and/or genes predicted to encode a protein directly related to the function of the cells. However, to avoid mischaracterizing a cell type, we often used gene names to annotate cell types in our data. This was done to avoid the pitfall of presuming *Drosophila melanogaster* cell-type orthology from gene orthology.

We integrated data across sexes and tissues, and annotated and identified global cell types (Figures 1E, 1F, andS2). Using combinations of marker genes, we discerned 66 distinct cell types (Figure 1F and S2C) and assigned them to one of 14 major cell-type categories (Figures 1E, S2B, and S2C). Annotating tissues individually offered a higher level of specificity in tissue cell types (Figures 2, 3, 7, S2–S12, S16, S19–S20, see Table S4 and Zenodo Supplemental Data for gene thresholds and scripts used for annotation). All data and annotations are available on the UCSC Cell Browser^60^ (http://mosquito.cells.ucsc.edu) to enable community use of the data.

### Annotation of male testes and identification of spermatids

**Figure 2.**
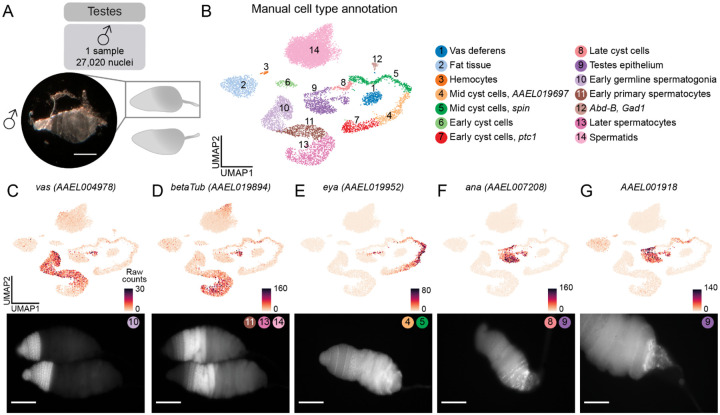
Localization and validation of testes RNA transcripts. **(A)** Photo of a dissected testis with anatomical diagram of testes pair, and collected sample information. Scale bar: 500 μm. **(B)** UMAP of testes nuclei, colored and numbered by manual cell-type annotation as listed in legend at the right of the figure panel. For a complete look up table of gene symbols to gene IDs used in this manuscript, see Table S3. For annotation thresholds see Table S4. **(C-G)** UMAP of raw counts (UMI) of a subset of genes used to annotate testes data (normalized gene expression are shown in Figures S13I–S13M), as well as corresponding validation using RNA *in situ* hybridization with probes against the indicated genes (below). Genes include *vas* (*AAEL004978*) (C), *betaTub* (*AAEL019894*) (D), *eya (AAEL019952*) (E), *ana (AAEL007208)* (F), and *AAEL001918* (G). Corresponding cell type(s) (number and color from B) are shown at top right of each RNA *in situ* hybridization image. Scale bar: 100 μm for C, E-G; 50 μm for D.

To validate the quality of our snRNA-seq data and our annotation approach, we first turned to the male testes, a tissue studied extensively as a potential target for mosquito population control and that has well-characterized cell types and marker genes. We dissected testes from 212 male mosquitoes for a total of 27,020 nuclei after quality-control filtering (Figure 2A). Following pre-processing, we identified 14 distinct cell types (see Figure 2B, Table S4 and Zenodo Supplemental Data for gene thresholds and scripts used for annotation).

We first identified the germline lineage based on the expression, in early stages, of *vas* (*AAEL004978*), which is homologous to the *Drosophila melanogaster* germline marker and known for its conserved role in germ cell development across species^61^ (Figure 2B), and expression of *beta2*-*tubulin* (*AAEL019894*) in spermatocytes. Initially, spermatids were absent from our analysis, due to their characteristically low transcriptional activity^25,62,63^ (Figure S13A–S13D). After modifying our filtering criteria, we identified a distinct spermatid cluster characterized by the presence of *S*-*Lap* (*AAEL000108*), *DBF4* (*AAEL008779*)^64^, and *Orco* (*AAEL005776*)^65^ (Figures 2B, S13E–S13G). Similarly, we observed clusters representative of the various stages of cyst cell development including early to late cyst cells, as well as the testes epithelium (Figure 2B). Intriguingly, we also observed expression of the taste receptor *Gr39* during the cyst cell developmental trajectory, underscoring a potential role of chemoreceptors in non-sensory tissues (Figure S13H)^66^.

Next, we used RNA fluorescence *in situ* hybridization to correlate mRNA expression patterns in testes with our snRNA-seq data. Consistent with our snRNA-seq, *vas* (*AAEL004978*) RNA *in situ* hybridization showed expression in early germline cells, from germline stem cells to early spermatocytes (Figure 2C). The expression of testis-specific *beta2*-*tubulin* (*AAEL019894*) was mainly detected in primary spermatocytes (Figure 2D), consistent with a previous study in *Aedes aegypti*^67^. Weaker *beta2*-*tubulin* RNA *in situ* hybridization signal can be seen in spermatids, as the transcripts remain in the cytoplasm of early elongation spermatids before being degraded as the spermatids mature^68^.

*eya* is a well-characterized cyst cell marker in *Drosophila melanogaster*^69^. According to our snRNA-seq data, the *Aedes aegypti* homolog of *eya (AAEL019952)* is also expressed in cyst cells, particularly at the mid-stages of spermatogenesis (Figure 2E). RNA *in situ* hybridization of *eya (AAEL019952)* confirms this localization. We also observed regional differences in the testicular epithelium. *ana* (*AAEL007208*) was detected in the testes epithelium, particularly towards the posterior of the testis, and in late cyst cells (Figure 2F). *AAEL001918* was also detected in the terminal epithelium and was enriched in the most posterior region (Figure 2G). These findings are consistent with the snRNA-seq data and also suggest the existence of a transcriptomically-distinct terminal subpopulation of the testes epithelium.

Coordinated results between our RNA *in situ* hybridization experiments and snRNA-seq data supported our cell annotation approach, which we then applied to all other tissues in this project.

### Enhanced spatial mapping of infection-related genes in the female salivary gland

**Figure 3.**
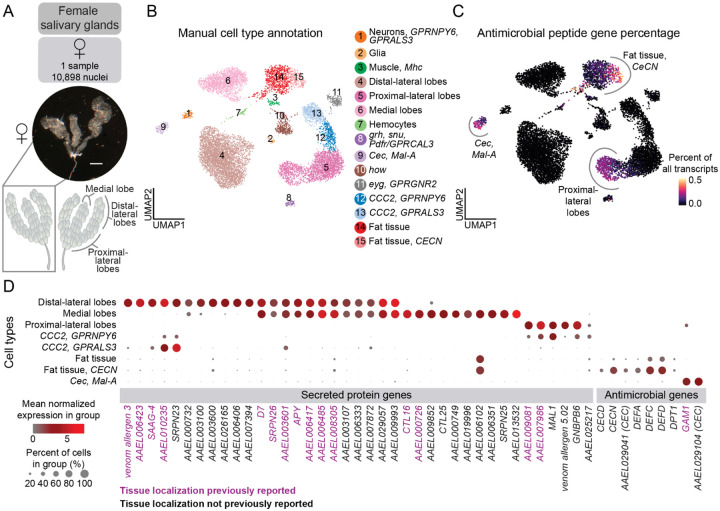
Localization and validation of female salivary gland RNA transcripts. **(A)** Photo of a dissected female salivary gland (middle) with labeled anatomical diagram (bottom), and collected sample information (top). Scale bar: 500 μm. **(B)** UMAP of female salivary gland nuclei, colored and numbered by manual cell-type annotation as listed in legend at the right of the figure panel. For a complete look up table of gene symbols to gene IDs used in this manuscript, see Table S3. For gene annotation thresholds see Table S4. **(C)** Female salivary gland UMAP, colored by expression of antimicrobial peptides set: *CECD* (*AAEL029046*), *CECN* (*AAEL029047*), putative cecropins (*AAEL029041* and *AAEL029104*), *DEFA* (*AAEL003841*), *DEFC* (*AAEL003832*), *DEFD* (*AAEL003857*), *DPT1* (*AAEL004833*), *GAM1*(*AAEL004522*). Expression of gene set shown as a fraction of total transcripts in each cell (color bar trimmed 0.1% for visibility). Relevant cell types labeled. **(D)** Dot plot illustrating mean normalized expression of salivary gland secreted protein genes and antimicrobial genes by cell type. Localization of genes colored in purple has been validated by previous work^88^. Normalized expression is ln([(raw count/total cell counts)x median total counts across cells]+1). For more information on salivary gland protein genes, see Table S4.

Mosquito salivary glands are critical for pathogen transmission because female mosquitoes inject saliva beneath the skin during blood feeding. Secretory salivary components influence the host immune response and reduce pain sensitivity to allow the mosquito to feed to repletion before being detected by the host^70–73^. These salivary components are also vital for pathogen transmission^73–85^. The paired salivary glands are divided into three lobes, with the proximal-lateral and distal-lateral lobes flanking one medial lobe (Figure 3A). A basal lamina surrounds each lobe, and a single layer of epithelial or saliva-secretory cells (acinar cells) is found within the lamina. These cells are arranged around a central salivary duct that has an apical cavity for saliva storage^86–88^.

We dissected the paired salivary glands from 495 female mosquitoes and obtained data from 10,898 nuclei after quality-control filtering (Figure 3A). Recent single-cell transcriptomics, proteomics, and genomics studies have profiled salivary glands from blood-feeding insects leading to a comprehensive list of salivary gland marker genes^74,89–96^, which we used to manually annotate the salivary gland tissue cell types in our data (Table S4, Zenodo Supplemental Data). All known salivary gland lobes and expected cell types were recovered in our dataset (Figure 3B).

Previous work has used RNA *in situ* hybridization and immunofluorescence to localize expression of a number of salivary proteins to specific subsets of cells^88,97–99^. We found that a majority of genes related to saliva proteins localized to the three lobes (Figure 3D). We cross-referenced these to published *in situ* hybridization data^88^, validating our annotations, and demonstrating our ability to effectively classify the saliva-secretory cells within each of the three major saliva-producing regions. Next, we used our data to identify the putative localization of all secreted proteins identified by mass spectrometry from previous studies of *Aedes aegypti* salivary glands^89,100,101^ (Table S4). Using gene expression as a proxy, we successfully assigned the localization of 24 secreted proteins whose cell type and tissue localization was not previously reported (Figure 3D).

Antimicrobial peptide genes are involved in mosquito innate immunity and host response to pathogens^84^ and are relevant to mosquito population and disease control. In *Drosophila melanogaster*, fat body cells synthesize antimicrobial peptides for secretion into hemolymph^102^. We found antimicrobial peptide genes, including cecropins (*CECN*) and defensins (*DEFA, DEFC, DEFD*), in the fat tissue collected as part of our dissections of salivary gland and the abdominal pelt, as well as in other cell types throughout the mosquito, including enterocytes and intestinal stem cells (Figure 3C, and S14A–S14E).

These comprehensive data provide a better understanding of the expression patterns of salivary-secreted protein genes and antimicrobial peptides. Transcriptomic access to these critical cells that mediate the spread of disease may stimulate new avenues of investigation into viral transmission and vector effectiveness.

### *ppk317* labels a previously unknown male-specific cell type in the antenna

**Figure 4.**
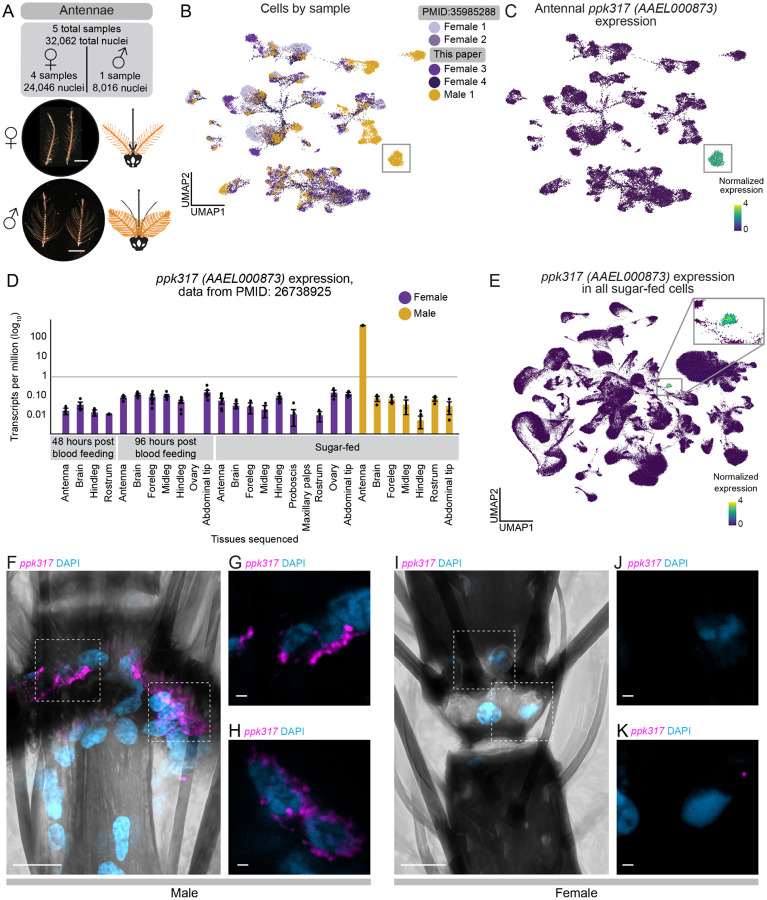
Identification of male-specific *ppk317* cell type in the *Aedes aegypti* antenna. **(A)** Photo of dissected female (middle) and male (bottom) antenna with anatomical diagram (in orange, bottom-left), and collected sample information (top). Scale bar: 500 μm. **(B)** UMAP of antenna nuclei, colored by sample (female samples = 4, male samples = 1). Putative male-specific cluster highlighted in gray box. **(C)** UMAP of *ppk317* (*AAEL000873*) gene expression (normalized) in all antenna nuclei. Cluster with high expression highlighted in gray box. Normalized expression is ln([(raw count/total cell counts)x median total counts across cells]+1). **(D)** *ppk317 (AAEL000873)* expression [transcripts per million (log_10_)] in the indicated female tissues 48 and 96 hours post-blood feeding or fed on sugar (purple) and sugar-fed male (yellow) tissues. Females were not offered an egg-laying substrate prior to tissue collection. Data from previously published RNA-seq data^27^. **(E)** UMAP of *ppk317 (AAEL000873)* normalized expression in all sugar-fed nuclei. Cluster with high expression highlighted in gray box, enlarged in inset. **(F)** Maximum-intensity projection of whole-mount male antenna with RNA *in situ* hybridization showing *ppk317* probe (magenta) and nuclear staining (DAPI). Scale bar: 10 μm. **(G-H)** Enlarged view of a single Z plane (Z = 0.24 μm) of white highlighted boxes from (F) left box **(G)** and right box (H). Scale bar: 1 μm. **(I)** Maximum-intensity projection of whole-mount female antenna with RNA *in situ* hybridization showing *ppk317* probe (magenta) and nuclear staining (DAPI). Scale bar: 10 μm. **(J-K)** Enlarged view of a maximum-intensity projection of white highlighted boxes from (I) left box (J) and right box (K). Scale bar: 1 μm.

Female mosquitoes are exquisitely sensitive to the smell of human hosts and use chemosensory neurons in their antennae to detect human body odor^8,103–107^. While the cellular, molecular, and neuronal composition of the female antenna has been extensively investigated^43,44,108–113^, the biology of the male antenna is largely unexplored. Male mosquitoes are not attracted to human odor cues^114^ and mosquito antennae are strikingly sexually dimorphic (Figure 4A). The cellular and genetic basis of these structural and behavioral dimorphisms is unknown.

To understand differences in cellular composition, we sequenced one male and two female antenna samples and integrated them with our previously published female antenna samples^43^ for a total of 24,046 female nuclei and 8,016 male nuclei (Figure 4A–4B). Visualization of the data on a common UMAP coordinate revealed shared and sex-specific sub-populations (Figures 4B and S15D). While the presence of separated and shared sub-populations between the sexes indicates differences that are likely biological, we cannot rule out complete absence of technical or batch effect given differences in sample processing (Figure S15C). Because batch correction methods aggressively merge the samples, thereby eliminating any biological differences^115^, we sought to take a more cautious approach in identifying specific markers or cell types to investigate cellular differences between underlying antenna sexual dimorphism. (Figure S15C–S15D).

We focused on a non-neuronal cluster of male-specific cells marked by *ppk317 (AAEL000873)*, an ion channel of unknown function belonging to the *pickpocket* (PPK) channel (DEG/ENaC) gene family (Figures 4B–C and S15D)^116–118^. Bulk RNA-seq studies have demonstrated that *ppk317* is exclusively expressed in the male antenna^27^ (Figure 4D). Investigating our integrated snRNA-seq dataset of all 330,364 nuclei in the sugar-fed mosquito, showed that *ppk317* is only present in nuclei from the male antenna and head (Figures 4E and S15H–S15I). We find that *ppk317* expression is highly unique to a single male-specific cell type in the antennae.

Male-specific *ppk317* cells are likely epithelial-related, based on their expression of *grh*, an orthologue of a *Drosophila melanogaster* epithelial cell marker^25,119^. To ask to which degree male *ppk317* cells differ from other antenna cell types, we carried out diffusion component, partition-based graph abstraction to quantify transcriptomic distances between clusters, and gene-expression correlation analyses to assess expression heterogeneity within and between clusters. We quantitatively demonstrate a strong difference between the male *ppk317* cluster and other cell types, including other *grh*+ and *grh+, snu*+ cells (Figures S15E–S15G, S15J, and Zenodo Supplemental Data). *ppk317* cells were also strongly self-correlated based on their gene expression, indicating relative homogeneity within the cell type (Figure S15G, S15J). To confirm that *ppk317* is only expressed in male antennae, we performed RNA *in situ* hybridization on male and female antennae*. ppk317* showed strong and selective expression in the joints of the male antennae (Figure 4F–4G) and was not detected at all in female antennae (Figures 4I–4K, S16A). The function of this unique male-specific *ppk317* cell type is unknown and requires future investigation.

### A precise sexual dimorphism in a single antennal chemosensory cell type

Understanding the complexity of the mosquito olfactory system is crucial to deciphering how mosquitoes excel at locating human hosts. Insects detect chemosensory cues with heteromultimeric ligand-gated ion channels encoded by three large multigene families, the odorant receptors (ORs), ionotropic receptors (IRs), and gustatory receptors (GRs). These receptors assemble into complexes composed of broadly expressed co-receptors and more selectively expressed ligand-specific subunits. Recent work using snRNA-seq and other methods showed that female *Aedes aegypti* olfactory sensory neurons co-express both co-receptors and ligand specific receptors both within and between major receptor families^43,44^. Using data from our cell atlas, we investigated if the organization of the *Aedes aegypti* male antenna resembles that of females and if receptor co-expression occurs.

**Figure 5.**
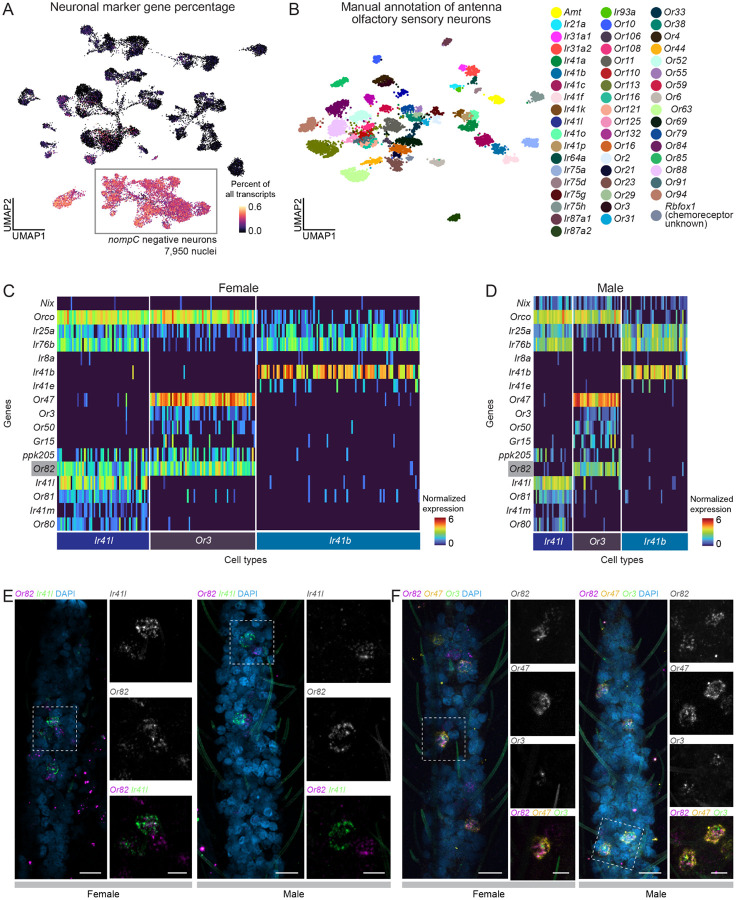
Precise sexually-dimorphic expression of *Or82* in a single antennal chemosensory cell type **(A)** Normalized expression UMAP of antenna neuronal genes set *Syt1* (*AAEL000704*), *brp* (*AAEL018153*), *nSyb* (*AAEL024921*) and *CadN* (*AAEL000597*). Expression of gene set shown as a fraction of total transcripts in each cell. *nompC* (*AAEL019818*)-negative cells highlighted by gray box (for *nompC* gene percentage, see Figure S17B). Normalized expression is ln([(raw count/total cell counts)x median total counts across cells]+1). **(B)** UMAP of antenna *nompC*-negative (olfactory sensory) neurons after filtering, colored by manual cell-type annotation as listed in legend at the right of the figure panel. For filtering steps and parameters, see Figure S17, Table S2, and Zenodo Supplemental Data. For a complete look up table of gene symbols to gene IDs used in this manuscript, see Table S3. For annotation thresholds see Table S4. **(C-D)** Heatmap of cells from female (C) and male (D) samples from annotated clusters *Ir41l, Or3*, and *Ir41b*. Selected genes are indicated in rows and cells indicated in columns. Cell types indicated below heatmap. Heatmap colors represent normalized expression. **(E)** Maximum-intensity projection of whole-mount female and male antennae with RNA *in situ* hybridization of *Or82* (magenta), *Ir41l* (green), and DAPI nuclear staining (blue). Scale bar: 10 μm. Right side of each large image: enlarged view of highlighted white box on left, with the indicated probes. Scale bar: 5 μm. **(F)** Maximum-intensity projection of whole-mount female and male antennae with RNA *in situ* hybridization of *Or82* probe (magenta), *Or47* (yellow), *Or3* (green) and DAPI nuclear staining (blue). Scale bar: 10 μm. Right side of each large image: enlarged view of highlighted white box on the corresponding left side, with the indicated probes. Scale bar: 5 μm.

We began by isolating neurons among the total population of 32,062 male and female antenna nuclei (Figure 4A–4B) based on expression of *Syt1* (*AAEL000704*), *brp* (*AAEL018153*), *nSyb* (*AAEL024921*) and *CadN* (*AAEL000597*) (Figure 5A). Mechanosensory neurons comprising 9% of total neurons based on the expression of the *Drosophila melanogaster* orthologue of mechanosensory receptor *nompC* (*AAEL019818*) were filtered out to focus on non-mechanosensory neurons (Figure S17A–S17B). After additional quality control filtering steps, we obtained 7,950 *nompC*-negative neurons comprising 7,003 neurons from females and 947 neurons from males (Figures 5A and S17C–S17I).

We reclustered the *nompC*-negative neurons and manually annotated them using chemoreceptors uniquely expressed within a cell type (Figure 5B). As previously reported^43,44^, *Aedes aegypti* olfactory sensory neuron cell types can express multiple ligand-specific chemoreceptors. Some ligand-specific chemoreceptors are also co-expressed in more than one olfactory sensory neuron cell type, as part of different sets of ligand-specific chemoreceptors. Generally, our annotated cell types demonstrated unique combinations of putative transcription factor genes, supporting our annotation’s classification of transcriptomically distinct cell types (Figure S18A). The two new female antennal samples replicated our previous finding of co-expression of *Orco* and *Ir25a*^43^ (Figures 5C–5D and S17L). Our data also replicate prior findings that female *Ir41l*-expressing olfactory sensory neurons co-express *Orco*, *Ir25a*, and *Ir76b*, along with additional ligand-specific receptors including *Or80, Or81*, *Or82*, and *Ir41m*^43,44^ (Figure S17L). We additionally find that *Ir41l* olfactory sensory neurons also express *ppk205* (Figure 5C–5D).

In total, we annotated over 54 olfactory sensory neuron cell types with distinct expression patterns (Figure S18 and Zenodo Supplemental Data). In our annotations, we found at least 6 examples of chemoreceptor genes co-expressed within a cluster but not within the same cells. While this indicates mutual exclusivity of the genes, we cannot rule out the possibility that it could be due to dropouts in single-cell sequencing, particularly because receptor genes can be expressed at relatively low levels, although this is unlikely given the large number of cells profiled at a high sequencing depth here. Furthermore, these cells occupy the same phenotype space and are not discernible as distinct clusters computationally, suggesting that these olfactory sensory cell types may be distinct, but transcriptomically similar (for instance, *Ir41b* and *Ir41*e in Figures 5C–5D, S18B, and Zenodo Supplemental Data). These findings are similar to the ~60 olfactory sensory neuron cell types recently identified in the antenna in another study^44^.

We then investigated the differences between male and female mosquito olfactory sensory neurons. Despite the sexually dimorphic olfactory behaviors displayed by male and female mosquitoes^13,114,120,121^, there were limited transcriptional differences between male and female olfactory sensory neurons. This is reflected in our finding that all annotated cell types contained both male and female cells, although in different proportions (Figure S17I–S17J). We then calculated differentially expressed genes between male and female samples within each sensory neuron cell type using MAST^54^ (Figure S17M and Table S5). Across the sensory neuron cell types, the ADP/ATP carrier protein *SLC25A5* (*AAEL004855*) was the most frequent differentially-expressed gene between female and male chemosensory cells, followed by a putative Mg^2+^/Na^+^ transporter (*AAEL009150*), the male-determining factor *Nix* (*AAEL022912*), a putative serine/threonine kinase (*AAEL004217*), and the odorant binding protein *OBP35* (*AAEL002606*) (Table S5). The significance of this sex-specific differential expression is unknown, with the exception of *Nix*, which is required for male sex determination.

Out of 403 putative sensory genes queried, including ORs, IRs, GRs, PPKs, transient receptor potential (TRP) ion channels, opsins, and mechanosensory receptors, only four were significantly differentially expressed between corresponding male and female sensory neuron cell types: *Or82*, *Ir25a*, *Ir76b*, and *Or2* (Figure S17N–S17Q, Table S5, for full queried gene list see Table S4). Surprised to find so few differentially-expressed sensory genes, we wanted to ensure that our results were not driven by outliers and effects of data normalization. We looked at the raw counts (unique molecular identifiers) of all of the sensory genes in the olfactory sensory neuron population (Figure S18C). We found comparable gene transcript counts for each sample and across sexes, validating that we were not overlooking large differences between male and female sensory gene expression.

Careful analysis of these chemoreceptor expression profiles revealed a precise and unexpected sexual dimorphism in a single population of olfactory sensory neurons. We discovered that while male and female *Ir41l* olfactory sensory neurons have the same chemoreceptor expression profile and co-express the same ensemble of OR and IR family co-receptors and ligand-specific receptors, male *Ir41l* olfactory sensory neurons do not express *Or82* (Figures 5C–5D and S17N). This sexually dimorphic expression of *Or82* is limited to *Ir41l* olfactory sensory neurons because both male and female *Or3* olfactory sensory neurons express *Or82* (Figure 5C–5D). To confirm that *Or82* is female-specific in *Ir41l* cells and absent from male *Ir41l* cells, we performed multiplex fluorescent RNA *in situ* hybridization on antennae with *Or82* and *Ir41l* probes. As our snRNA-seq data predicted (Figure 5C–5D), *Or82* is expressed and co-localizes with *Ir41l* in females but does not co-localize with *Ir41l* in males (Figure 5E). We detect *Or82* in male antennae (Figure 5C–5D), and find that *Or82* is co-localized with *Or47* and *Or3* in both female and male antennae (Figure 5F) as predicted by our snRNA-seq data. The ligand profile of *Or82* is unknown and it is not known what consequence, if any, the lack of *Or82* in male *Ir41l* olfactory sensory neurons has for dimorphic olfactory behaviors of male and female mosquitoes. This work underscores the striking similarity in chemoreceptor expression between male and female olfactory sensory neurons, with one notable exception.

### Molecular signature of polymodal sensory detection in leg sensory neurons

**Figure 6.**
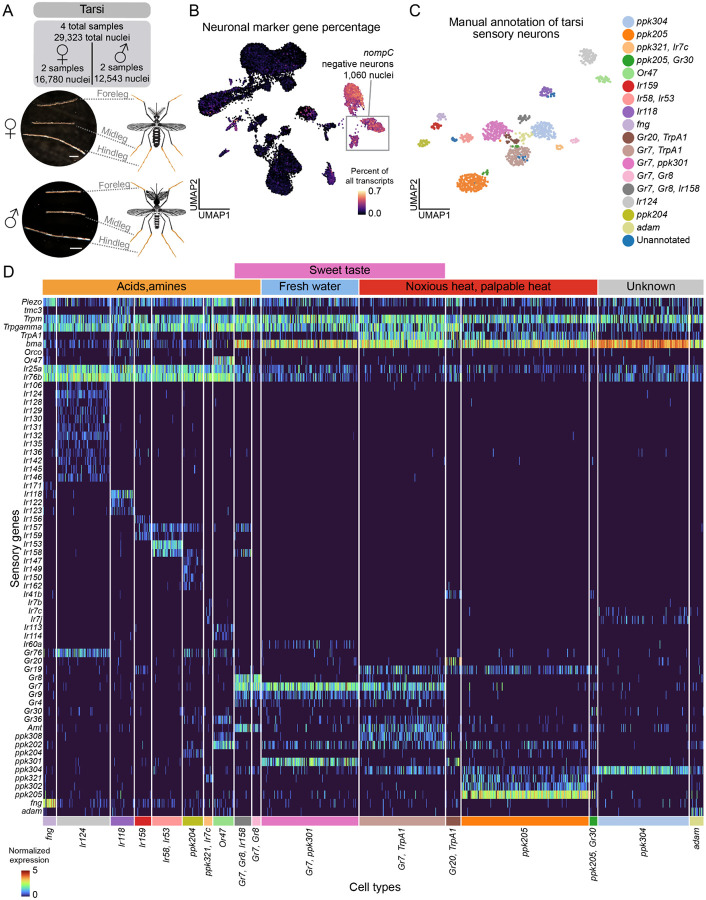
Tarsi sensory neurons are polymodal. **(A)** Photo of dissected female (top) and male (bottom) tarsi, (bottom) with anatomical diagram (in orange; labeling the foreleg, midleg and hindleg), and collected sample information. Data was collected from the three most distal segments of the tarsi. Scale bar: 500 μm. **(B)** Normalized expression of tarsi neuronal genes set: *Syt1* (*AAEL000704*), *brp* (*AAEL018153*), *nSyb* (*AAEL024921*) and *CadN* (*AAEL000597*). Expression of gene set shown as a fraction of total transcripts in each cell. *nompC* (*AAEL019818*)-negative cells highlighted by gray box. Normalized expression is ln([(raw count/total cell counts)x median total counts across cells]+1). For *nompC* gene percentage, see Figure S22A. **(C)** UMAP of tarsi chemosensory (*nompC*-negative) neurons after filtering, colored by manual cell-type annotation as listed in legend at the right of the figure panel. For filtering steps, see Table S2 and Zenodo Supplemental Data. For a complete look up table of gene symbols to gene IDs used in this manuscript, see Table S3. For annotation thresholds see Table S4. **(D)** Heatmap of cells from all annotated clusters. Sensory genes are indicated in rows and cells indicated in columns. Cell types indicated below heatmap and respective sensory function indicated above. Heatmap colors represent normalized expression.

*Aedes aegypti* legs enable mosquitoes to evaluate human skin before blood feeding^122^, detect pheromones during mating^123–125^, and identify suitable egg-laying sites in freshwater^118,126^. They can detect multiple stimuli including osmolality^118^, bitter substances^127,128^, sugars^129^, and amino acids^130^. Mosquitoes have three pairs of legs: forelegs, midlegs, and hindlegs. We collected snRNA-seq data from the most distal segment of the leg, the tarsi, which contains most of the leg’s neuronal cell bodies, including mechanosensory and chemosensory neurons^11,131,132^ from 332 and 298 female and male mosquito legs, respectively, yielding a total of 29,323 tarsal nuclei (Figure 6A). We identified 1,060 *nompC*-negative neurons, excluding putative mechanosensory neurons (Figures 6B and S19C). Clustering *nompC*-negative sensory neurons revealed cell types with mostly distinct receptor gene profiles (Figure 6C–6D and Zenodo Supplemental Data).

Subpopulations of tarsal sensory neurons showed co-expression of different receptor families. *ppk204* cells express the IR co-receptors (*Ir25a* and *Ir76b*) and ligand-specific genes. Co-expression of IRs and PPKs has also been observed in *Drosophila melanogaster* tarsi snRNA-seq data^133^. *Ir124* cells express several other IR ligand-specific genes as well as *Gr76. Or47* cells co-express *ppk202*, *Gr36*, *Gr76*, *Ir113*, and *Ir114*. Interestingly, although antennal *Or47* cells co-express *Orco* as expected (Figures S5C–S5D and S18B), tarsal *Or47* cells do not co-express *Orco*, raising the question of whether *Or47* functions in tarsi without what is assumed to be its obligate co-receptor (Figure 6D). We note that GR-expressing cell types also have some expression of the IR co-receptors *Ir25a* and *Ir76b*, although at lower normalized expression values and more sparsely than cell types that express ligand-specific IRs (Figure 6D). In the proboscis, we observed co-expression of *Orco* and *Or47*, with IR co-receptors and a diversity of ligand-specific IRs (Figure S21C and Zenodo Supplemental Data). These data show that mosquito neurons in the tarsi, as well as the proboscis (Figure S21), can co-express chemosensory receptors across gene families.

Remarkably, tarsal taste neurons also co-express receptors known to operate in distinct sensory modalities including taste, heat, and osmolality, suggesting that some tarsal sensory neurons are polymodal. The low-salt detector *ppk301* enables *Aedes aegypti* females to detect freshwater, helping them avoid laying eggs in toxic high-salt environments^118^. We found that *ppk301* co-expresses with the sweet taste receptors *Gr7* and *Gr9* (Figure 6D). Interestingly, although *ppk301* co-expresses with *Gr7* in the tarsi (Figure 6D), these genes are expressed in separate cell types in the proboscis (Figure S21C), suggesting that mosquitoes may employ receptors combinatorially in different appendages for different sensory coding functions.

Both *Gr7* and *ppk205* cells co-express *TrpA1* (*AAEL001268*), which functions in noxious heat detection^134^, suggesting these neurons may detect both sweet taste and heat (Figure 6D). *Ir140* is required for heat-related sensory compensation in *Orco* mutant mosquitoes^11^ and we found *Ir140* in a cluster with a number of IRs, in addition to *Gr76* (Figure S24E). *ppk304* and *ppk102*, putative orthologues of *Drosophila melanogaster ppk29* and *ppk23* that are required for pheromone detection in the fly^135^ are co-expressed in the mosquito (Figure S24E–S24F). Although comparatively sparser in chemoreceptor gene expression, we found a *Gr39* cell type in the wing and a cell type expressing both *Gr20* and *Gr60* in the abdominal tip (Zenodo Supplemental Data).

We investigated the tarsi, proboscis, and maxillary palp sensory neurons for possible sites of sexual dimorphism (Figures S23A–S23B and S24C). The proboscis *Ir7e* cell type was only present in female samples and the tarsal *ppk205/Gr30* cells only in male, although this is based on a small sample of 19 and 12 cells, respectively (Figure 24D). Other cell types were present in different proportions within, but not specific to, male or female samples. We looked for gene expression differences among each cluster, and found no sensory-related genes to be differentially-expressed (Figure 24C and Table S6). This further emphasizes the striking similarity between male and female sensory neurons across sensory organs. Co-expression of receptors sensitive to different sensory modalities has important implications for mosquito behavior. However, many of these genes remain uncharacterized, and future studies will need to examine the functional consequences of the distribution of these receptors within cell types.

### Sensory neurons express a cell-type specific neuropeptide receptor code

Neuropeptides serve as critical modulators of behavior and physiology in mosquitoes, modifying neural circuit function and behavioral states. In *Aedes aegypti*, over 100 predicted neuropeptides regulate diverse processes, including host seeking, blood feeding, and reproduction^18,136,137^. To ask whether sensory neurons express genes related to neuropeptide and their receptors, we queried 122 genes from these gene families. By analyzing the percentage of cells in each sensory cell type that expresses these genes, we found that while some receptors are broadly expressed in most sensory cell types (e.g. *SIFaR1*, *InR*, *GPRNPY7*, *NYPLR3)*, other receptors demonstrated a degree of specificity in their expression patterns that corresponded to the chemosensory receptor expression profiles (Figures S23 and S24A). This receptor code was observed in all sensory tissues analyzed in this study and suggests that neuropeptide signaling could modulate sensory neurons in a cell-type specific manner.

### Sexually dimorphic Kenyon cells and glia in the brain

**Figure 7.**
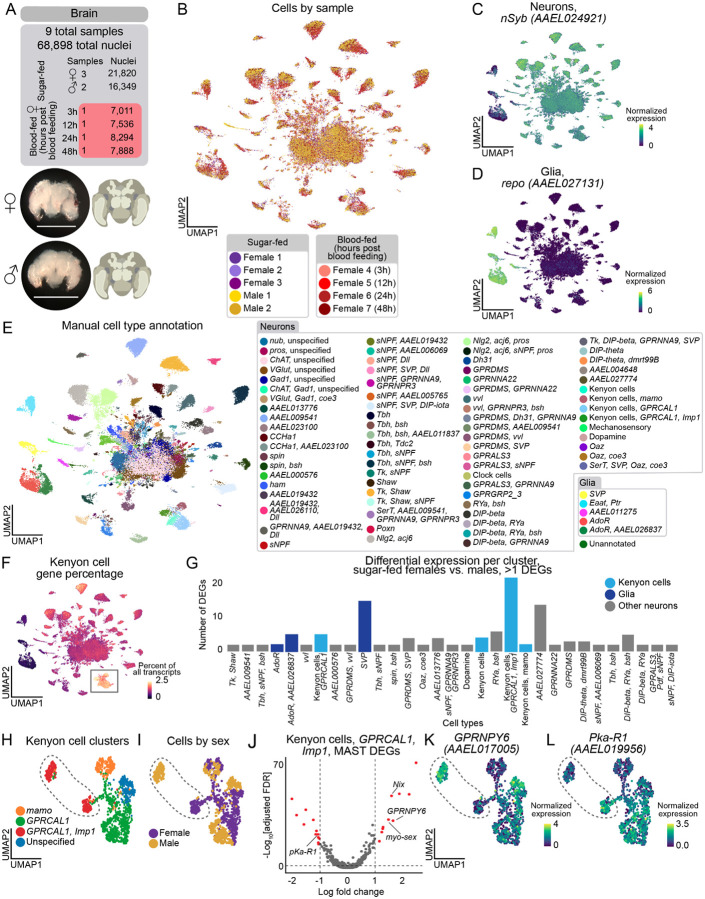
Brain annotation identifies sexually dimorphic Kenyon cells and glia **(A)** Photo of dissected female (top) and male (bottom) brain with anatomical diagram, and collected sugar-fed and blood-fed sample information. Scale bar: 500 μm. **(B)** UMAP of brain nuclei, colored by sample (female samples = 7, male samples = 2). **(C-D)** Normalized gene expression UMAP of neuronal marker, *nSyb (AAEL024921)* (C) and glial marker *repo (AAEL027131)* (D) in all brain nuclei. Normalized expression is ln([(raw count/total cell counts)x median total counts across cells]+1). **(E)** UMAP of nuclei from all samples, colored and numbered by manual annotation using marker genes, as listed in legend at the right side of figure panel. For a complete look up table of gene symbols to gene IDs used in this manuscript, see Table S3. For annotation thresholds see Table S4. **(F)** UMAP of gene percentage for a set of 30 putative Kenyon cell gene markers (Table S4). Relative expression of gene set shown as a fraction of total transcripts in each cell. Annotated Kenyon cells highlighted by gray box. **(G)** Bar plot of number of differentially expressed genes between females and males in each sugar-fed brain cell type. Clusters included contain at least 2 differentially expressed genes (DEG) with a |log fold change| > 1 and false discovery rate < 0.05, determined by MAST on normalized expression. Clusters colored by cell identity: Kenyon cells (light blue), glia (dark blue), and other neurons (grey). **(H-I)** UMAP of reclustered Kenyon cells from all sugar-fed brains, cells colored by manual cell-type annotation (H), and by sex (I). Dotted area marks sexually differential expressed Kenyon cells cluster. **(J)** Volcano plot of differentially expressed genes in the “*GPRCAL1, Imp1*” Kenyon cell cluster by sex using MAST analysis. All significant genes (indicated in red) a |log fold change| > 1 and false discovery rate < 0.05, determined by MAST on normalized expression. Male biased genes on right, as indicated by *Nix* (*AAEL022912*) and *myo*-*sex (AAEL021838)*, female biased genes on left. *pKa*-*R1* and *GPRNPY6* are labeled. **(K-L)** Normalized gene expression UMAP of *GPRNPY6 (AAEL017005)* (K) and *pKa*-*R1 (AAEL019956)* (L) in reclustered Kenyon cell nuclei from all sugar-fed brains.

Investigation of the mosquito central nervous system is crucial for better understanding of their unique and sexually dimorphic behaviors^138,139^. We collected samples from the brain (21,820 female and 16,349 male nuclei) and thoracic ganglia of the ventral nerve cord (9,306 female and 8,304 male nuclei) (Figures 7A and S7). After blood feeding, female mosquitoes exhibit unique behaviors, including decreased activity and host-seeking suppression^14–16,18,51,140^. To better understand how this may be regulated by the cells in the brain, we also collected female brain samples 3, 12, 24, and 48 hours after blood feeding (Figure 7A). This yielded a total of 68,898 brain nuclei post-quality control filtering (Figures 7A, 7B, and S25A).

The *Aedes aegypti* brain is estimated to have ~250,000 cells, of which ~220,000 are neurons^141^. In our brain data, 92% of nuclei were neurons and 8% were glia, based on the normalized expression of the markers *nSyb*, and *repo*, respectively (Figure 7C–7D). Because there is no prior literature characterizing subtypes of neurons and glia in the *Aedes aegypti* brain, we manually identified cell populations based on marker genes (Figures 7E and S25B). We note that many markers are based on ortholog information from *Drosophila melanogaster* and will require further validation.

To understand the depth of our sampling of neuronal cell types in our data, we looked for the central clock cells, a group of fewer than 15 cells in the brain of an adult mosquito^142,143^. We identified a small cluster, marked exclusively by the neuropeptide Pigment-Dispersing Factor (*Pdf*) (*AAEL001754*) and by a set of other circadian rhythm regulatory genes (Figure S26C–S26D), validating our ability to identify rare cell types.

To assist in our annotations, we compared our clustered mosquito brain data to annotated data from *Drosophila melanogaster* head^25^ using SAMap, an algorithm that iteratively matches gene homologs and cell types using graph-based data integration^144^. Analyzed separately, neurons and glia each matched with an alignment score of 0.64, compared to a score of 0.47 when we combined fly glia and mosquito neurons as a control (Figure S26E–S26G). Many clusters had high mapping scores to one or a few annotated cell types in the fly cell atlas (Figure S26H–S26J, Table S7, and Table S8). However, because of the considerable evolutionary distance and similarities between neuron types within species, mapping scores between cell types should be regarded with caution, and the lack of a strong mapping score cannot be interpreted as a lack of a corresponding cell type. We identified mosquito Kenyon cells in the mushroom body, a conserved invertebrate brain structure involved in learning and memory^145^, both through high SAMap mapping scores and expression of orthologs of *Drosophila melanogaster* Kenyon Cell gene markers (Figures 7F S26J, S26L–S26P and Table S7)^25,146^.

Using our annotated brain data, we investigated differences within each cell type between male and female brains. We calculated differentially expressed genes between males and females using MAST^54^ (Figure 7G, Table S9). Among cell types that had greater than 10 cells in each male and female condition and more than two differentially expressed genes, 28 of 72 were neuronal cell types, and 3 of 5 were glial. Among the frequently differentially expressed genes within each cell type are four genes involved in sex determination and sex-specific neuronal function: *Nix* (*AAEL022912*) and *myo*-*sex (AAEL021838)* were upregulated in male cell types, and *fru* (*AAEL024283*) and *dsx* (*AAEL009114*) were upregulated in female cell types (Table S7). Curiously, we observed that the presence of *nompC (AAEL019818)*-positive neurons (Table S7) was exclusive to male samples (Figures 7E, S25A, and S26B). Otherwise, we did not observe major differences in the abundance of cell type annotations across sexes (Figure S25A).

Among the Kenyon cells, the cluster expressing *GRPCAL1* (*AAEL010043*) and *Imp1* (*AAEL006876*) (Figure 7H–7I) showed the most sexually-dimorphic gene expression of all brain cell types (Figure 7G). In this Kenyon cell subtype, one of the most upregulated genes in males included *GPRNPY6* (*AAEL017005*), a neuropeptide Y receptor and a highly upregulated female genes included the signaling receptor *Pka*-*R1* (*AAEL019956*) (Figure 7J and 7L). Glial cells marked by *SVP (AAEL002765*) also exhibited a high number of differentially expressed genes (Figure 7J and 7K). This is consistent with recent brain snRNA-seq data from *Drosophila* species showing that glial cell types display more divergent gene expression profiles than neuronal cell types^147^.

### Glial cells display dramatic transcriptional changes in the female brain after blood feeding

**Figure 8.**
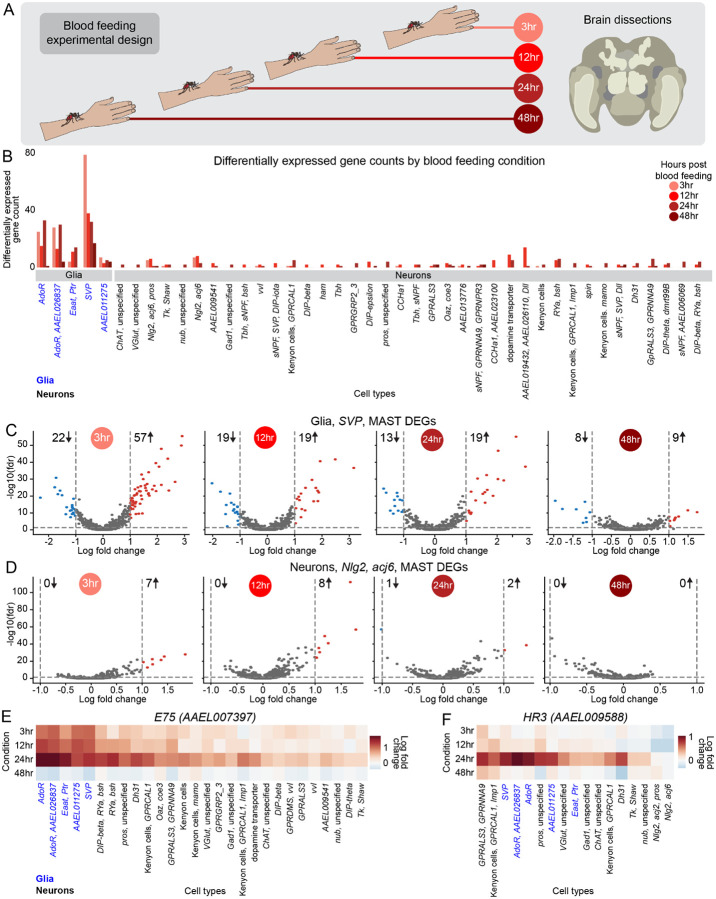
Glia show extensive transcriptional changes after blood feeding **(A)** Blood feeding experimental design. **(B)** Bar plot of number of differentially expressed genes between blood feeding conditions and sugar-fed female brain, per cell type. Bars colored by blood feeding condition. Glia and neuron cell types listed below bars. Genes thresholded on |log fold change| > 1 and false discovery rate (fdr) < 0.05 (determined by MAST on normalized expression, which is ln([(raw count/total cell counts)x median total counts across cells]+1)). **(C-D)** Volcano plots of differentially expressed genes across blood feeding conditions in the glial, “*SVP”* cell type (C) and the neuronal *, “Ngl2, acj6”* cell type (D). All significant genes (indicated in red) a |log fold change| > 1 and false discovery rate < 0.05, determined by MAST on normalized expression. Number of downregulated (blue) and upregulated (red) genes at each timepoint is indicated by the down/up arrows. **(E-F)** Heatmaps of log fold change of *E75 (AAEL007397)* (E) and *HR3 (AAEL009588)* (F) across blood feeding conditions compared to sugar-fed female brain by cell type. Cell types are sorted by the total log fold change across all timepoints and colored as glia (blue) or neurons (black). Cell types included have over 10 cells in each timepoint, and at least one timepoint where change from sugar-fed condition had a false discovery rate < 0.05.

The ingestion of blood sets off a sequence of dramatic physiological and behavioral events in the female mosquito. To understand these changes from the vantage point of neurobiology with single-cell resolution, we collected female brains at 3, 12, 24, and 48 hours after blood feeding (Figure 8A). For each timepoint post blood feeding, we calculated differentially expressed genes compared to sugar-fed females using MAST^54^ (Figure 8B, Table S10, and Zenodo Supplemental Data). We examined changes in gene expression for each cell type in the female brain across these timepoints. We did not observe any notable changes in cell-type abundance across cell types across the four timepoints after blood feeding (Figure S25A).

Contrary to our expectation that neurons would show a strong response to blood-feeding, glial cell types had dramatically more transcriptomic changes across blood-feeding timepoints than neurons (Figure 8B). For example, the glial cell type marked by *SVP* (*AAEL002765*) showed the most dramatic transcriptomic shifts with blood feeding compared to any cell type in the brain. *SVP* glia undergo rapid blood-feeding induced changes in gene expression, with the number of significantly differentially-expressed genes peaking at 3 hours post blood feeding, and progressively returning closer to the non blood-fed state at later timepoints (Figure 8B). There were 79 significantly differentially expressed genes at 3 hours, 38 at 12 hours, 32 at 24 hours, and 17 at 48 hours. Gene expression shifted from upregulation at 3 hours with 72% of differentially expressed genes upregulated to roughly equal numbers of upregulated and downregulated genes at the three other timepoints (Figure 8C, Table S10).

Neurons in general showed more muted responses to blood feeding, although 38 of 47 neuronal cell types with greater than 10 cells at each timepoint expressed at least two significantly differentially expressed genes. The neuronal cell types marked by “*Nlg2*, *acj6*, *pros*”, “*Ngl2*, *acj6*”, “*AAEL019432, AAEL026110, Dll”*, dopamine-related genes, “*RYa, bsh”* displayed the highest number of significantly differentially expressed gene(Figure 8B, Table S10). Nevertheless, the differential changes in glia cells were more dramatic than in neurons; for example, the neuron cell type marked by *Nlg2*, *acj6* had fewer differentially expressed genes than any of the glial cell types. The number of differentially expressed genes across timepoints was 7 at 3 hours, 8 at 12 hours, 3 at 24 hours, and no differentially expressed genes at 48 hours (Figure 8D and Table S10).

Next, we investigated the expression dynamics of individual genes at cell-type resolution across timepoints. *E75* (*AAEL007397*), *EcR* (*AAEL019431*), and *HR3* (*AAEL009588*) are nuclear steroid hormone receptors that play vital physiological roles in ecdysone signaling. Ecdysone signaling has many functions in insects, including for regulating female reproduction in mosquitoes after blood feeding^148^. All three of these genes are widely expressed in glia and neurons in the non blood-fed female brain (Figure S27A–S27D). Both *E75* and *EcR* show significant upregulation in all glial cell types and several neuronal cell types in at least one timepoint after blood feeding. Their expression is highly upregulated from 3 to 24 hours post blood feeding, which peaked at 24 hours and then to very low levels by 48 hours post blood feeding across all cell types (Figure 8E and Figure S27E). Cell-type resolution reveals that *E75* and *EcR* upregulation in the brain in the first 24 hours after blood feeding occurs in predominantly glia rather than neurons, suggesting that the post-blood feeding physiological role for *E75* and *EcR* is primarily glia-related (Figure 8E and Figure S27E). *HR3* expression was low across all cell types at 3 hours and 12 hours and then sharply increased at the 24-hour timepoint in both neuronal and glial cell types, suggesting a different mode of function than *E75* and *EcR* (Figure 8F).

Our analyses revealed additional examples of brain cell-type specific gene expression changes post blood feeding (Figure S27F–S27J). *IA*-*2* (*AAEL005692*) insulin-like peptide showed upregulation in a small subset of neurons at 12 hours and 24 hours post blood feeding and then was downregulated in many more subpopulations of neurons and glia at 48 hours (Figure S27F).

*fru* (*AAEL024283*) and *dsx* (*AAEL009114*) encode transcription factors that regulate sex-specific behaviors and sexual dimorphism in insects. Compared to sugar-fed expression levels, *dsx* was downregulated at 3 hours, 12 hours, and 24 hour timepoints almost exclusively in glia, and then returned to near-baseline levels by 48 hours (Figure S27G). Conversely, the transcription factor *fru* showed more modest changes in various cell types, although was significantly downregulated in *SVP* glia at 48 hours (Figure S27H). Cell type-specific *fru* regulation has also been observed in *Drosophila melanogaster*, where the male isoform of *fru* masculinizes brain circuitry through unique regulatory patterns of effector genes in different neuronal cell-types^149–152^. Whether the downregulation of *Aedes aegypti fru* predominantly in *SVP* glia during bloodfeeding could play a similar role in the behavioral states of the female mosquito is unknown.

Lastly, the clock genes *ITP* and PER showed upregulation specifically in glia (Figure S27I–S27J). Together these data point to potential regulators for gene expression changes both globally and in specific cell types across blood-feeding timepoints in the brain.

Our data confirm that gene expression changes in the female mosquito brain are correlated with blood-feeding state^27^. We found that while these changes occur in some neuronal cell types, the largest transcriptomic changes in the female brain post blood meal were in glial cell types. The functional implications of these glial transcriptomic patterns during blood feeding and how they might impact mosquito metabolism, physiology, and behavior require further study.

## Discussion

### A cell atlas of the adult male and female *Aedes aegypti* mosquito

We present the first comprehensive snRNA-seq cell atlas of adult male and female *Aedes aegypti*, which we expect will serve as a vital resource to the mosquito research community and scientists interested in comparative genomics. This analysis of 367,745 nuclei from 19 tissues provides insights into mosquito cellular diversity and function with a focus on sexual dimorphism across tissues. Using both unbiased approaches and orthology with *Drosophila melanogaster* genes, we identified specific cell-type gene markers and annotated individual tissues sampled from the entire mosquito. All data and annotations are available through the UCSC Cell Browser (http://mosquito.cells.ucsc.edu)^60^ to allow future exploration of the gene expression and cell types of this deadly disease vector. The Mosquito Cell Atlas will aid the identification of specialized cell types and their molecular signatures as potential targets for vector control, particularly in disrupting host-seeking behavior or pathogen transmission, and the investigation of sexually-dimorphic and blood feeding-related mosquito physiology and behavior. For example, in the testes we identified cell-type specific markers throughout spermatogenesis that may provide new targets for male sterility approaches for population suppression or be used for developing more effective gene drive strains^153,154^. In the salivary gland, we mapped 24 previously unlocalized secreted proteins via their cell-type specific distribution, which could inform transgenic expression of antiviral effector molecules. Our identification of specialized fat tissue cells expressing antimicrobial peptides provides an opportunity to investigate cells with direct influence on vector competence and mosquito immunity in fighting off viral infection. Beyond potential translational uses of the cell atlas, we anticipate that it will enable further development of molecular tools, including cell-type specific drivers. Finally, we anticipate that these data will be useful for cross-species comparisons^155^.

### Sexual dimorphic organization of receptors in the antenna

Our analysis revealed several unexpected examples of sexual dimorphism in the *Aedes aegypti* antenna. First, we found previously unknown male-specific *ppk317*-expressing epithelial cells in antennal joints. No counterpart to the *ppk317* cell type in females was identified in our data. We note that this could be due to this cell type not existing in the female, or a related cell type existing in the female but not expressing the *ppk317* gene. Among the *Aedes aegypti* PPK gene family, only *ppk301* has been functionally characterized^118^. However, studies in *Drosophila melanogaster* show that PPKs can have diverse functions. For instance, *ppk4* and *ppk11* are important for larval liquid clearance^116,156^, and *ppk23*, *ppk25*, and *ppk29* play a role in male courtship and pheromone detection^135,157–159^. Although the function of *Aedes aegypti ppk317* and these male antennal cells is unknown, the closest homologues in *Drosophila melanogaster* are the *ppk1, rpk*, and *ppk26* gene group^118^, which have been implicated in mechanical nociception in multi-dendritic neurons^117,160^. Future genetic and functional work is needed to understand the role of this male-specific PPK gene, and whether this novel cell type in males might be important for male antenna function or behavior.

Second, although there was unexpectedly little sexual dimorphism in chemosensory neurons in the antenna, we did identify a specific cell type marked by *Ir41l* where a single receptor, *Or82*, is absent in male cells and present in female cells. This sexual dimorphism was precise because *Or82* was expressed in both male and female cells marked by *Or3*. This cell-type specific transcriptional regulation suggests active mechanisms both controlling the expression of OR genes and downstream sexually dimorphic sensory processing. In turn, this could allow for the precise tuning of sensory responses across sexes, while maintaining overlapping olfactory responses and behaviors. Such organization might represent an efficient evolutionary solution for developing sexually dimorphic behaviors while preserving essential sensory functions common to both sexes. Investigating the transcriptional regulation of *Or82* could reveal mechanisms controlling sexual dimorphism in sensory systems. The ligand specificity of *Or82* is unknown and it will be interesting to learn if female-specific expression of *Or82* in *Ir41l* neurons is important for an aspect of female-specific sensory behavior.

### Widespread receptor co-expression in *Aedes aegypti* sensory appendages

Mosquito sensory neurons challenge canonical principles of chemosensory organization through extensive co-expression of receptors. Our data extend recent findings of co-receptor and ligand-specific receptor co-expression in antennal and maxillary palp neurons^43,44^ to other major sensory appendages such as the proboscis and tarsi, suggesting a fundamental organizational principle across mosquito sensory systems. We observe multiple patterns of receptor co-expression. First, neurons frequently co-express multiple ligand-specific receptors from the same family, as demonstrated by *Or82*, *Or3*, and *Or47* co-expression in single antennal cells. Second, we find many examples of cell-types that co-express receptors from multiple families (ORs, IRs, GRs, PPKs, TRPs) throughout sensory tissues. This is exemplified by *Or82* expression in *Ir41l* cells, which we validated through RNA *in situ* hybridization. These data suggest that there is coordinated receptor co-expression across gene families.

This complex organization may represent an evolutionary adaptation enabling efficient processing of environmental cues. While co-expression in antennae and maxillary palps has been hypothesized to enhance host detection^43^, its presence in proboscis and tarsi suggests a broader strategy. By co-expressing different receptor families, mosquito sensory neurons can process diverse chemical cues simultaneously to enable specificity of behavioral responses in different contexts. Alternatively, co-expression could enable redundant detection that enhances signal reliability. The polymodal nature of some sensory neuron cell types may be particularly advantageous for *Aedes aegypti* as a human specialist, allowing robust host detection despite variations in human odor profiles and continuous environmental changes. Critical questions remain about the molecular mechanisms underlying this organization: Does expression across receptor families lead to co-expression of functional receptor complexes? How do they interact? How is this information integrated by higher-order neurons? Understanding these mechanisms could reveal new approaches for vector control targeting multiple receptor systems simultaneously.

Beyond chemoreceptor gene distribution, we discovered coordinated and specific expression patterns of neuropeptide receptors across sensory neurons cell-types. Some receptors show broad expression, while others display restricted patterns correlating with the chemoreceptor expression profiles. This organization could enable modulation based on internal state, like host seeking, post-blood-feeding behavior, and oviposition. Future work should examine how neuropeptide signaling modifies sensory neuron function and whether specific receptor combinations enable flexible adjustment of sensory processing based on global physiological states.

### Sexual dimorphism and glial plasticity in the mosquito brain

Our analysis reveals new cell-types for the study of sexual dimorphism in the mosquito brain. Kenyon cells, associated with learning and memory^145^, in particular those marked with *GPRCAL1* and *Imp1*, show striking sex-related differential gene expression. This includes male-specific expression of neuropeptide Y receptor (*GPRNPY6*) and female-specific expression of protein kinase A receptor (*Pka*-*R1*), suggesting sex-specific neuromodulation of these circuits. Neuroanatomical evidence supports this sexual dimorphism, with some male Kenyon cells showing larger size despite overall smaller male brains^161^. Given that mushroom bodies process innate behaviors and internal states^162^, these sexually dimorphic Kenyon cells may contribute to sex-specific behaviors in mosquitoes such as host seeking or male courtship.

Glial cells emerge as key cell types in both sexual dimorphism and blood-feeding response. Recent comparative work in drosophilids (*Drosophila melanogaster*, *Drosophila simulans*, and *Drosophila sechellia*), showed that glia exhibit the highest expression divergence in the central brain^147^. In *Aedes aegypti*, glia also exhibit higher transcriptomic divergence between males and females than neurons. This points to a fundamental role of glia in the insect brain, where their transcriptional plasticity may provide a permissive substrate for evolutionary and sexually dimorphic plasticity, allowing for the emergence of novel properties without disrupting the more conserved functions of neuronal circuits.

We also show that glial cells, more than neurons, undergo extensive transcriptional changes following blood feeding. The significance of glia in sexual dimorphism and behavioral state transitions suggests a broader glial function than previously recognized^163,164^. Glia may function as master regulators of the mosquito brain, or acutely responsive to sex or state-related cues. Several factors may explain their extensive response.

First, as regulators of blood-brain barrier permeability, glia are ideally positioned to detect blood-derived signals and trigger an immune response if needed^165^. The perineurial glia of the blood-brain barrier demonstrate transcriptomic divergence between *Drosophila sechellia*, which feeds on the low-carbohydrate noni fruit, and *Drosophila melanogaster*, which feeds on yeast on rotting fruit, suggesting that glial transcriptional changes could be related to divergent requirements for sugar uptake in the brain^147^.

Second, glia can broadly influence neural circuit function by releasing neuroactive molecules and controlling the extracellular environment^166,167^.

Third, given the critical role of glia in neuronal metabolic support^168^ and the extensive metabolic demands of blood meal processing^29,169,170^, their transcriptional response may reflect adaptation to meet new metabolic needs.

Fourth, the temporal dynamics of glial gene expression, particularly in nuclear steroid hormone receptors like *HR3* and *E75*, suggest a transcriptional cascade that could maintain prolonged suppression of host-seeking behavior after blood feeding.

Understanding how specific glial populations influence neuronal function and behavior through these pathways could reveal novel aspects of glia-neuron interactions and their role in regulating mosquito behavior.

### Limitations of study and future directions

While our cell atlas provides many insights into mosquito cellular diversity, several limitations should be considered. Although snRNA-seq enables unified profiling of all mosquito tissues, nuclear transcriptomes may not fully reflect cytoplasmic mRNA levels and provide no insights into protein expression^171^. This is particularly relevant for chemoreceptor co-expression studies, where post-transcriptional regulation could affect final receptor composition^172^. In addition, other detected transcripts could be untranslated, as seen in the recent work looking at ORs in the clonal raider ant *Ooceraea biroi*^173^.

Annotation of the *Aedes aegypti* genome is imperfect. Overlapping gene annotations may lead to multimapping of transcripts during genome alignment and would cause transcripts to be discarded. *Or111* and *AAEL019786* are not visible in the processed data files for this reason. Therefore cautious interpretation of low or undetectable genes is important.

Our annotation relies heavily on *Drosophila melanogaster* orthology despite 260 million years of evolutionary separation^58,59^, potentially causing us to miss mosquito-specific adaptations. While we achieved high coverage across tissues with the profiling of 367,745 nuclei, some rare cell types may remain undetected, particularly those comprising few cells per tissue. Furthermore, although we characterized 19 mosquito tissues, most were not discussed in depth in this paper, leaving substantial data for future exploration by the mosquito biology community.

In the antenna, proboscis and tarsi, we observed extensive sensory receptor co-expression. Our validation of sensory gene expression was limited to a few specific cell types. Determining whether those receptors form functional complexes, and characterizing other multi-receptor cell types, will require electrophysiological, genetic, and behavioral studies. In addition, while we identified sexually dimorphic expression patterns in the antenna and brain, determining whether these differences are causally linked to behavioral dimorphism will require further study.

## Materials and methods

### Human and animal ethics statement

Blood feeding procedures and behavioral experiments with live hosts were approved and monitored by The Rockefeller University Institutional Animal Care and Use Committee (IACUC protocol 23040 (PRV 20068)) and Institutional Review Board (IRB protocol LV-0652), respectively. Human participants gave their written informed consent to participate in this study.

### Mosquito rearing and maintenance

*Aedes aegypti* wild-type (Liverpool) mosquitoes were reared in an environmental chamber maintained at 26°C ± 2°C with 70–80% humidity with a photoperiod of 14 h light: 10 h dark as previously described^121^. Embryos were hatched in 1 L hatching broth: one tablet of powdered Tetramin (TetraMin Tropical Tablets 16110M) in 1 L of deionized water, then autoclaved. Larvae were reared in deionized water (3 L total) and fed 3 crushed Tetramin tablets on the first day post hatching and 2 tablets daily thereafter. To maintain low rearing density, ~400 larvae were kept in 3 L deionized water from L3-L4 stage. Adult mosquitoes were supplied with unlimited access to 10% sucrose solution (w/v in deionized water), delivered in a glass bottle (Fisher Scientific FB02911944) with a cotton dental wick (Richmond Dental 201205), and were kept in 30 cm^3^ BugDorm-1 Insect Rearing Cages (BugDorm DP1000). Animals were dissected on day 7 of adulthood (14 days post hatching). All dissected animals were mated unless indicated as virgin animals. Virgin animals were sexed as pupae and isolated with their same-sex siblings prior to eclosion.

### Photographs of mosquito tissues

7–14 day-old mosquitoes were cold-anesthetized and kept on ice. The indicated tissues were freshly dissected using using Dumont #5 Forceps (Fine Science Tools 11295–10/11295–20 or Roboz Surgical RS-4955) ice in 1 X PBS (Thermo Fisher Scientific AM9625). Only brains were pre-fixed in 4% paraformaldehyde (Electron Microscopy Sciences 15710-S) in 1X PBS, 0.25% Triton X-100 prior to dissection for 3 h at 4°C. Tissues were placed on a stage micrometer (Fine Science Tools 29025–01) and photographed using an iPhone X (Apple) through the iDu Optics LabCam adapter (iDu Optics) attached to the eyepiece of a Nikon SMZ1500 stereo zoom microscope (Nikon). A scale bar of 500 μM was added to the images using the stage micrometers scale (Fine Science Tools 29025–01).

### Tissue collection

Adult wild-type (Liverpool) mosquitoes aged 7 days were aspirated using oral aspirator (John W. Hock Company 612) into a 16 ounce container (Webstaurant KH16A-J8000) and were sealed using double 0.8 mm polyester mosquito netting (ahh.biz F03A-PONO-MOSQ-M008-WT) then anesthetized on ice for 10 minutes. Mosquitoes were then placed in a 40 μm cell strainer (Falcon 352340) in a 100 mm Petri dish (Corning 430293) and soaked in ice-cold molecular-grade 100% ethanol for 5–10 seconds. The animals were rinsed in ice-cold Schneider’s Medium (Gibco 21720024) and placed in a clean Petri dish with ~20 mL ice-cold Schneider’s Medium on a reusable ice pack (GenTap, Cooler Shock. Amazon.com 854850006121). Tissues of interest were dissected using Dumont #5 Forceps (Fine Science Tools 11295–10/11295–20 or Roboz Surgical RS-4955) on a 100 mm Petri dish (Corning 430293) lined with or without SYLGARD 184 silicone (World Precision Instruments SYLG184). Tissues were placed directly into a DNA LoBind 1.5 mL tube (Eppendorf 022431021) pre-wet with 100 μL Schneider’s Medium on wet ice or in a 70 μm cell strainer (pluriSelect 43–10070–70) and DNA LoBind 1.5 mL tube (Eppendorf 022431021) pre-wetted with 100 μL Schneider’s Medium on ice by inverting the cell strainer over the Eppendorf tube using Dumont #5 Forceps (Fine Science Tools 11295–10/11295–20 or Roboz Surgical RS-4955) and pipetting 300 μL ice-cold Schneider’s Medium onto the strainer to expel the tissue. Each sample was collected in 90 minutes or less. The Eppendorf tube was wrapped in parafilm (Bemis Company Inc. PM996), flash-frozen in liquid nitrogen and stored at −70°C. All tissues were dissected in the Vosshall Laboratory at Rockefeller University. With the exception of two antenna samples, all samples were shipped to Baylor College of Medicine on dry ice for nuclei extraction. For individual sample information, see Table S1.

### Human blood feeding for blood-fed brain samples

Approximately 30 4–7 day old female mated adults were aspirated into a 30 cm^3^ BugDorm-1 Insect Rearing cage (BugDorm DP1000) and allowed to feed on a human arm for 20–30 minutes. One human subject was used for all blood feeding. Fed females were placed in an environmental chamber maintained at 26°C ± 2°C with 70–80% humidity with unlimited access to 10% sucrose solution until they reached 7 days of adulthood and were dissected. Brain dissections and collections were performed as described above.

### Single-nucleus suspension

Single-nucleus suspensions were prepared as described previously^174^. Thawed samples were spun down using the bench-top centrifuge, removing the Schneider’s medium as much as possible. Samples of like tissues were combined into one tube using a pipette with wide-bore tips and then centrifuged. Large tissues such as whole body/thorax/head were ground using a pestle motor (Kimble 6HAZ6) for 30 seconds on ice after thawing.

Samples were resuspended in 900 μL of fresh homogenization buffer (250 mM sucrose, 10 mM Tris PH 8.0, 25 mM KCl, 5 mM MgCl_2_, 0.1% Triton-x 100, 0.5% RNasin Plus, protease inhibitor, 0.1 mM DTT in 10 mL nuclease-free water) and transferred into a 1 mL Dounce (Wheaton 357538). Sample tubes were rinsed in 100 μL of homogenization buffer and transferred into the same dounce. Dounce sets were autoclaved at 200°C for more than 2 hours before each use.

Nuclei were released by 20 strokes of loose dounce pestle and 40 strokes of tight dounce pestle. 1000 μL of the samples were filtered into a 5 mL tube through 35 μM cell strainer cap (Corning 352235) and then filtered using Flowmi (40 μM; BelArt H136800040) into a 1.5 mL Eppendorf tube. After 10 minutes of centrifuging at 1000g at 4°C the pellet was resuspended using 500 μL of 1xPBS/0.5% BSA with RNase inhibitor (9.5 mL 1x PBS, 0.5 mL 10% BSA, 50 μl RNasin Plus). Samples were finally filtered using a 40 μm Flowmi into a new 5 mL FACS tube (Corning 352052) and kept on ice. 10 μL of the sample was moved into a new 5 mL FACS tube with 190 μL PBS as unstained control for FACS. The remaining single-nucleus suspension samples were stained with Hoechst-33342 (Invitrogen H3570) and checked using a cell counter slide (Fisher Scientific 22–600-100) to confirm individual nuclei.

### Fluorescence-activated cell sorting (FACS)

Nuclei were stained with Hoechst-33342 (Invitrogen H3570) on wet ice (1:1000; >5 min). Hoechst-positive nuclei were collected using the BD FACSAria III Cell Sorter (BD Biosciences). 80k–150k individual nuclei were collected into one 1.5 mL RNAse-free Eppendorf tube with 300–500 μL 1x PBS with 0.5% BSA as the receiving buffer (with RNase inhibitor). Next, nuclei were centrifuged for 10 min at 1000 g at 4°C, and resuspended using 30 μL of 1x PBS with 0.5% BSA (with RNase inhibitor). 2 μL of the nucleus suspension was used to calculate the concentration on a hemocytometer. 20k nuclei per sample were loaded on the 10x controller (10X Genomics) to recover ~10k cells after sequencing. For tissue with very limited nuclei, all Hoechst-positive nuclei from single-nucleus suspensions were collected, and the counting step was skipped to maximize the target nuclei number.

### Library preparation and sequencing

10x Genomics sequencing libraries were prepared following the standard protocol from 10x Genomics 3’ v3.1 dual index kit with the following settings. All PCR reactions were performed using C1000 Touch Thermal cycler with 96-Deep Well Reaction Module (BioRad 1851197). Cycle numbers were used as recommended in 10x protocol for cDNA amplification and sample index PCR. As per 10x protocol, 1:10 dilutions of amplified cDNA were evaluated using a Qubit fluorometer (Thermo Fisher). Final libraries were evaluated using TapeStation (Agilent). The final libraries were sent to Novogene Corporation Inc. (Sacramento, California , USA) for Illumina NovaSeq PE150 S4 lane sequencing with the dual index configuration Read 1 28 cycles, Index 1 (i7) 10 cycles, Index 2 (i5) 10 cycles and Read 2 91 cycles. The sequencing depth was about 80,000 reads per nucleus.

### Gene Annotation File

Gene annotations were prepared from VectorBase (www.vectorbase.org, Release 58, as of June 2022) using the *Aedes aegypti* LVP_AGWG AaegL5.3 Genome^52,57^. These were merged with the manual chemoreceptor annotation from^31^, then double checked and corrected for errors manually as well as using AGAT and GFF3 toolkit^175,176^ and then processed using gffread^177^. For quick identification in downstream analyses, the prefixes “MT-”, “RP-” and “RR-” were appended to all AAEL gene IDs for mitochondrial, ribosomal protein, and rRNA genes, respectively. Final annotation file was assembled using Cell Ranger package (version 7.1.0) *mkgtf*^178^ using the *Aedes aegypti* genome, including the mitochondrial chromosome, downloaded from NCBI^52,179^ NCBI RefSeq assembly: GCF_002204515.2^52,179^. Gene annotation file (including prefixes identifying MT, PR, and RR genes) is available in Zenodo Supplemental Data.

### Alignment and ambient RNA removal

FASTQ files were aligned using 10x Genomics Cell Ranger 7.1.0 (include-introns set to “true”)^178^. While the Cell Ranger performs alignment, PCR duplication correction and identification of empty droplets, the cells are susceptible to ambient RNA noise. A droplet containing a nucleus may also contain remnant floating RNA, which can occlude the nucleus’ expression. We therefore used the CellBender package^180^ for ambient RNA correction (epochs = 200, fpr = 0.01). We used the Cell Ranger cell count estimate as the number of expected cells and set the number of total droplets to the recommended default value (generally 30,000 droplets for typical samples). We selected the learning rate based on the smoothness of ELBO value along the epochs, as suggested by the developers. For most cases, we used the default learning rate and in cases where the ELBO value was “wobbly” we chose x0.1, x0.5 or x0.01 as suggested in the CellBender package^180^. A list of parameter values is provided in Table S1 and scripts used for Cell Ranger and CellBender are available in Zenodo Supplemental Data.

### Quality control and cell filtering

For all downstream analysis, we used the Scanpy package (referred to as sc from here on^53^, in Python^181,182^ in addition to standard Python libraries such as numpy, pandas, matplotlib, csv, os, datetime^183–185^. Most analysis was carried out in Jupyter notebooks^186^, and all scripts and additional data are available on Zenodo Supplemental Data.

#### Quality control metrics:

We began by evaluating basic quality control metrics using *calculate_qc_metrics* function in Scanpy in each sample. We evaluated the distribution of each metric such as the total counts in a cell, total number of genes expressed in a cell and the number of cells each gene is expressed in to filter for high quality cells and genes. We also evaluated Mitochondrial (MT), rRNA (RR), and ribosomal protein (RP) fractional expression distribution across cells. These metrics are associated with apoptotic cells or are typically uninformative^187^, hence understanding their contribution to the expression of each cell is important. To err on the conservative side, we began by removing only a few cells that were clearly noisy or outliers. Specific parameters and scripts for each sample are in Table S1 and Zenodo Supplemental Data.

We also performed basic filtering in the gene space. First, as a standard practice in the analysis of scRNA-seq data, we removed RP genes from downstream computation, as they are typically uninformative and are often confounders in biological signals^187^. Additionally, to reduce noise in the data, genes that were expressed in fewer than 12 cells were also removed, unless they were registered as possibly biologically meaningful after discussion with MCA co-authors. For this, we compiled a list of around 2,464 genes that were of interest based on the current literature (see Zenodo Supplemental Data).

#### Data Normalization:

After basic clean-up, each sample was median library size normalized followed by log-transformation, which was recently shown to perform just as well, if not better, than more sophisticated transformations^188^. We used *sc.pp.normalize_total* function in Scanpy and took the natural logarithm of the data with a pseudocount of 1 to preserve zeroes. We then computed the top 4000 highly variable genes (*sc.pp.highly_variable_genes*), followed by a principal component analysis (PCA, 30 components). We then computed k-nearest neighbors using *sc.pp.neighbors(n_neighbors*=*30, use_rep*=*‘X_pca’, metric*=*‘euclidean’)* function in Scanpy. UMAP, tSNE, Force Directed Layout (FDL) visualizations were used for visualization of data in 2D.

#### Doublet detection:

For doublet detection, we used the scrublet package^189^. Scrublet expects an estimate of doublets as an input, for which we used the formula *y* = *0.000759x + 0.052721* from the expected multiplet table provided by 10X Genomics, where *x* is the total number of cells in the dataframe. The predicted doublets were then analyzed together with other quality metrics for data clean-up as described below.

#### Cell-type informed data filtering:

Combining all the metrics discussed above, cell filtering was performed through identification of low quality clusters. A typical strategy to filter individual cells relies on individual metrics such as library size or doublet score, which can be manual and less generalizable. We instead sought to utilize the entire transcriptome to first group cells and filter out clusters of cells that cumulatively have low quality scores for the above described set of metrics: doublet score, mitochondrial gene fraction, ribosomal protein fraction, total counts, gene counts and cell-type specific gene expression. We removed clusters of cells that demonstrated low quality features (Table S1). To do this systematically, we first identified obvious outlier clusters, using which we defined a threshold that was uniformly applied to all clusters in each sample. For clustering we used the PhenoGraph^190^ package with the Leiden algorithm (resolution_parameter = 5 or 10, see Table S1) as implemented in the *sc.external* module. We chose such a high value of resolution_parameter, which results in a large number of clusters, to ensure that only highly specific noisy clusters were removed from downstream analysis. At minimum, clusters from all samples were removed that had a mitochondrial gene fraction of 5 or higher, and a doublet score of 0.3 or higher (Table S1). In many cases, these thresholds were adjusted based on their distribution to retain only high-quality cells for downstream analysis (see individual sample scripts in Zenodo Supplemental Data), because low-quality cells confound the characterization of real biological features in the data. Thus, we prioritized our analysis on high quality cells to enhance our understanding of these uncharacterized cell types with minimal exceptions (see testes data below),

Since there is limited prior knowledge on basic quality metrics for single-nuclei data from mosquitoes, we sought to biologically guide and complement our cell-filtering strategy using whatever limited information we have about cell-type markers in mosquitoes. For the purposes of a preliminary annotation to inform cell filtering strategy, we queried genes that appear often in most samples and utilized those to represent broad cell type categories. In particular, we used *AAEL024921* (*nSyb*) for neurons, *AAEL027131* (*repo*) for glia, *AAEL019468* (*Ppn*) for hemocytes, *AAEL001168* (*grh*) for epithelial-like cells, *AAEL002417* (*troponin T*) for muscle, *AAEL001194* (*FASN1*) for fat cells. We also included *AAEL019457* (*Lim1*), a commonly expressed transcription factor, that typically labels a discrete subset of cells. Cells that expressed these genes were typically not removed in filtering and used as reference for setting thresholds (described above) and identifying outlier clusters (Table S1).

To validate male and female samples, we also queried *AAEL022912* (*Nix*), which showed markedly differential expression in male and female samples, as expected^27,191^.

Samples were then each reprocessed, which included renormalizing the data, re-computing highly variable genes, PCA, and nearest neighbors. Same sets of parameters were used. For clustering, we used the Jaccard + Louvain algorithm implementation of PhenoGraph at resolution 1 for downstream annotation and analysis, unless indicated otherwise^190,192^. Preliminary annotation was performed on each sample as described in the next section. Only one round of filtering (or cluster removal) was performed for each sample. Preliminary annotation was performed on each sample individually.

#### Exception for testes sample:

We processed the testes sample both with and without CellBender. We observed that CellBender removed spermatids, which after meiosis slow transcription, and therefore have low transcript counts^62^. For this reason, to detect spermatids in our snRNA-seq data, we did not apply ambient RNA removal and did not discard clusters with features such as low UMI-count (Figure S13A–S13D). Spermatids were readily identifiable by their low transcript count and their expression of *S*-*Lap* (*AAEL000108*), *DBF4* (*AAEL008779*)^64^, and *Orco* (*AAEL005776*)^65^ (Figures 2B, S13E–S13G). To avoid potential batch effects (from lack of ambient RNA removal) in the integrated Mosquito Cell Atlas object (Figures 1C–1F and S2), we used the testes data that were processed with ambient RNA removal and thus lacks spermatids. Testes data processed without CellBender are available on UCSC Cell Browser (http://mosquito.cells.ucsc.edu) and with CellBender at Zenodo Supplemental Data.

### Sample merging

In cases where we had multiple samples for a tissue, we merged data, including male and female samples. In general, for a more robust annotation of cell types informed by a greater number of cells, and to enable comparison across sexes, we merged male and female samples. Our preliminary annotations (see Methods: Annotations and gene selection) showed in most cases a noticeable similarity in general cell types in male and female samples. We used *AnnData.concatenate* function^193^ and repeated the processing as described above. Genes expressed in fewer than 18 cells were removed unless they were present in a more comprehensive list of genes of interest (20,587 genes, see Zenodo Supplemental Data). We then renormalized the data, re-computed highly variable genes, principal components (PCs), nearest neighbors, and re-clustered as described above, unless indicated otherwise. See Table S2 for list of final objects. All objects available through either UCSC Cell Browser (https://mosquito.cells.ucsc.edu) or Zenodo Supplemental Data.

### Batch correction

In the case of the two merged ovary samples, we saw a noticeable batch effect of unknown origin (Figure S1G). We batch-corrected all genes using *batchelor.fastmnn*^194^. Quality control and filtering was done iteratively and informed by annotations on individual and merged samples. It was not necessary to batch correct any other samples for our other analyses.

### Annotations and gene selection

In non-model organisms, lack of knowledge of expected cell types, absence of extensive gene characterization, and few established cell markers, makes cell-type annotation challenging. Prior to analysis and annotation, we contacted an international group of mosquito experts to solicit hypotheses about putative cell types, as well as potential cell markers or genes of interest. *Aedes aegypti* genes came from sources including previous mosquito literature and previous bioinformatics analyses assessing putative function or gene families from the AaegL5 genome.

#### *Gene orthology to* Drosophila melanogaster:

In addition to information collected from the Mosquito Cell Atlas Consortium, we used information from homologues in *Drosophila melanogaster* that have been better-characterized. Orthologous genes were assessed using Ensembl Metazoa BioMart database (Ensembl Genomes release 56^55^, BLAST (nucleotide or protein)^56^, or Vectorbase^57^. We also used curated and computed cell marker genes from the Fly Cell Atlas^25^. It is important to note that *Aedes aegypti* and *Drosophila melanogaster* are separated by 260 million years since their last common ancestor^58^, with distinct behaviors, life cycles and physiology, so relying on *Drosophila melanogaster* homology to interpret *Aedes aegypti* genes can be problematic.

For instance, in a comparative genomic study of *Drosophila melanogaster* and several mosquito species of developmental genes, while many were well-conserved, key developmental genes in *Drosophila melanogaster* (as well as other insects) were not identified in mosquito genomes^58^. The fibroblast growth factor (FGF) signaling pathway involved in many biological processes including cell differentiation and migration, is conserved between flies and vertebrates, but was not identified in mosquito species. Additionally, cases were also observed of increased copy numbers of developmental genes in mosquitoes. How these individual copies differ from their homolog in *Drosophila melanogaster* is not known. While some genes and pathways are conserved, divergence in gene function and expression patterns is also expected, which can easily lead to misinterpretation and errors in analysis if one relies too heavily on *Drosophila melanogaster* to benchmark discoveries in *Aedes aegypti*.

#### Gene marker selection:

Genes were selected based on *sc.tl.rank_genes_groups* and MAST^53,54^. Top computed marker genes for each cluster were each assessed visually (UMAP) and by comparing average gene expression across all clusters in the data object. Genes were manually selected based on their ability to confer information of cell type, orthology to known *Drosophila melanogaster* marker genes, and their distinctiveness as a marker gene across cell types in all datasets. For instance, the transcription factor *Sox100B* (*AAEL009473*) was used as a marker and commonly observed in sensory tissues. Recent work identifying these cells in *Drosophila melanogaster* tarsi suggests that *Sox100B*-expressing cells may be important for neural lamella formation^133^.

#### Annotation using gene markers:

Annotations were performed using a combination of semi-automated and manual methods. Principal annotations were performed on each tissue (Figures 2–3, 7, S3–S12, S16 and S19–S20) and on the integrated data object of all sugar-fed cells (Figure 1). Preliminary annotations were performed on each sample individually before merging all samples of each tissue (Table S1). Data were clustered using Louvain or Leiden algorithms and clusters were assigned cell-type annotations. Clustering resolution was set based on cellular complexity of tissue and amount of prior information on tissue cell types (Table S2). Clusters were assessed for mean expression of identified gene markers using outputs from MAST, UMAPs, heatmaps, violin plots and bar plots (Zenodo Supplemental Data). Clusters were assigned a cell-type annotation based on expression of thresholded gene markers or combinations of gene markers (Table S4, for annotation script see Zenodo Supplemental Data). Gene markers for each cell type were also assigned a threshold through assessment of mean expression levels across clusters (Table S4).

#### Sensory neuron annotations:

*nompC*-negative sensory neuron populations in the antenna, maxillary palp, tarsi and proboscis were annotated separately in a similar pattern to tissues (Figures 5–6 and S22–S23). We used the same combination of semi-automated and manual methods as described above, however for these populations, we attributed extra significance to a list of putative sensory genes that might affect the stimulus response profile of a given cell type (Table S4). Clusters were computed with the Leiden algorithm, at high resolution due to sensory neuron complexity (Table S2). Clusters were assigned a cell type annotation. Cell types were named for chemoreceptors uniquely expressed in a cell type.

In the antenna, despite separating clusters at high resolution (Leiden, resolution 10), we found at least 6 examples of chemoreceptor genes co-expressed within a cluster but not within the same cells - indicating mutual exclusivity - and could not be separated via clustering algorithms. This suggests that these cells may belong to distinct, but transcriptomically similar olfactory sensory neuron cell types (for instance, *Ir41b* and *Ir41*e in Figures 5C–5D, S18B, and Zenodo Supplemental Data).

### *S*ensory neuron analysis

For the antennae, maxillary palps, and proboscis samples, we subsetted and filtered *nompC*-negative sensory neurons for further analysis. We identified the neuronal population based on the expression of *Syt1* (*AAEL000704*), *brp* (*AAEL018153*), *nSyb* (*AAEL024921*) and *CadN* (*AAEL000597*) (Figure 5A, S21A, S22A). We excluded mechanosensory neurons based on the expression of the *Drosophila melanogaster* orthologue of mechanosensory receptor *nompC* (*AAEL019818*) (Figure S17A–S17B). We removed clusters with a high doublet score (Figure S17C–S17D). Before reclustering, we additionally removed individual nuclei with a doublet score above 0.15 (Figure S17H). This ensured a conservative filtering of potential doublets given our interest in possible co-expression of receptor genes. For the antenna, we also filtered on neuronal gene fraction to ensure we were only looking at high quality neuronal nuclei (Figure S17E), although we note that this step removed *Gr20* cells from our analysis (cluster 83, Figure S16B and S17). For wing and abdominal tip neuron subsetting, all neurons were included for assessment of putative sensory gene expression (Zenodo Supplemental Data). As with other tissues, we removed individual nuclei with a doublet score above 0.15 (Figure S17H).

### Comparison of cell types across conditions and sexes

#### Cell abundance comparison:

For the sexual cell-type abundance difference, the frequencies of each cell type in each tissue for both sexes were determined by calculating the proportion of each cell type relative to the total number of cells in the tissue. The sexual abundance difference index for each cell type in each tissue was calculated using the following equation (Figure S3–S11, S16, S19–S20, scripts in Zenodo Supplemental Data):

$$\text{abundance}\;\text{index}=\frac{\text{Frequency}(\text{female})-\text{Frequency}(\text{male})}{\text{Frequency}(\text{female})+\text{Frequency}(\text{male})}$$

#### Sexual abundance difference index:

Where cell type was categorized based on abundance difference across sexes, cells were considered “Female biased” if abundance index > 0.3; “Neutral” if abundance index was −0.3 to 0.3, inclusive; “Male biased” if abundance index < −0.3. In bar plots, if there are biological replicates, the value for each replicate was shown as dots, and the standard error was calculated.

#### Differentially expressed genes:

MAST was used to calculate the differentially expressed genes for cell-type annotation, across sexes, and blood-feeding conditions for each cell type^54^. Log fold change is represented by MAST coefficients *(coef)*.

For counting significantly differentially expressed genes in Figures 7–8 and S24C, MAST output files were thresholded for absolute value of *coef* above 1, and a false discovery rate of 0.05. *coef* was calculated from normalized expression (natural log). We only analyzed cell types with at least 10 cells in all conditions. In some cases, MAST *coef* could not be calculated for some genes due to their normalized-log expression being zero or close to zero in at least one of the conditions (NaN genes). NaN genes were included in DEG counts (bar plots) if they were expressed in greater than 10% of genes in at least one condition and had normalized expression value greater than 1 (Tables S5, S6, S9, S10, and Zenodo Supplemental Data). Most NaN genes did not meet this criteria and were discarded. No NaN genes met this criteria for generation of volcano plots (Figure 6, 7 and S25). NaN genes were left grey for log fold change heatmaps (Figure 8 and S26).

For male versus female differential gene expression analysis across annotated cell types in Figure S3–S11, S16, S19–S20, genes were discarded prior to analysis if they were not expressed in at least 10% of cells in each sex within each cell type. DEG counts were determined by genes that were |*coef*/log(2)| > 1 and a false discovery rate < 0.05.

#### Volcano plots, log fold change heatmaps:

Volcano plots and log fold change heatmaps on differentially expressed genes were made using MAST differentially expressed genes. Log fold change is represented by MAST coefficients *(coef)*. Volcano plots were made with *seaborn.scatterplot* on −log_10_(false discovery rate)^195^. Log fold change heatmaps using *seaborn.heatmap* on individual genes were made by identifying all clusters where the gene had a calculated false discovery rate < 0.05 in at least one timepoint. Heatmaps were sorted by sum of *coef* values. Only clusters that had more than 10 cells in each timepoint were included.

### Data visualization

#### UMAPs, Gene fraction visualization:

UMAP coordinates were created using *scanpy tl.umap* function on the constructed nearest neighbors graph (described above). The *min_dist* parameter used are described in Table S2. We visualized UMAPs using *sc.pl.umap* function.

We quantify gene signature expression by computing gene fraction defined as: np.asarray(np.sum(adata.X[:, genelist_indices], axis = 1)/np.sum(adata.X, axis = 1)).squeeze() * 100) and visualized on UMAP. This is the mRNA content represented by the genes in the list for a given cell as a fraction of total mRNA of the cell.

#### Dotplots, heatmaps, violin plots, stacked bar plots, box plots:

Clusters for dotplots and heatmaps were organized using *sc.tl.dendrogram, sc.tl.heatmap* functions followed by *sc.pl.dendrogram* or *sc.pl.heatmap* functions on selected genes. Violin plots were made using *sc.pl.violin* or *sc.pl.stacked_violin*. Proportion stacked bar plots were made using *matplotlib ax.bar*. Full heatmaps of all putative sensory genes expressed in selected sensory neurons are available in Zenodo Supplemental Data, in addition to wing and abdominal tip datasets. Boxplots made with *seaborn.boxplot* and *seaborn.stripplot*.

#### Neuropeptide-related genes heatmaps:

Neuropetide-related genes were identified from literature and *Drosophila melanogaster* orthology, as previously described^55,196,197^. Genes were considered expressed by a cell type if they had a normalized expression value of at least 1 and were expressed by at least 20 percent of all cells in that cluster. Heatmaps were visualized using seaborn *sn.heatmap*^195^.

#### Diffusion component analysis, partition-based graph abstraction, quantification of distance between clusters:

To quantify the transcriptomic difference between male *ppk317* and other antenna cell types, we applied diffusion components analysis (Figure S15E–F) using *sc.tl.diffmap*, with 80 diffusion components using the nearest neighbors graph (described above). Diffusion components have been widely used in single-cell data analysis to approximate phenotypic distances between subpopulations of cells^198–202^. Since the top diffusion components explain the most variance in the data^200,201^, we calculated the top correlating gene for the diffusion components 1 and 2 (Zenodo Supplemental Data). *ppk317* (*AAEL000873*) was the highest scoring gene of diffusion component 1 (|correlation score| > 0.89) (Figure S15E, first panel). Neuronal markers including *Syt1 (AAEL000704)* and *nSyb* (*AAEL024921*) ranked highly for diffusion component 2 (for both a |correlation score| > 0.72) (Figure S15E, second panel). We then selected top components based on eigengap as has been done previously^200,201^. We observed that the first major gap in eigenvalues occurred between 18th and 19th eigenvalues, as such we chose top 18 eigenvectors for further analysis: Eigenvalues 1 through 18. Partition-based graph abstraction (*sc.tl.paga*) was then made through recalculating nearest neighbors using thus computed diffusion components (Figure S15F). For boxplot in Figure S15J, pairwise Euclidean distances were computed to approximate phenotypic distance based on diffusion embeddings using *sklearn.metrics.pairwise_distances*^203^ and plotted with *matplotlib.boxplot*.

#### Correlation matrix heatmap:

To evaluate pairwise correlation of gene expression between clusters in Figure S15G, we computed the Pearson correlation coefficient matrix (*numpy.corrcoef*) between normalized gene expression matrices for every pair of clusters. We computed correlation between every pair of cells for every pair of clusters and reported the mean correlation value as a heatmap. Diagonal values (cluster to itself) represent intra-cluster correlation values, which vary based on features such as cell number and gene heterogeneity.

#### Raw counts scatterplot:

To generate the scatter plot on antenna olfactory sensory neurons in Figure S18C, raw transcript counts (unique molecular identifiers) for a list of putative sensory genes (Table S4) were counted and plotted for each sample using *matplotlib.scatter*.

### Comparison of *Aedes aegypti* brain to *Drosophila melanogaster* head snRNA-seq data

For comparison of the mosquito cell atlas (MCA) to the fly cell atlas (FCA), we used SAMap (v1.0.15^144^). SAMap was used according to documentation. All versus all NCBI BLAST (v2.9.0^56^) was run using the SAMap script map_genes.sh on the annotated proteins from the VectorBase-58 version of LVP_AGWG genome and the “all translation” file from the FB2023_02 version of the FlyBase genome. Analyses were performed on the FCA head dataset^25^ and the MCA all brain dataset. These datasets were subsetted into neurons and glia and abundant cell clusters were subsampled using scanpy. The FCA head dataset was subsetted using the FCA cell type annotation clusters. Clusters with mean expression of the gene *Dm_repo* >0.4 were considered glia and mean expression of *Dm_nSyb* >1.2 were considered neurons. Then cell clusters of neurons (Leiden algorithm, resolution = 4) with >1000 cells were subsampled down to 1000 using *scanpy.subsample*. The MCA brain dataset was subsetted using the Leiden algorithm (resolution = 5) clusters. Clusters with mean expression of the gene *repo (AAEL027131)* >2.0 were considered glia and mean expression of *nSyb (AAEL024921)* >0.7 were considered neurons. Neurons clusters with >1000 cells were subsampled down to 1000 using the subsample function in Scanpy. Subsampled neurons and all glia were then run in SAMap using default parameters. FCA and MCA neurons were run together, FCA and MCA glia were run together, and as a control FCA glia and MCA neurons were run together (Figure S26E–S26K). Mapping scores were determined between FCA cell type annotations and MCA (Leiden, resolution = 5) clusters. Kenyon cells (KCs) were identified in the MCA dataset by high mapping scores with the FCA KCs and expression of the known markers including *Hr51 (AAEL020847)* and *sNPF (AAEL019691)* (Figure S26L–S29M). We also queried markers for potential Kenyon cell subtypes in the MCA using *Pde8* (*AAEL019528*) (alpha/beta KCs), *mamo* (*AAEL019481*) (alpha’/beta’ KCs) and *Imp1* (*AAEL006876*) (gamma KCs) (Figure S26N–S29P)^25,146^.

#### Testes whole mount RNA *in situ* hybridization and imaging

Hybridization chain reaction RNA fluorescence *in situ* hybridization (RNA *in situ* hybridization) was conducted in whole male testes to detect RNA, using an adaptation of published protocols^204,205^. 1–3 days old adult male *Aedes aegypti* wild-type (Liverpool), were anesthetized at 4°C for 10 minutes. Testes were dissected from male mosquitoes in ice-cold PBST (1X PBS, 0.1% Tween-20) with 0.5% formaldehyde using Dumont biology tweezers (Agar Scientific T5291). The terminal abdomen was removed by grasping the upper abdomen and genitalia with separate pairs of forceps. Testes and male genital tract were cleaned of excess fat tissue. Dissected testes were fixed in 4% paraformaldehyde (made from 40% stock: 0.368 g paraformaldehyde, 1 mL RNase-free water, 7 μL 2N KOH, heated until dissolved and filtered through 0.3 μm filter) in PBST for 30 minutes at room temperature. Samples were washed twice in PBST for 5–10 minutes each, then dehydrated in 100% methanol and stored at −20°C in 100% methanol for up to 2 weeks. Prior to hybridization, samples were rehydrated by rinsing once in 70% ethanol and stored overnight at 4°C in 70% ethanol. The next day, samples were transferred to 0.2 mL PCR tubes (Azenta Life Sciences PCR1174) and rinsed twice with PBST. Samples were then pre-hybridized in 30% probe hybridization buffer (30% formamide, 5X SSC, 0.1% Tween 20, 50 μg/mL heparin, 5X Denhardt’s solution, and 10% dextran sulfate) at 37°C for 30 minutes. Probe solution was prepared by adding 0.4 μL of 100 μM probe stock to 100 μL hybridization buffer (Full list of probe sequences can be found in Table S11). Both samples and probe solution were heated to 80°C for 5 minutes before combining. Hybridization was performed overnight at 37°C in dry bath. Following hybridization, samples were washed four times for 20 minutes each in pre-warmed probe wash buffer (30% formamide, 5X SSC, 0.1% Tween 20, and 50 μg/mL heparin) at 37°C. Hairpin amplification was performed by heating 2 μL of each hairpin to 95°C for 90 seconds, cooling to room temperature for 30 minutes, then adding to 50 μL amplification buffer (5X SSC, 0.1% Tween 20, and 10% dextran sulfate). Samples were incubated in amplification buffer for 30 minutes at room temperature before overnight incubation with hairpin solution at room temperature in the dark. Samples were washed 5 times with 5X SSCT (5X SSC and 0.1% Tween 20) for 5 minutes each, followed by three 5-minute washes in 1X PBS. Tissues were then mounted in mounting medium on a cover slip and imaged. Images were acquired using an Olympus BX63 microscope (Olympus) equipped with a Cool LED pE-300 light source and Hamamatsu ORCA Spark camera (Hamamatsu Photonics C11440–36U), using 20x/0.80 UPlan XApo objective (Figures 2C and 2F–2G) or Olympus Uplan Fl 40x/0.75 objective (Figure 2D). Images were acquired as a 1920×1200 size image. Image acquisition was performed using Olympus cellSens software.

#### Antennae whole mount RNA *in situ* hybridization

RNA *in situ* hybridization was conducted in whole mount female and male antenna to detect RNA using adaptations of published protocols^43,204,206^. Products including HCR custom probes, amplifiers, probe hybridization buffer, probe wash buffer, and amplification buffer were purchased from Molecular Instruments Inc. (https://www.moleclarinstruments.com). All staining steps were done in a modified cell strainer snap cap (Fisher Scientific, Falcon 352235) in a well of a 24-well plate (Fisher Scientific, Falcon 353047).14-day-old adult Liverpool mosquitoes were anesthetized on wet ice. Antennae were dissected in a bubble of ice-cold 1X PBS (Thermo Fisher Scientific AM9625) in a 100 mm Petri dish (Corning 430293) lined with SYLGARD 184 silicone (World Precision Instruments SYLG184) on a reusable ice pack (GenTap, Cooler Shock. Amazon.com 854850006121) using Dumont #5 Forceps (Fine Science Tools 11295–10/11295–20 or Roboz Surgical RS-4955). Samples were digested in a chitinase-chymotrypsin solution [119 mM NaCl, 48 mM KCl, 2 mM CaCl_2_, 2 mM MgCl_2_, 25 mM HEPES, 5 U/mL chitinase (Sigma-Aldrich C6137–50UN), 100 U/mL alpha-chymotrypsin (Sigma-Aldrich CHY5S-10VL), 1% DMSO] rotating at 37°C for 1.5 hours. Antennae were washed in 1% PBS, 0.1% Tween-20 (PBST) for 10 minutes three times at room temperature. Samples were then fixed in 4% paraformaldehyde (Electron Microscopy Sciences 15710-S) in 1X PBS, 0.025% Triton X-100 for two hours at room temperature, following six five-minute washes at room temperature in PBST. Antennae were then dehydrated at 4°C in a stepwise sequence of 25% methanol/PBST, 50% methanol/PBST, 75% methanol/PBST, then 100% methanol twice, for 10 minutes at each step. Samples were kept in 100% methanol overnight at −20°C. The following day tissues were rehydrated at 4°C in a stepwise sequence of 75% methanol/PBST, 50% methanol/PBST, 25% methanol/PBST for 10 minutes each. At room temperature, samples were washed in PBST four times for ten minutes, fixed in 4% paraformaldehyde in PBS with 0.1% Tween for 20 minutes, and then washed again in PBST three times for 15 minutes. Antennae were transferred to preheated probe hybridization buffer at 37°C for 30 minutes. 8 μL of 1 μM stock of each probe was added to 800 μL of preheated probe hybridization buffer at 37°C, samples were transferred to this probe solution for two nights and kept at 37°C (Full list of probes can be found in Table S11). They were then washed four times for 15 minutes at 37°C in probe wash buffer, followed by four 15-minute washes in 5X SSC (Invitrogen 15557044) in nuclease-free water, 0.1% Tween 20 solution (SSCT) at room temperature. Antennae were then incubated in amplification buffer for 30 minutes at room temperature. Hairpin amplifiers were combined and activated per the manufacturer’s instructions. 8 μL of 3 μM stock hairpins were added to 800 μL of amplification buffer at room temperature overnight in the dark. At room temperature, samples were washed in SSCT twice for 15 minutes, incubated in 1:500 DAPI (Sigma-Aldrich D9542–5MG) in SSCT for one hour, then washed again in SSCT five times for 15 minutes. Tissues were then mounted on slides in SlowFade Diamond (Thermo Fisher S36972), topped with a coverslip, sealed with clear nail polish, and stored at 4°C until imaged.

#### Antennal confocal imaging and image processing

Confocal images of antennae were acquired on a Zeiss Axio Observer 7 Inverted LSM 980 scanning confocal microscope (Zeiss) with a 63x/1.40 PlanApochromat Oil DIC M27 objective. The sample was scanned bidirectionally without averaging (Figures 5E–F and S15K) or with 4x averaging (Figure 4F–K). The images were acquired as a standard 1024×1024 size image, which, depending on the zoom used, resulted in a voxel size of 0.0658 μm × 0.0658 μm × 0.24 μm (for Figure 4F–K) or 0.1315 μm × 0.1315 μm × 0.26 μm (for Figure 5E–F and S16A). Zen Blue v3.5 software was used for image acquisition.

For all comparative experiments, image acquisition parameters were kept consistent. We note that all confocal imaging was conducted in a manner that would maximize our ability to visualize the presence or absence of each fluorophore and was not intended as a quantitative measure of fluorescence intensity. Confocal images were processed in ImageJ (NIH). Brightness/contrast was adjusted to maximize visualization, and for all comparative experiments, adjusted parameters were kept consistent.

## Supplementary Material

Supplement 1

Supplement 2

## Data Availability

Supplementary Figures S1–S27 and Table S1–S11 accompany the paper. Processed data are available for user-friendly visualization, querying and download through UCSC Cell Browser (https://mosquito.cells.ucsc.edu). Raw snRNA-seq data have been deposited and can be downloaded from NCBI (BioProject: PRJNA1223381). Raw snRNA-seq data from female antenna and maxillary palp samples previously published in Herre, Goldman *et al*.^43^ and re-analyzed in this study can be downloaded from NCBI (BioProject: PRJNA794050). Additional raw and processed data, plots, analysis and custom scripts are available at Zenodo Supplemental Data (https://doi.org/10.5281/zenodo.14890013).
