# Supplementary material for: A single-nucleus transcriptomic atlas of the adult Aedes aegypti mosquito
Source: bioRxiv. 2025 Sep 30:2025.02.25.639765. Originally published 2025 Feb 25. Preprint. [Version 3] doi: 10.1101/2025.02.25.639765 (PMC11888250; doi:10.1101/2025.02.25.639765)
Supplement: Supplement 3 [file media-3.pdf]

Goldman *et al.* 2025, "A single-nucleus transcriptomic atlas of the adult Aedes aegypti mosquito"

Data S3
RNA *in situ* hybridization controls and probe information, related to Figures 2-4.

A

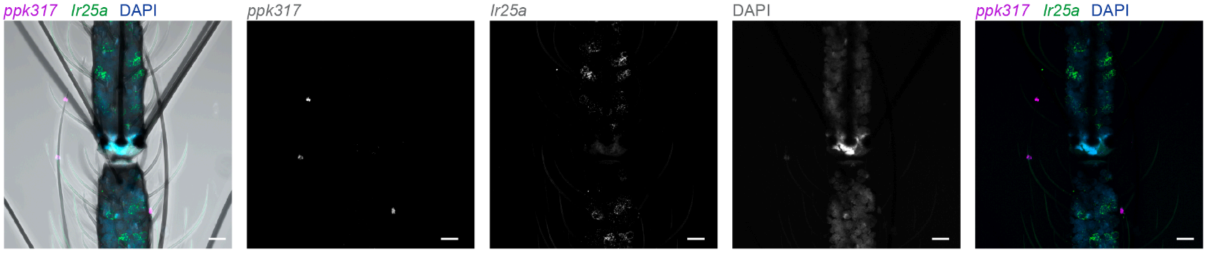

B

Target Name	Gene	Organism	HCR Amplifier	Molecular Instruments Probe Lot Number
ppk317	*AAEL000873*	*Aedes aegypti*	B2	RTR390
Ir41l	*Ir41l*	*Aedes aegypti*	B2	RTR716
Or82	*Or82*	*Aedes aegypti*	B4	RTR717
Or47	*Or47*	*Aedes aegypti*	B5	RTR718
Or3	*Or3*	*Aedes aegypti*	B2	RTR719
Ir25a	*Ir25a*	*Aedes aegypti*	B3	PRB511

C

Target Name	Gene	Organism	HCR Amplifier	Individual probe sequence
eya	*AAEL019952*	*Aedes aegypti*	B1	GAGGAGGGCAGCAAACGGAACCTGGGCTACGGATTTCCGATTTGA
				TCCCCCTGAGATGGCTGCGTCAAGTTAGAAGAGTCTTCCTTTACG
				GAGGAGGGCAGCAAACGGAACACCACTCATTCGTTTCTCTACCTG
				CAACTGCGTCGCTTCGTTCTTCTTTTAGAAGAGTCTTCCTTTACG
				GAGGAGGGCAGCAAACGGAATTGAAGAAGAAGAACGCATCCGCCA
				AACCTGATCGCACTCCTCCAGATCGTAGAAGAGTCTTCCTTTACG
				GAGGAGGGCAGCAAACGGAACTACTCGGTGGCAAGCAGCTGTCCG
				AGCCGTTGGATAATGCAGCGTTGAGTAGAAGAGTCTTCCTTTACG
AAEL001918	*AAEL001918*	*Aedes aegypti*	B4	CCTCAACCTACCTCCAACAATAGTTGTTGAAGCCGATGGTTCCAG
				GACATGTCAGTTGATGGCAGATCAGATTCTCACCATATTCGCTTC
				CCTCAACCTACCTCCAACAAATTGCTGTTGGCTGTCTGCTGATGT
				CTTGCGAAGGGAAACGAATCCTAGAATTCTCACCATATTCGCTTC
				CCTCAACCTACCTCCAACAATATGCCAACATCCCACGTCAAATGG
				CGGATTTTTTCTTCGCTTTGCCTCCATTCTCACCATATTCGCTTC
				CCTCAACCTACCTCCAACAACAGGCTGTTCCACTGGCAAATCTAT
				GTTTCTTCCGGAGCAAGCTGTTGAAATTCTCACCATATTCGCTTC
ana	*AAEL007208*	*Aedes aegypti*	B2	CCTCGTAAATCCTCATCAAACACCCCGCACAGCGCCATAGTTCCA
				ATGGCCACATCTGAGGCGTACGGGGAAATCATCCAGTAAACCGCC
				CCTCGTAAATCCTCATCAAAGCGCGTTGCATGTTCTTCTGGGCGG
				TTGCTTGATGGCCATCGGAGCCCCGAAATCATCCAGTAAACCGCC
				CCTCGTAAATCCTCATCAAATGCCGAATTAACCGAAGTGTAAGGC
				CGCCGATCATGTACAACCTCAACATAAATCATCCAGTAAACCGCC
				CCTCGTAAATCCTCATCAAAAACACGAATCATCTGCTCGGCGGGG
				TCTTGCGCCAGTACACGGAAACGGTAAATCATCCAGTAAACCGCC
beta2-tubulin	*AAEL019894*	*Aedes aegypti*	B2	CCTCGTAAATCCTCATCAAACCTCGGTGGTGAAAATCGAAGTGAA
				AATGGTTCCAAATCACGTCCGCGTTAAATCATCCAGTAAACCGCC
				CCTCGTAAATCCTCATCAAAGGATCCATTCGACGAAGTAGCTACT
				CAGACGGCCGTCTTCACATTGTTCGAAATCATCCAGTAAACCGCC
				CCTCGTAAATCCTCATCAAATGATCCGCTCCAGCTGCAAATCGCT
				CCGGTAGCCTCGTTGTAGTACACGTAAATCATCCAGTAAACCGCC
				CCTCGTAAATCCTCATCAAAGGGAATGGAACCATATTGACTGCCA
				GCCGGTCATGAAAAAGTGCAGCCGCAAATCATCCAGTAAACCGCC
Vas	*AAEL004978*	*Aedes aegypti*	B4	CCTCAACCTACCTCCAACAAGCTTCGTTGAAAATCTGTAGCGCCA
				AACGGTTCCAAGCGCAAACTTGCGGATTCTCACCATATTCGCTTC
				CCTCAACCTACCTCCAACAAAAATCGCTACGGCCGCCTCCTCGTC
				TTCTCCATCATTGTCGCCACCGCCGATTCTCACCATATTCGCTTC
				CCTCAACCTACCTCCAACAATTCGTTGTCGCCTCCGCCGAAGTCG
				CACCACGACCGAATCCGTTTTCTCCATTCTCACCATATTCGCTTC
				CCTCAACCTACCTCCAACAAGGGTTCTCTCCCGTTACGTTTACTT
				GCCGAACGACTTGATTGGGCTAGGAATTCTCACCATATTCGCTTC

Data S3. RNA *in situ* hybridization controls and probe information, related to Figures 2-4.

(A) Maximum-intensity projection of whole-mount female antennae with RNA *in situ* hybridization of *ppk317* probe (magenta), *Ir25a* (green) and nuclear staining (DAPI). Scale bar: 10 μm, related to Figure 3.

(B) Antennae RNA *in situ* hybridization probe information. Table includes targeted gene names, gene IDs, organism, HCR Amplifier, and probe lot numbers for ordering, related to Figures 3 and 4. To order probe sets, contact Molecular Instruments (https://www.molecularinstruments.com/) and provide the probe lot number.

(C) Testis RNA *in situ* hybridization probe information. Table includes targeted gene names, gene IDs, organism, HCR Amplifier, and probe sequences, related to Figure 2.
