# Supplementary material for: A single-nucleus transcriptomic atlas of the adult Aedes aegypti mosquito
Source: bioRxiv. 2025 Sep 30:2025.02.25.639765. Originally published 2025 Feb 25. Preprint. [Version 3] doi: 10.1101/2025.02.25.639765 (PMC11888250; doi:10.1101/2025.02.25.639765)
Supplement: Supplement 4 [file media-4.pdf]

Goldman *et al.* 2025, "A single-nucleus transcriptomic atlas of the adult Aedes aegypti mosquito"

Data S4

Filtering and characterization of *nompC (AAEL019818)*-negative sensory neurons, related to Figure 5.

Index:

(Data S4.1) Sensory neuron filtering and analysis, related to Figures 5 and S7.

(Data S4.2) Neuropeptide receptor and synthesis gene analysis in antenna, and characterization and sexual dimorphism in *nompC* (*AAEL019818*)-negative sensory neurons, related to Figures 4, 5, S6, and S7.

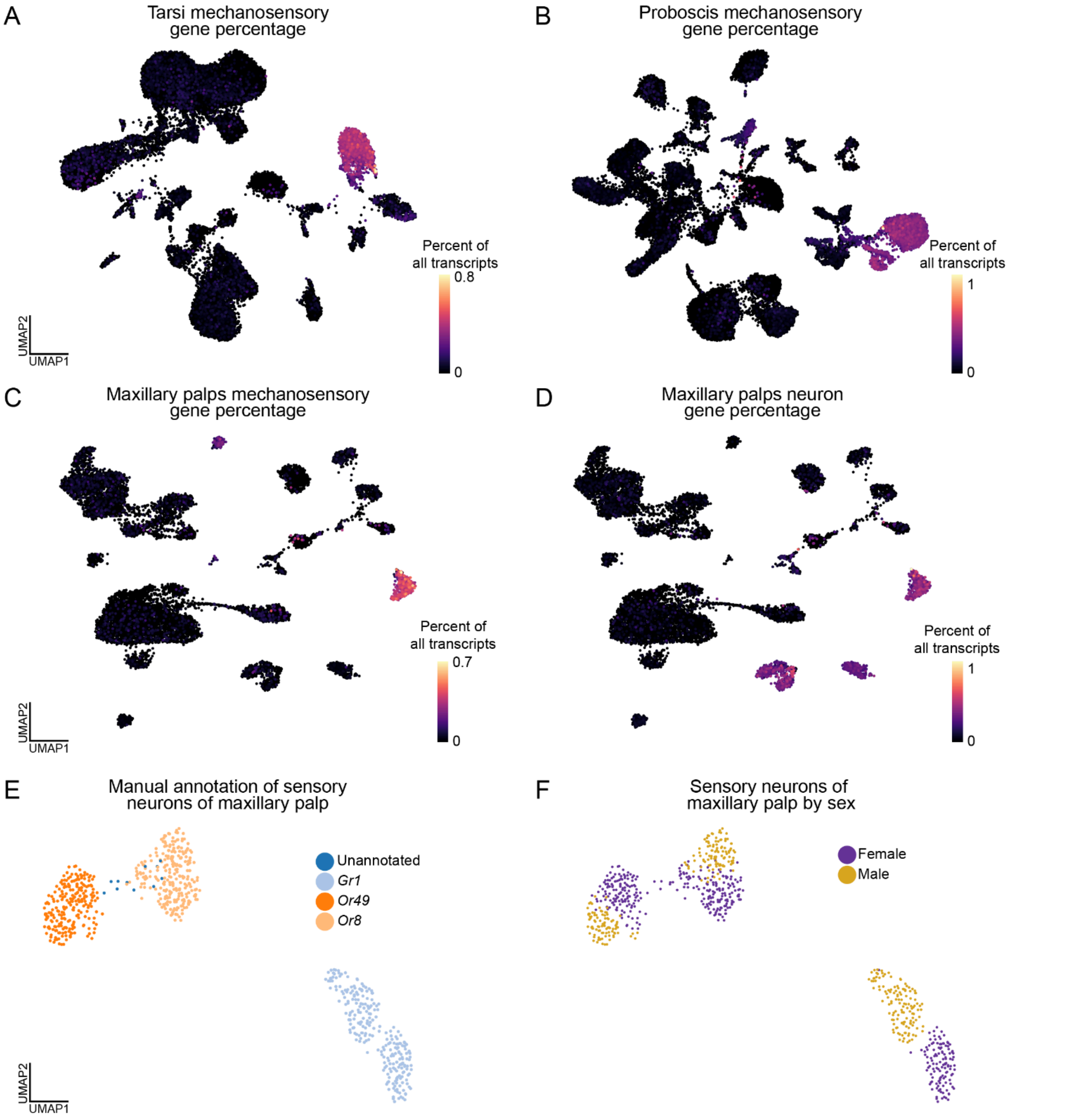

Data S4.1. Sensory neuron filtering and analysis, related to Figures 4 and 5.
(A-C) UMAP of neurons from tarsi (A), proboscis (B), and maxillary palps (C) data with expression of *nompC* (*AAEL019818*) as percent of all transcripts in each cell.
(D) UMAP of all maxillary palp cells with expression of neuron marker genes (*Syt1 (AAEL000704), nSyb (AAEL024921), brp (AAEL018153), CadN (AAEL000597)*) as percent of all transcripts in each cell.
(E-F) UMAP of *nompC*-negative sensory neurons from maxillary palps, colored by manual annotation (E) and sample (F) (female = 1, male = 1). Female sample previously published[36].

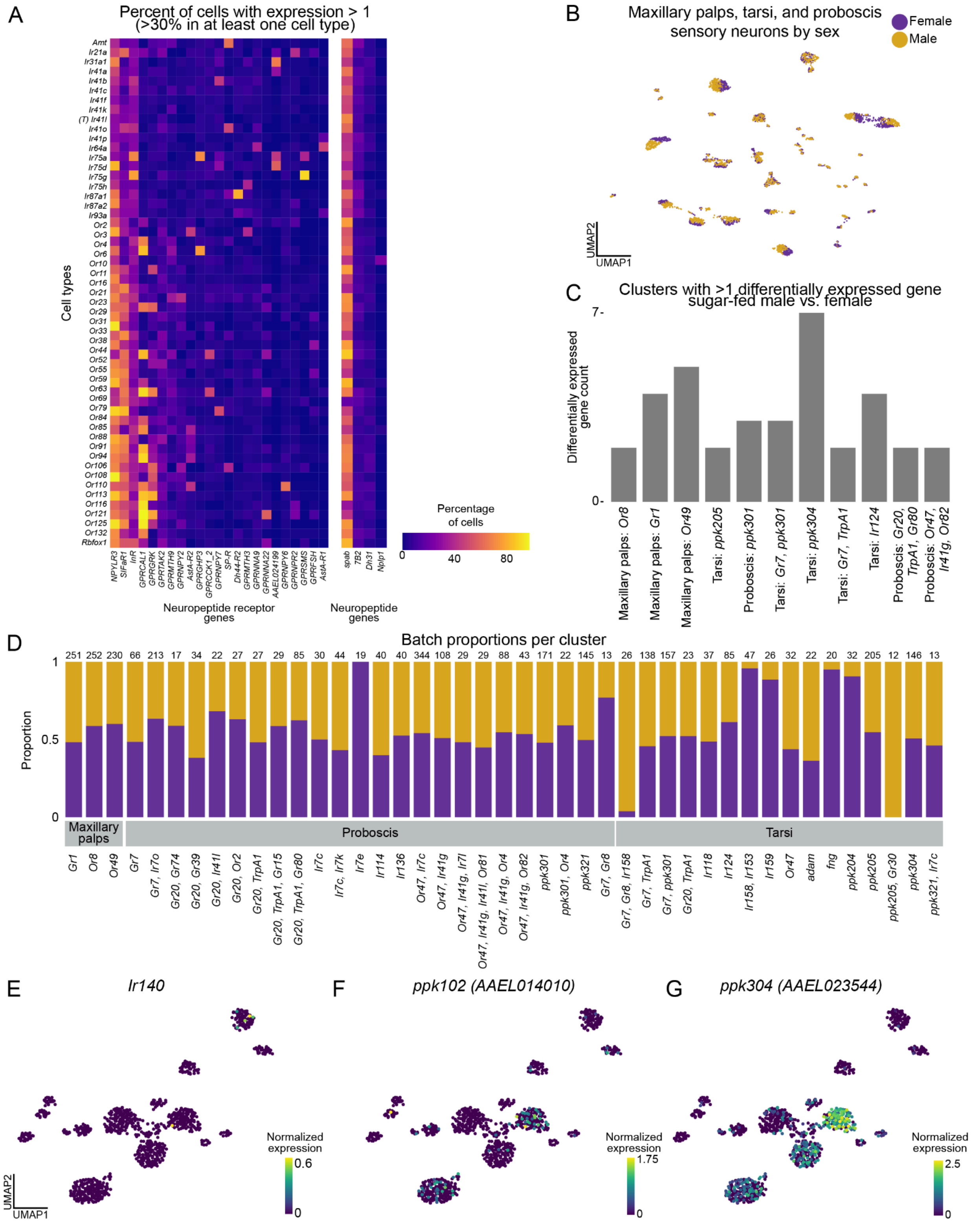
A
Percent of cells with expression > 1
(>30% in at least one cell type)
Cell types
Amt
Ir21a
Ir31a1
Ir41a
Ir41b
Ir41c
Ir41f
Ir41k
(T) Ir41l
Ir41o
Ir41p
Ir64a
Ir75a
Ir75d
Ir75g
Ir75h
Ir87a1
Ir87a2
Ir93a
Or2
Or3
Or4
Or6
Or10
Or11
Or16
Or21
Or23
Or29
Or31
Or33
Or38
Or44
Or52
Or55
Or59
Or63
Or69
Or79
Or84
Or85
Or88
Or91
Or94
Or106
Or108
Or110
Or113
Or116
Or121
Or125
Or132
Rbfox1
NPYLR3
SIFaR1
InR
GPRCAL1
GPRGRK
GPRTAK2
GPRMTH9
GPRNPY2
AstA-R2
GPRGHP3
GPRCCK1_2
GPRNPY7
SP-R
Dh44-R2
GPRMTH3
GPRNNA9
GPRNNA22
AAEL024199
GPRNPY6
GPRNPR2
GPRSMS
GPRFSH
AstA-R1
spab
TB2
Dh31
Nplp1
Neuropeptide receptor genes
Neuropeptide genes
Percentage of cells
0
40
80
B
Maxillary palps, tarsi, and proboscis sensory neurons by sex
Female
Male
UMAP2
UMAP1
C
Clusters with >1 differentially expressed gene sugar-fed male vs. female
Differentially expressed gene count
7-
0-
Maxillary palps: Or8
Maxillary palps: Gr1
Maxillary palps: Or49
Tarsi: ppk205
Proboscis: ppk301
Tarsi: Gr7, ppk301
Tarsi: ppk304
Tarsi: Gr7, TrpA1
Tarsi: Ir124
Proboscis: Gr20, TrpA1, Gr80
Proboscis: Or47, Ir41g, Or82
D
Batch proportions per cluster
Proportion
1
0.5
0
251 252 230 66 213 17 34 22 27 27 29 85 30 44 19 40 40 344 108 29 29 88 43 171 22 145 13 26 138 157 23 37 85 47 26 32 22 20 32 205 12 146 13
Maxillary palps
Proboscis
Tarsi
Gr1
Or8
Or49
Gr7
Gr7, Ir7o
Gr20, Gr74
Gr20, Gr39
Gr20, Ir41l
Gr20, Or2
Gr20, TrpA1
Gr20, TrpA1, Gr15
Gr20, TrpA1, Gr80
Ir7c
Ir7c, Ir7k
Ir7e
Ir114
Ir136
Or47, Ir7c
Or47, Ir41g
Or47, Ir41g, Ir7l
Or47, Ir41g, Ir41l, Or81
Or47, Ir41g, Or4
Or47, Ir41g, Or82
ppk301
ppk301, Or4
ppk321
Gr7, Gr8
Gr7, Gr8, Ir158
Gr7, TrpA1
Gr7, ppk301
Gr20, TrpA1
Ir118
Ir124
Ir158, Ir153
Ir159
Or47
adam
fng
ppk204
ppk205
ppk205, Gr30
ppk304
ppk321, Ir7c
E
Ir140
Normalized expression
0.6
0
UMAP2
UMAP1
F
ppk102 (AAEL014010)
Normalized expression
1.75
0
G
ppk304 (AAEL023544)
Normalized expression
2.5
0

Data S4.2. Neuropeptide receptor and synthesis gene analysis in antenna, and characterization and sexual dimorphism in nompC-negative sensory neurons, related to Figures 4, 5, S6, and S7.

(A) Heatmap of expression of neuropeptide receptor genes (left) and neuropeptide synthesis genes (right) within manually annotated olfactory sensory neurons (*nompC*-negative) in the antenna. Color scale indicates percentage of cells expressing a gene above threshold (normalized expression value of 1). Sensory genes are indicated in columns and cell types indicated in rows. Genes were included if they were expressed above threshold in over 20% of cells in at least one cell type. Genes filtered from lists in Table S1. Normalized expression is *ln([(raw count/total cell counts)*median total counts across cells]+1)*.

(B) UMAP of combined *nompC*-negative sensory neurons from maxillary palps, tarsi and proboscis samples, colored by sex.

(C) Bar plot of number of differentially expressed genes (DEGs) between females and males in each olfactory sensory neuron cell type. Clusters shown contain at least 1 DEG with a |log fold change| >1 and false discovery rate 0.05 (determined by MAST on normalized expression). For more information on DEGs, see Table S2.

(D) Proportion plot for annotated *nompC*-negative sensory neurons from maxillary palps, tarsi and proboscis. Bar plot of proportion cells from each sex that makes up each cell type. Numbers above each bar indicate the number of cells in that cluster. Cell types that are more than 70% from a single sex indicated in red.

(E-G) In tarsi *nompC*-negative sensory neurons, UMAP of normalized gene expression of *Ir140* (E), *ppk102 (AAEL014010)* (F), and *ppk304 (AAEL023544)* (G). Each end of color bar trimmed 0.1% for visibility.
