# Supplementary material for: A single-nucleus transcriptomic atlas of the adult Aedes aegypti mosquito
Source: bioRxiv. 2025 Sep 30:2025.02.25.639765. Originally published 2025 Feb 25. Preprint. [Version 3] doi: 10.1101/2025.02.25.639765 (PMC11888250; doi:10.1101/2025.02.25.639765)
Supplement: 9 [file NIHPP2025.02.25.639765v3-supplement-9.pdf]

bioRxiv preprint doi: https://doi.org/10.1101/2025.02.25.639765; this version posted September 30, 2025. The copyright holder for this preprint (which was not certified by peer review) is the author/funder, who has granted bioRxiv a license to display the preprint in perpetuity. It is made available under aCC-BY 4.0 International license.

Supplemental figures and legends

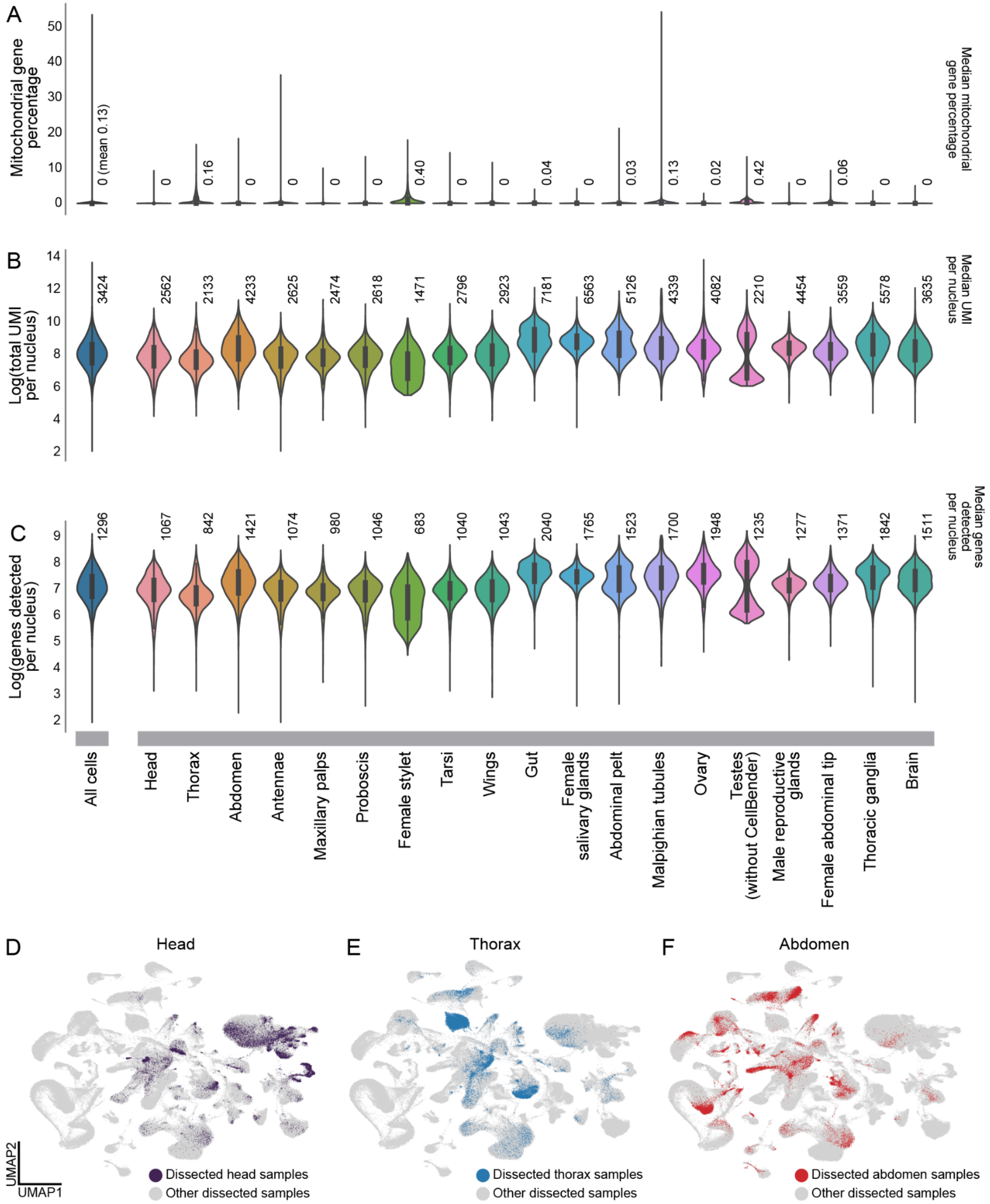

bioRxiv preprint doi: https://doi.org/10.1101/2025.02.25.639765; this version posted September 30, 2025. The copyright holder for this preprint (which was not certified by peer review) is the author/funder, who has granted bioRxiv a license to display the preprint in perpetuity. It is made available under aCC-BY 4.0 International license.

Figure S1. Final data quality control post filtering, related to Figure 1.
(A-C) Violin box plots depicting data (post-quality control filtering) mitochondrial gene percentage (A), total unique molecular identifiers (UMI) per nuclei (B), and genes detected per cell (C) for all cells (left) and for each tissue (right). Across all nuclei from all tissues, median mitochondrial gene percentage was 0.00% (mean 0.13%), median total UMI per nucleus was 3,424, and median genes per nucleus was 1,296. Annotations above each violin represent median values unless otherwise indicated. Pre-filtering gene per nucleus and UMI per nucleus metrics are available in Table S1, as well as filtering parameters. Note clusters were filtered by quality control metrics on clusters, not filtered on individual cell quality control metrics (Data S1, Table S1). Multiple samples (10x Genomics libraries) from males and females are included for each tissue. Inner boxes represent first quartile to third quartile. Box plot lines represent 1.5x interquartile range. Length of violin indicates complete range of the data, with thickness of the violin representing number of cells at each value.
(D-F) UMAPs of integrated *Aedes aegypti* Mosquito Cell Atlas data, colored by samples representing major body parts: female and male head (D), thorax (E), and abdomen (F).

bioRxiv preprint doi: https://doi.org/10.1101/2025.02.25.639765; this version posted September 30, 2025. The copyright holder for this preprint (which was not certified by peer review) is the author/funder, who has granted bioRxiv a license to display the preprint in perpetuity. It is made available under aCC-BY 4.0 International license.

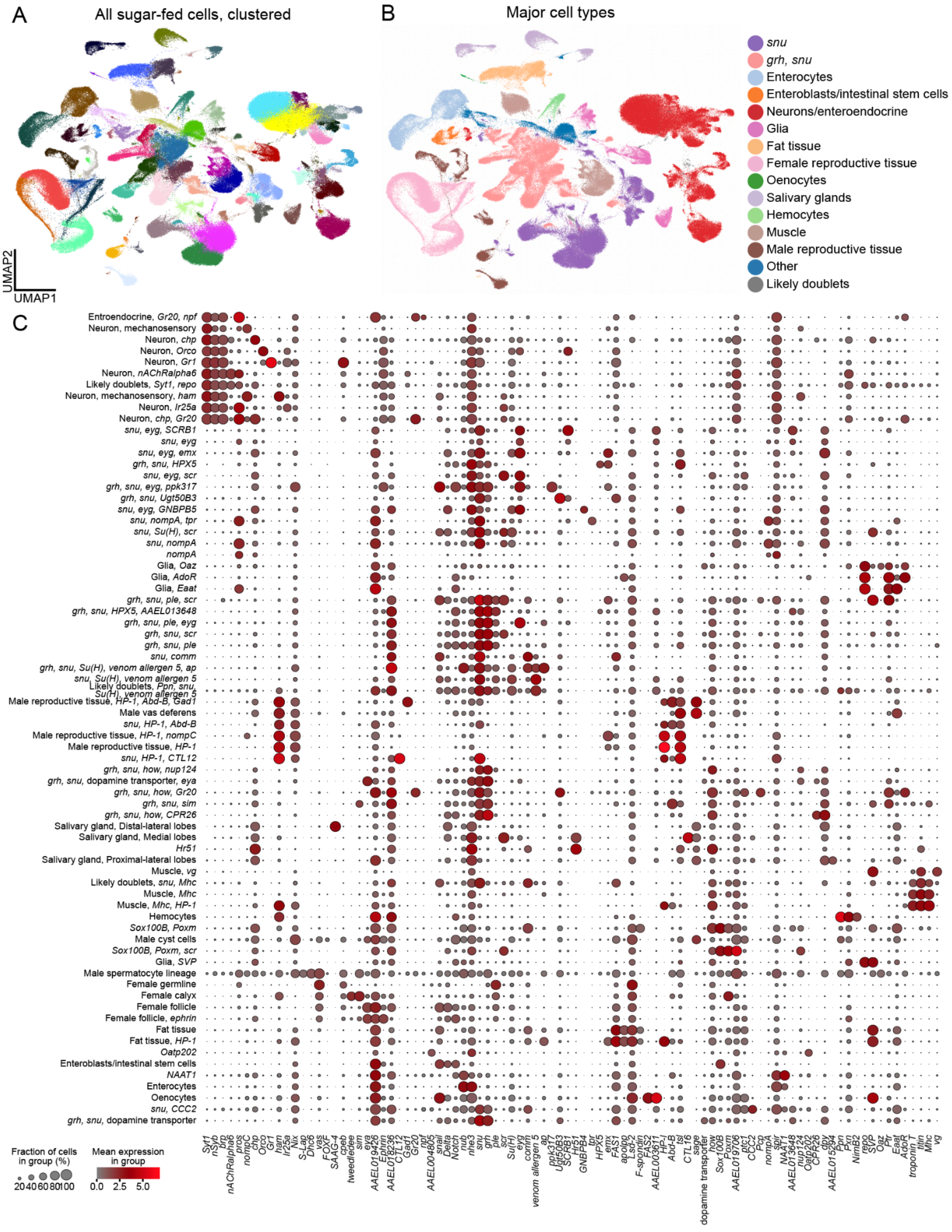

Figure S2. Integrated *Aedes aegypti* Mosquito Cell Atlas tissues and data, related to Figure 1.

bioRxiv preprint doi: https://doi.org/10.1101/2025.02.25.639765; this version posted September 30, 2025. The copyright holder for this preprint (which was not certified by peer review) is the author/funder, who has granted bioRxiv a license to display the preprint in perpetuity. It is made available under aCC-BY 4.0 International license.

(A-B) UMAPs of integrated *Aedes aegypti* Mosquito Cell Atlas data colored by clustering (Louvain algorithm, resolution = 1) (A) and major cell types based on manual annotation in Figure 1F (B).
(C) Dot plot of integrated *Aedes aegypti* Mosquito Cell Atlas annotations and gene markers. Color scale indicates mean normalized expression of gene within cell type, size of dot indicates percent of cells expressing gene within the group. See Table S1 for gene IDs and annotation thresholds.

bioRxiv preprint doi: https://doi.org/10.1101/2025.02.25.639765; this version posted September 30, 2025. The copyright holder for this preprint (which was not certified by peer review) is the author/funder, who has granted bioRxiv a license to display the preprint in perpetuity. It is made available under aCC-BY 4.0 International license.

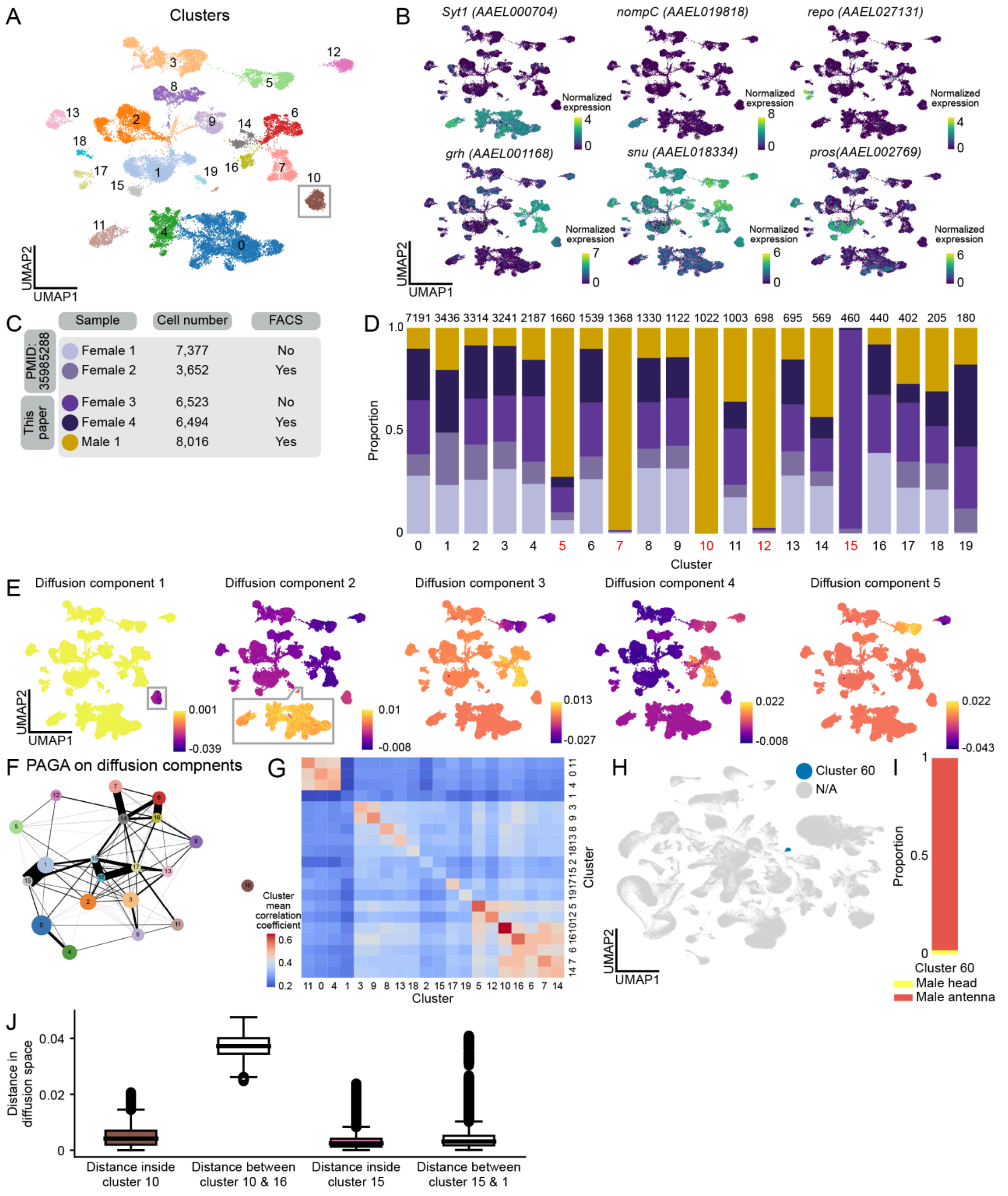

bioRxiv preprint doi: https://doi.org/10.1101/2025.02.25.639765; this version posted September 30, 2025. The copyright holder for this preprint (which was not certified by peer review) is the author/funder, who has granted bioRxiv a license to display the preprint in perpetuity. It is made available under aCC-BY 4.0 International license.

Figure S3. Identification of male-specific *ppk317* cell type in *Aedes aegypti* antenna, related to Figure 3.

(A) UMAP of antenna nuclei clustered and numbered using the Leiden algorithm (resolution = 0.1). Cluster 10 (male-specific, *ppk317*-expressing cells) highlighted in grey.

(B) Normalized expression of *Syt1, nompC, repo (AAEL027131), grh (AAEL001168), snu (AAEL018334),* and *pros (AAEL002769)*. Normalized expression is *ln([(raw count/total cell counts)*median total counts across cells]+1)*.

(C) Number of cells in each sample (female = 4, male = 1), data source, and if each sample underwent fluorescence-activated cell sorting (FACS).

(D) Stacked bar plot illustrating proportion of each sample within each cluster. Annotated information: cluster numbers (below bar plot), clusters for which over 70% originate from a single sample (red), number of cells in each cluster (above bar plot).

(E) UMAPs of diffusion components 1 through 5. Diffusion component 1 (first panel) maps to cluster 10 (*ppk317*-expressing cells, highlighted in grey), suggesting a robust biological feature. Diffusion component 2 (second panel) maps to neurons (highlighted in grey).

(F) Partition-based graph abstraction (PAGA) calculated on diffusion components in (E). All edges illustrated, no edge threshold set.

(G) Correlation matrix heatmap, depicting pairwise correlation of gene expression matrices between each cluster (mean Pearson correlation coefficient). Diagonal values (cluster to itself) represents intra-cluster correlation values, which vary based on features such as cell number and transcriptome heterogeneity.

(H) UMAP of integrated *Aedes aegypti* Mosquito Cell Atlas data with cluster 60 colored in blue (Louvain algorithm, resolution = 0.1). *ppk317*-expressing antennal cells belong to cluster 60, see Figure 3E.

(I) Stacked bar plot indicating tissue origin of cells from cluster 60. Cluster 60 come from the male antenna and male head sample.

(J) Pairwise Euclidean distances on diffusion embeddings from (E) to approximate phenotypic distance between and within clusters. Boxes represent first quartile to third quartile, and middle line represents median. Whiskers represent the 1.5x interquartile range of the data, with points outside this range as outliers.

bioRxiv preprint doi: https://doi.org/10.1101/2025.02.25.639765; this version posted September 30, 2025. The copyright holder for this preprint (which was not certified by peer review) is the author/funder, who has granted bioRxiv a license to display the preprint in perpetuity. It is made available under aCC-BY 4.0 International license.

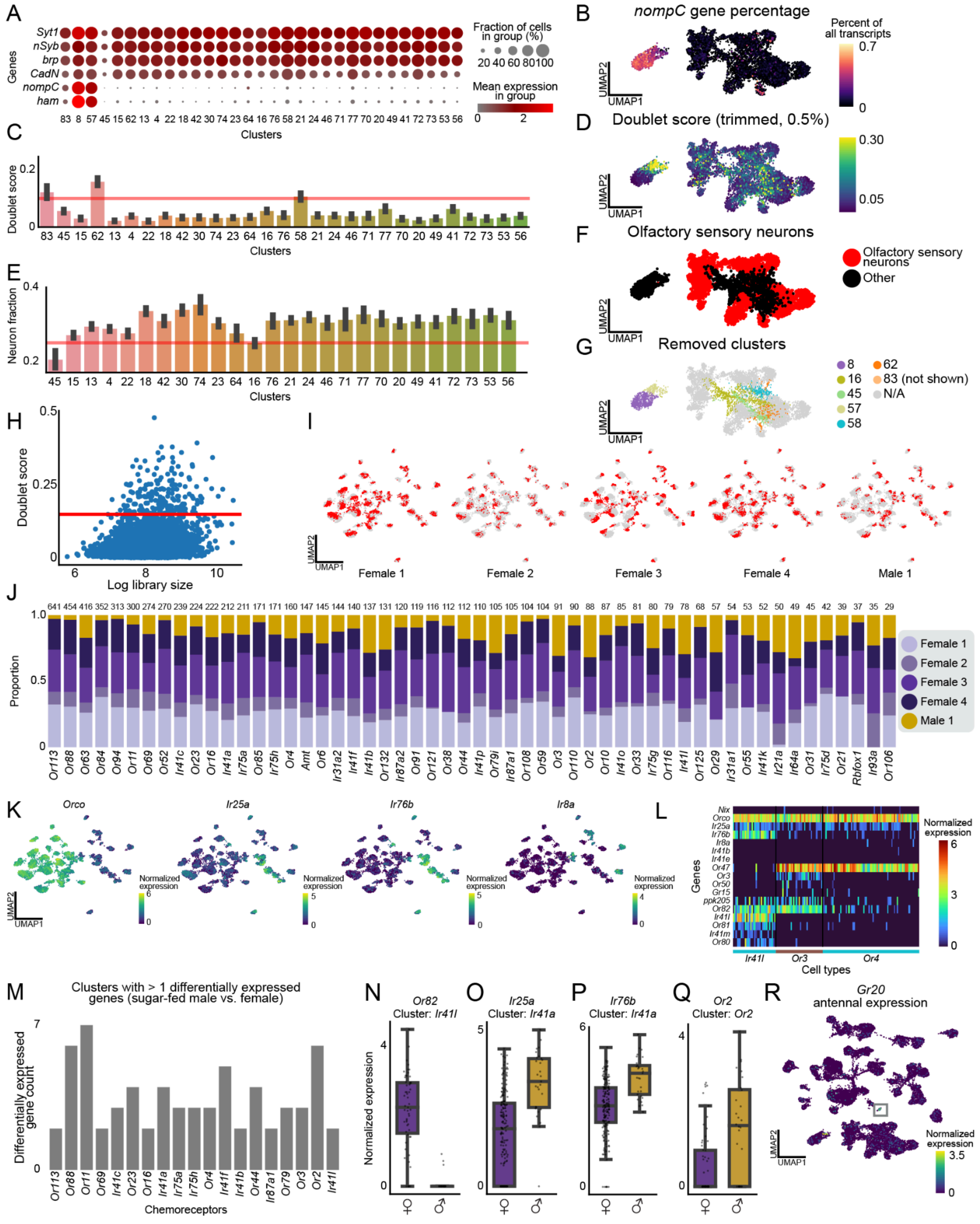

Figure S4. Antenna chemosensory cell type expressing *Orco* and *Ir25a* is sexually dimorphic for *Or82,* related to Figure 4.

(A) Dot plot illustrating mean normalized expression of neuronal genes set: *Syt1, nSyb,*

bioRxiv preprint doi: https://doi.org/10.1101/2025.02.25.639765; this version posted September 30, 2025. The copyright holder for this preprint (which was not certified by peer review) is the author/funder, who has granted bioRxiv a license to display the preprint in perpetuity. It is made available under aCC-BY 4.0 International license.

brp (AAEL018153), CadN, nompC, and *ham (AAEL017229)*. Size of dot indicated the percent of cells in each group, color indicated mean normalized expression. Normalized expression is *ln([(raw count/total cell counts)*median total counts across cells]+1)*.
(B) Fraction of total transcripts per cell of *nompC* in antennal neuronal nuclei. (B,D,F,G) use the same UMAP coordinates as neurons from Figure 4A. UMAPs cropped for space, cluster 83 not shown.
(C) Mean doublet score across cells, with error bars indicating 95% confidence interval calculated from bootstrapping. Generated through scrublet[189]. Clusters with an average score above 0.15 were removed from further analysis (red line).
(D) UMAP depicting doublet score.
(E) Average percentage of neuronal genes in (A), with error bars indicating 95% confidence interval calculated from bootstrapping. Clusters with an average score below 0.25 were removed from further analysis (red line).
(F) UMAP demonstrating which clusters (Leiden, resolution = 5) were kept for downstream analysis (red) or removed based on filtering parameters (black).
(G) UMAP of neurons from antenna samples, demonstrating clusters removed from downstream analysis.
(H) Log(library size) versus calculated doublet score for neurons filtered in (A-G). Cells with a score above 0.15 were removed from further analysis (red line).
(I) UMAPs of antenna olfactory sensory neurons (filtered *nompC*-negative sensory neuron population), colored by sample.
(J) Stacked bar plot illustrating proportion of each sample within each cluster in annotated *nompC*-negative sensory neuron population. Annotated information: cluster numbers (below bar plot), number of cells in each cluster (above bar plot). No clusters has more than 70% of cells from a single sample.
(K) Normalized gene expression of olfactory co-receptor genes: *Orco, Ir25a, Ir76b,* and *Ir8a*.
(L) Heatmap of *Ir41l*, *Or3*, and *Or4* cells from female samples 3 and 4 (see Figure S3C). Selected genes are indicated in rows, cells in columns, with cell type annotations below. Heatmap colors represent normalized expression.
(M) Bar plot showing olfactory sensory neuron cell types with at least 2 differentially expressed genes (DEGs) between male and female cells. Significant genes had |log fold change| >1, false discovery rate <0.05, determined by MAST on normalized expression. For more information on DEGs, see Table S2.
(N-Q) Distribution of expression for differentially expressed olfactory receptor genes across male and female cells in a particular cluster. *Or82* expression in cluster *Ir41l* (N), *Ir25a* in cluster *Ir41a* (O), *Ir76b* in cluster *Ir41a* (P), *Or2* expression in cluster *Or2* (Q). Boxes represent first quartile to third quartile, and middle line represents median. Whiskers represent the 1.5x interquartile range of the data, with points outside this range as outliers.
(R) *Gr20* normalized expression. *Gr20* positive antennal cluster highlighted in grey.

bioRxiv preprint doi: https://doi.org/10.1101/2025.02.25.639765; this version posted September 30, 2025. The copyright holder for this preprint (which was not certified by peer review) is the author/funder, who has granted bioRxiv a license to display the preprint in perpetuity. It is made available under aCC-BY 4.0 International license.

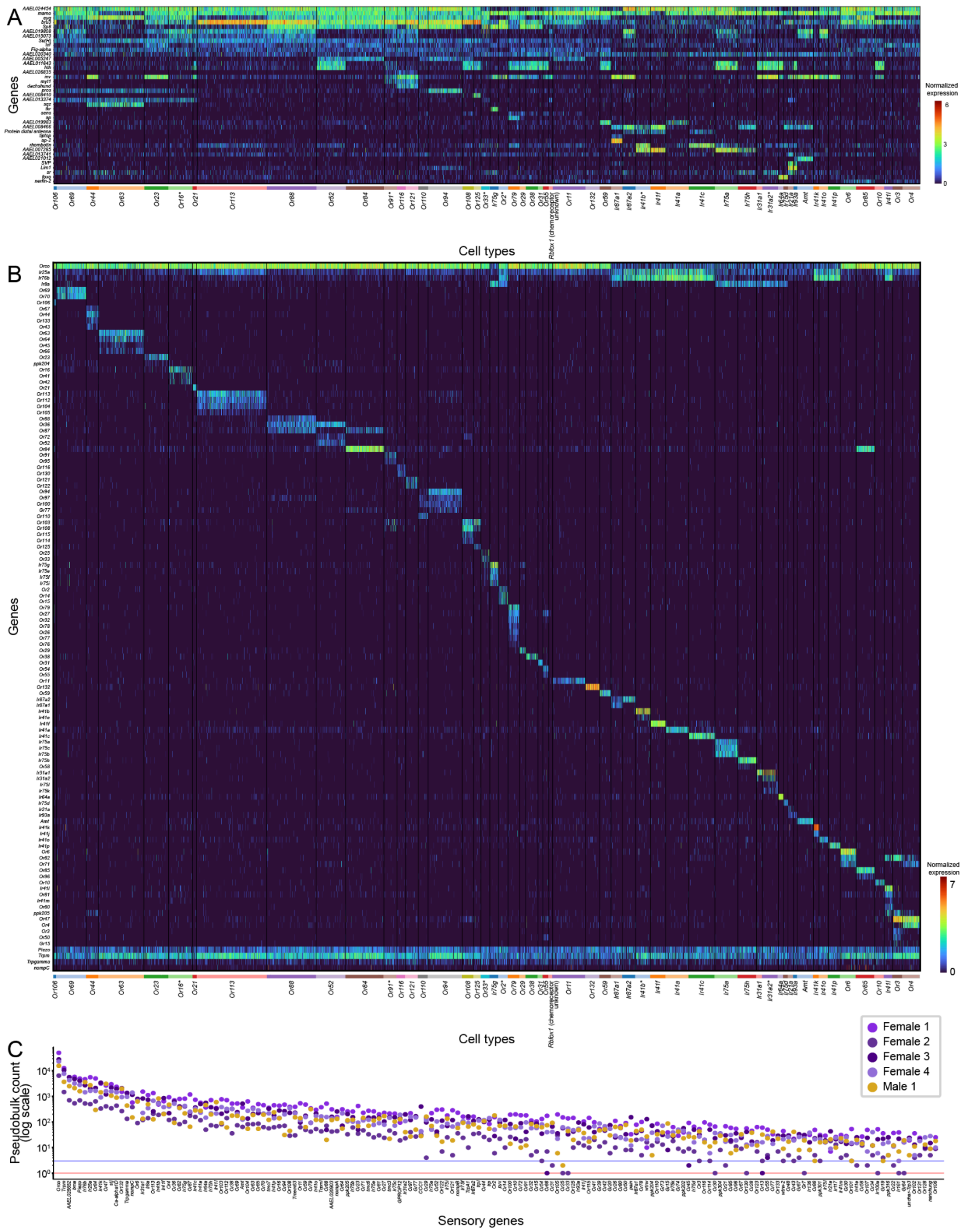

Figure S5. Antenna chemosensory neuron chemoreceptor and putative transcription factor factor expression profiles, related to Figure 4.
(A-B) Heatmap of all annotated antenna olfactory sensory neuron cell types for selected

bioRxiv preprint doi: https://doi.org/10.1101/2025.02.25.639765; this version posted September 30, 2025. The copyright holder for this preprint (which was not certified by peer review) is the author/funder, who has granted bioRxiv a license to display the preprint in perpetuity. It is made available under aCC-BY 4.0 International license.

putative transcription factors (A) and selected sensory genes (B). Selected genes are indicated in rows, cells in columns, with cell type annotations below. Cells are grouped by annotation in Figure 4C and indicated below heatmap. Asterisks (*) on cell type annotation indicates that a group may represent a co-clustering of cells with multiple unique sensory gene expression patterns, as depicted in Figure 4C-4D. Heatmap colors represent normalized expression. Normalized expression is *ln([(raw count/total cell counts)*median total counts across cells]+1)*. See Table S1 for gene IDs and annotation thresholds.

(C) Scatterplot of summed raw counts of sensory genes within all olfactory sensory neurons within each antenna sample. Red line indicates count of 1, blue line is a count of 5. 158 out of 403 putative sensory genes (Table S1) with the highest counts shown for space (full plot available in Zenodo Supplemental Data).

bioRxiv preprint doi: https://doi.org/10.1101/2025.02.25.639765; this version posted September 30, 2025. The copyright holder for this preprint (which was not certified by peer review) is the author/funder, who has granted bioRxiv a license to display the preprint in perpetuity. It is made available under aCC-BY 4.0 International license.

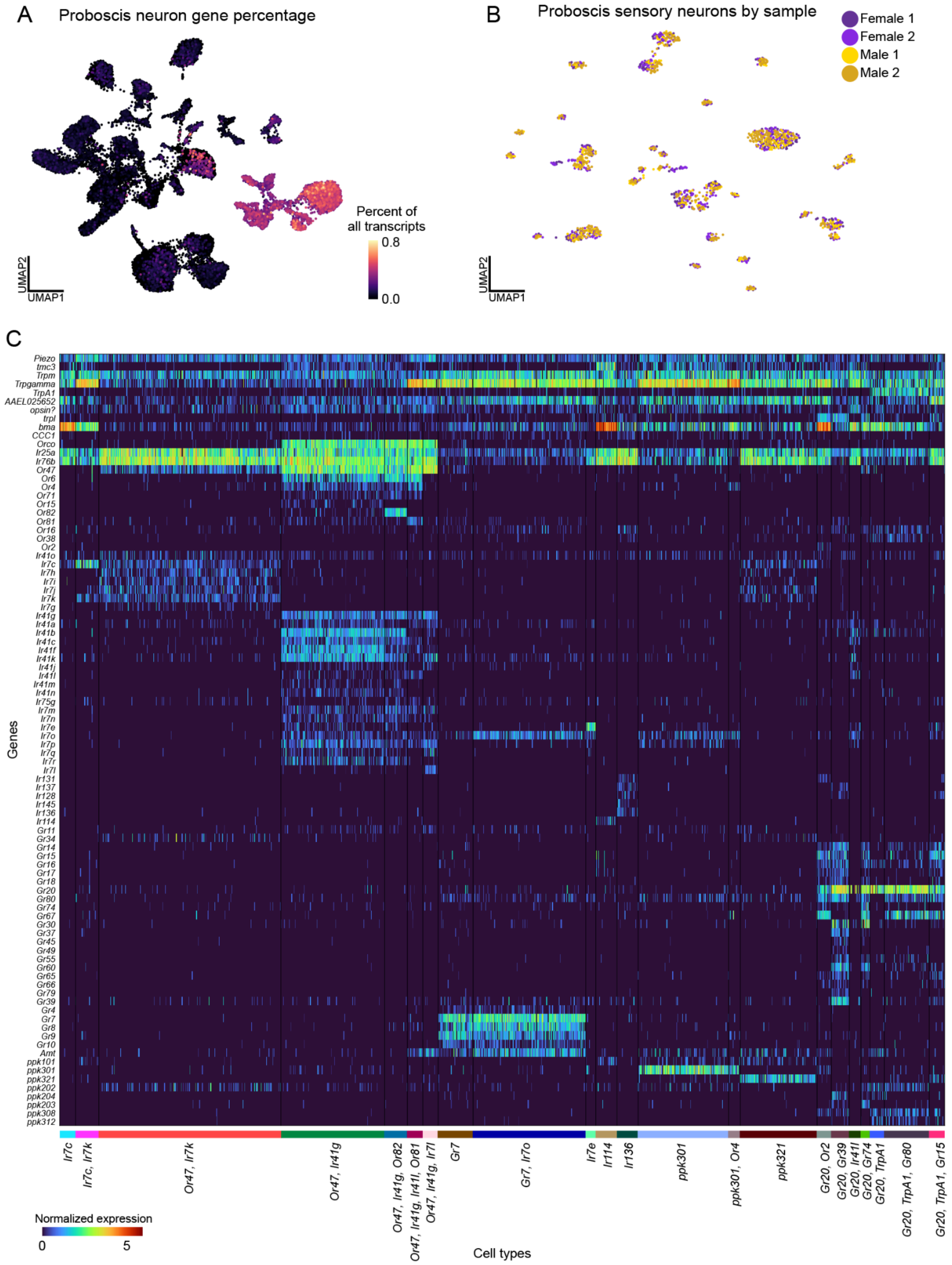

Figure S6. Proboscis sensory gene analysis, related to Figures 4 and 5.
(A) UMAP of proboscis cells illustrating fraction of total transcripts per cell of neuronal genes set: *Syt1*, *brp*, *nSyb*, *CadN*. *nompC*-negative cells highlighted (grey box). For

bioRxiv preprint doi: https://doi.org/10.1101/2025.02.25.639765; this version posted September 30, 2025. The copyright holder for this preprint (which was not certified by peer review) is the author/funder, who has granted bioRxiv a license to display the preprint in perpetuity. It is made available under aCC-BY 4.0 International license.

nompC gene percentage, see Data S4.
(B) UMAP of reclustered proboscis *nompC*-negative sensory cells colored by sample (female = 2, male = 2).
(C) Heatmap of cells from all annotated clusters. Sensory genes are indicated in rows and cells indicated in columns. Selected genes are indicated in rows, cells in columns, with cell type annotations below. Heatmap colors represent normalized expression. Normalized expression is *ln([(raw count/total cell counts)*median total counts across cells]+1)*.

bioRxiv preprint doi: https://doi.org/10.1101/2025.02.25.639765; this version posted September 30, 2025. The copyright holder for this preprint (which was not certified by peer review) is the author/funder, who has granted bioRxiv a license to display the preprint in perpetuity. It is made available under aCC-BY 4.0 International license.

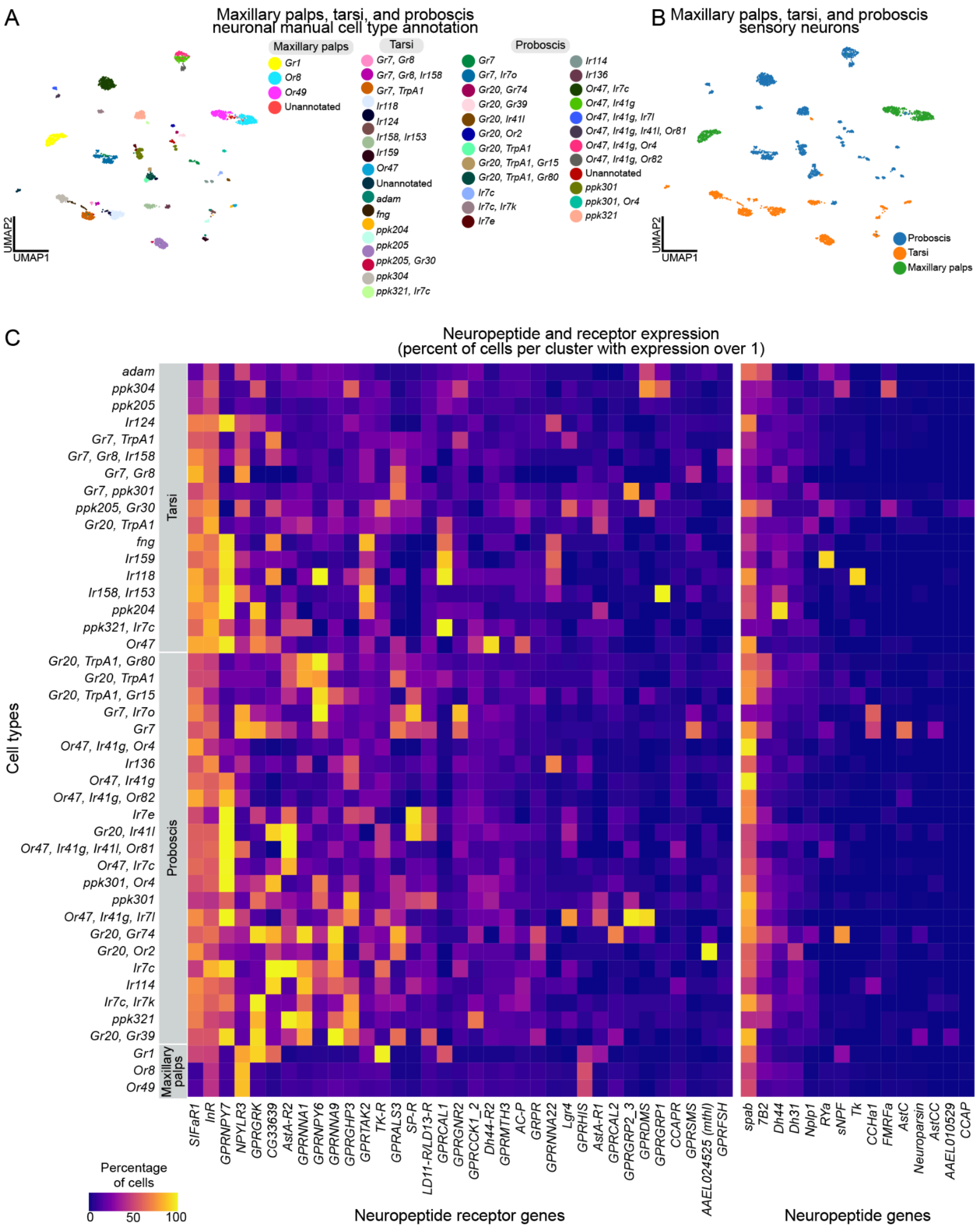

Figure S7. Neuropeptide receptor and synthesis gene analysis, related to Figures 4 and 5.
(A-B) UMAP of *nompC*-negative sensory neurons from maxillary palps, tarsi and

bioRxiv preprint doi: https://doi.org/10.1101/2025.02.25.639765; this version posted September 30, 2025. The copyright holder for this preprint (which was not certified by peer review) is the author/funder, who has granted bioRxiv a license to display the preprint in perpetuity. It is made available under aCC-BY 4.0 International license.

proboscis samples, colored by manual cell type annotation as listed in legend at the right of the figure panel **(A)** and original tissue **(B)**.
(C) Heatmap of expression of neuropeptide receptor genes (left) and neuropeptide synthesis genes (right) within annotated *nompC*-negative sensory neurons in the maxillary palps, tarsi and proboscis. Color scale indicates percentage of cells expressing a gene above threshold (normalized expression value of 1). Sensory genes are indicated in columns and annotated cell types indicated in rows. Genes were included if they were expressed above threshold in over 20% of cells in at least one cell type. Genes filtered from lists in Table S1. Normalized expression is *ln([(raw count/total cell counts)*median total counts across cells]+1)*.

bioRxiv preprint doi: https://doi.org/10.1101/2025.02.25.639765; this version posted September 30, 2025. The copyright holder for this preprint (which was not certified by peer review) is the author/funder, who has granted bioRxiv a license to display the preprint in perpetuity. It is made available under aCC-BY 4.0 International license.

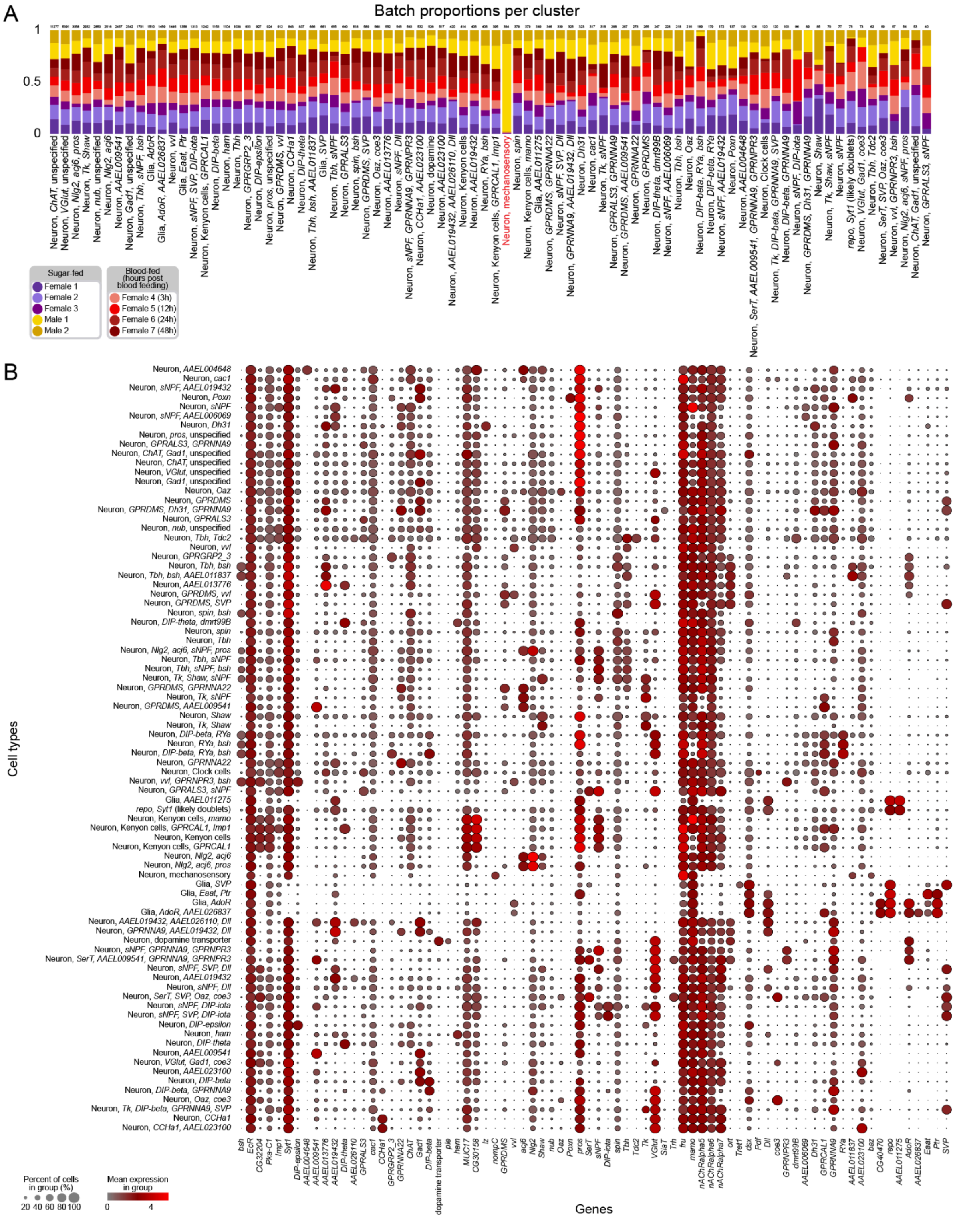

Figure S8. Brain Cell types, related to Figures 6 and 7.
(A) Stacked bar plot illustrating proportion of each sample within each cluster for brain nuclei cell types. Annotated information: cluster numbers (below bar plot), clusters for

bioRxiv preprint doi: https://doi.org/10.1101/2025.02.25.639765; this version posted September 30, 2025. The copyright holder for this preprint (which was not certified by peer review) is the author/funder, who has granted bioRxiv a license to display the preprint in perpetuity. It is made available under aCC-BY 4.0 International license.

which over 70% originate from a single sample (red), number of cells in each cluster (above bar plot).
(B) Dot plot illustrating mean normalized expression of gene markers. Color scale indicates mean normalized expression of gene within cell type, size of dot indicates percent of cells expressing gene within the group. See Table S1 for gene IDs and annotation thresholds.

bioRxiv preprint doi: https://doi.org/10.1101/2025.02.25.639765; this version posted September 30, 2025. The copyright holder for this preprint (which was not certified by peer review) is the author/funder, who has granted bioRxiv a license to display the preprint in perpetuity. It is made available under aCC-BY 4.0 International license.

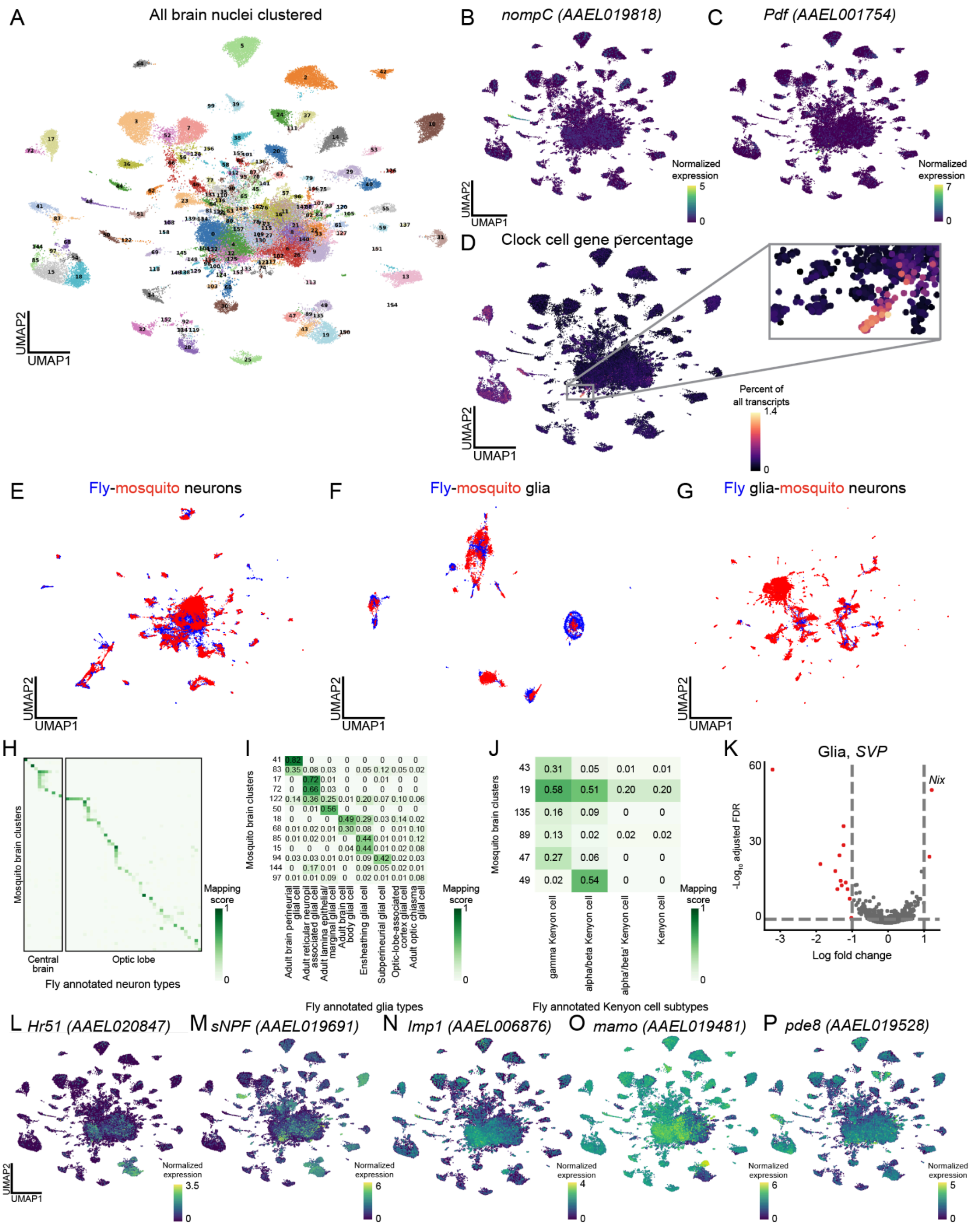

Figure S9. SAMap analysis and identification of clock cells and kenyon cells, related to Figure 6.
(A) UMAP of brain nuclei clustered using the Leiden algorithm (resolution = 5).

bioRxiv preprint doi: https://doi.org/10.1101/2025.02.25.639765; this version posted September 30, 2025. The copyright holder for this preprint (which was not certified by peer review) is the author/funder, who has granted bioRxiv a license to display the preprint in perpetuity. It is made available under aCC-BY 4.0 International license.

(B-C) Normalized expression of *nompC* (B) and *Pdf (AAEL001754)* (C). Normalized expression is *ln([(raw count/total cell counts)*median total counts across cells]+1)*.
(D) Fraction of total transcripts per cell of 10 putative clock cell gene markers (Table S1). Cluster with high expression highlighted in grey box, enlarged in inset.
(E-G) UMAP of manifold integration of snRNA-seq data from *Aedes aegypti* mosquito brain and published *Drosophila melanogaster* fly head[18]. Plots show integration of fly neurons with mosquito neurons (E), fly glia with mosquito glia (F), and, as a control, fly glia with mosquito neuron (G). Alignment scores are 0.64, 0.64 and 0.47, respectively.
(H-J) Correlation matrices of mapping scores between *Drosophila melanogaster* head annotations and *Aedes aegypti* of clusters (Leiden, resolution = 5) for neuronal cell types (H), glial cell types (I), and Kenyon cell subtypes (J). For all numerical values, see Table S3.
(K) Volcano plot of differentially expressed genes between male and female cells in the *SVP* glia *(AAEL002765*) cluster. Significant genes (red) had |log fold change| >1, false discovery rate <0.05, determined by MAST on normalized expression (Table S2). Male biased genes on right, indicated by *Nix* (*AAEL022912*).
(L-P) Normalized gene expression of *Hr51 (AAEL020847)* (L), *sNPF (AAEL019691)* (M), *Imp1 (AAEL006876)* (N), *mamo (AAEL019481)* (O), *Pde8 (AAEL019528)* (P).

bioRxiv preprint doi: https://doi.org/10.1101/2025.02.25.639765; this version posted September 30, 2025. The copyright holder for this preprint (which was not certified by peer review) is the author/funder, who has granted bioRxiv a license to display the preprint in perpetuity. It is made available under aCC-BY 4.0 International license.

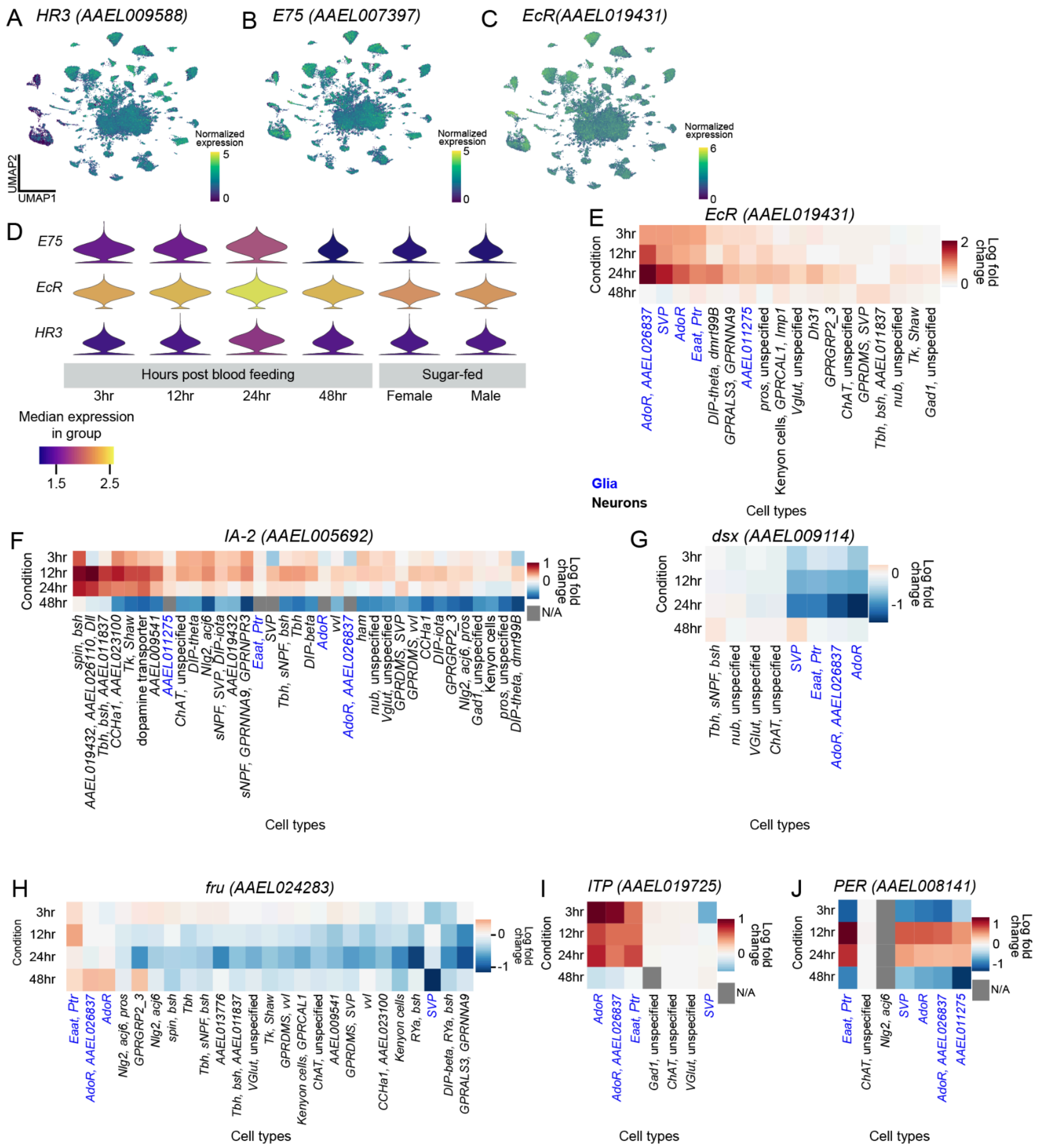

Figure S10. Blood feeding changes in brain, related to Figure 7.

(A-C) Normalized gene expression UMAP of *E75* (A), *EcR* (*AAEL019431*) (B) and *HR3* (C) in all brain nuclei. Normalized expression is *ln([(raw count/total cell counts)*median total counts across cells]+1)*.

(D) Violin plot of gene expression of *E75*, *EcR* and *HR3* across all brain nuclei in each

bioRxiv preprint doi: https://doi.org/10.1101/2025.02.25.639765; this version posted September 30, 2025. The copyright holder for this preprint (which was not certified by peer review) is the author/funder, who has granted bioRxiv a license to display the preprint in perpetuity. It is made available under aCC-BY 4.0 International license.

timepoint. Length of violin indicates complete range of the data, with thickness of the violin representing number of cells at each value.
(E-J) Heatmaps of log fold change of gene expression, grouped by annotated cell type, between corresponding cells collected in each blood-feeding timepoint compared to sugar-fed female brain. Genes shown: *EcR* (E), *IA-2 (AAEL005692)* (F), *dsx* (*AAEL009114*), (G), *fru* (*AAEL024283*) (H), *ITP* (*AAEL019725*) (**I**), and *PER* (*AAEL008141*) (J). Log fold change is determined by MAST on normalized expression. Cell types are sorted by the total log fold change across all timepoints and colored as glia (blue) or neurons (black). Cell types included have over 10 cells in each timepoint, and at least one timepoint where change from sugar-fed condition had a false discovery rate <0.05. Grey boxes indicate log fold change data is not available, due to zero expression within cell type at the specified timepoint (or the sugar-fed condition for *PER* in *Nlg2, acj6* cells).

Supplemental Data and table titles

Data S1. Example sample quality-control filtering, and gene expression patterns in the testes and antimicrobial peptide-expressing tissues, related to Figures 1 and 2.
Data S2. Individual tissue annotations of mated, sugar-fed tissues, related to Figures 1-6.
Data S3. RNA *in situ* hybridization controls and probe information, related to Figures 2-4.
Data S4. Filtering and characterization of *nompC* (*AAEL019818*)-negative sensory neurons, related to Figure 5.
Data S5. *Aedes aegypti* Mosquito Cell Atlas Consortium Members and Funding

Table S1. Sample, processing, annotation, and gene information for the *Aedes aegypti* Mosquito Cell Atlas (related to Figure 1).
Table S2. Differential gene expression for sensory appendage, brain, fat tissue and gut cell types, across sex or bloodfeeding (related to Figures 4-7).
Table S3. Cross-species manifold integration mapping of *Aedes aegypti* brain cells to *Drosophila melanogaster* head cell types (related to Figure 6).
