# Supplementary material for: Mosquito Cell Atlas: A single-nucleus transcriptomic atlas of the adult Aedes aegypti mosquito
Source: bioRxiv. 2025 Feb 25:2025.02.25.639765. Preprint. [Version 1] doi: 10.1101/2025.02.25.639765 (PMC11888250; doi:10.1101/2025.02.25.639765)
Supplement: Supplementary file 1 [file NIHPP2025.02.25.639765V1-supplement-1.pdf]

bioRxiv preprint doi: https://doi.org/10.1101/2025.02.25.639765; this version posted February 25, 2025. The copyright holder for this preprint (which was not certified by peer review) is the author/funder, who has granted bioRxiv a license to display the preprint in perpetuity. It is made available under aCC-BY 4.0 International license.

Supplemental figures

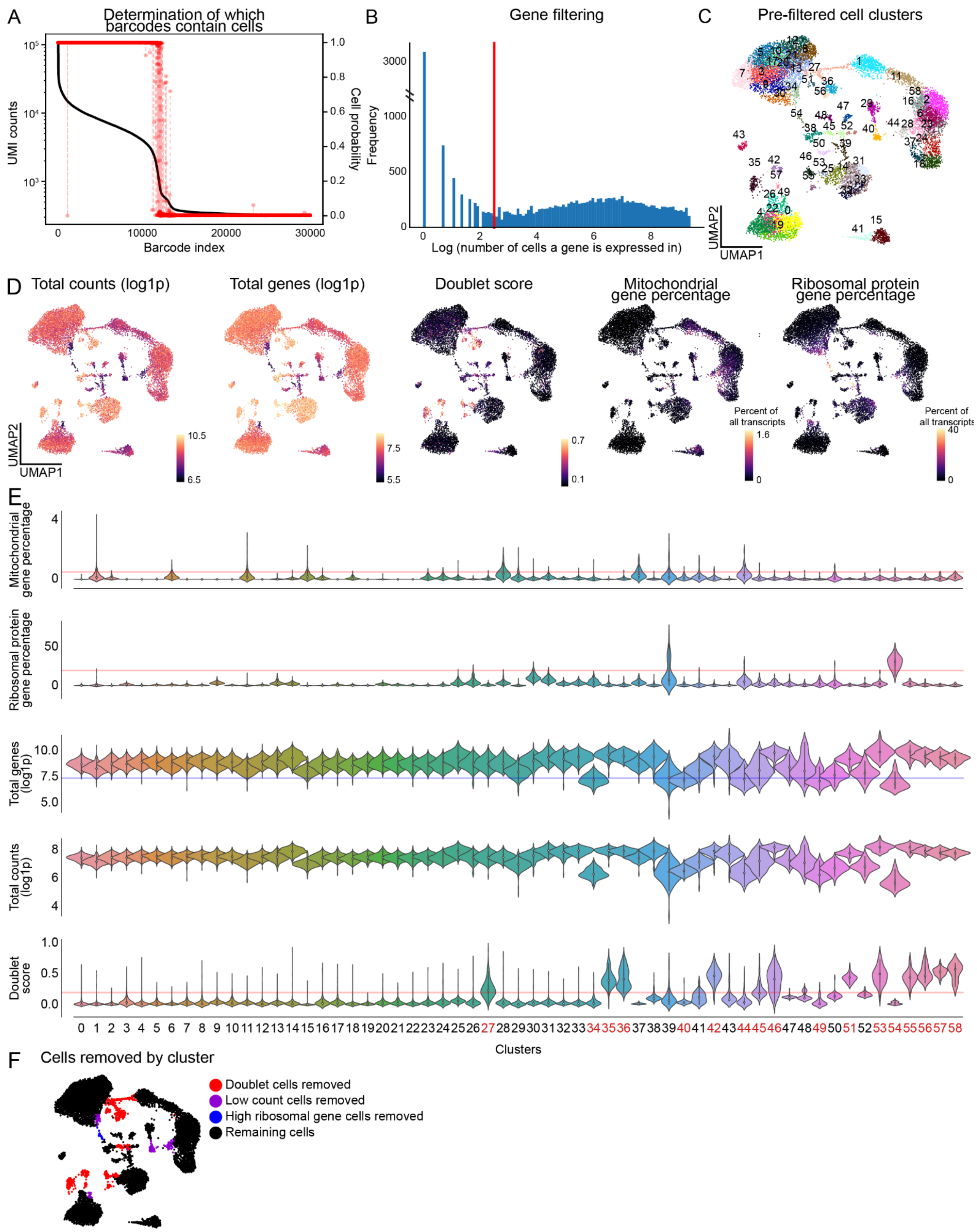

bioRxiv preprint doi: https://doi.org/10.1101/2025.02.25.639765; this version posted February 25, 2025. The copyright holder for this preprint (which was not certified by peer review) is the author/funder, who has granted bioRxiv a license to display the preprint in perpetuity. It is made available under aCC-BY 4.0 International license.

Figure S1. Processing of female salivary gland sample, related to Figures 1 and 3.
(A) Determination of individual cell barcodes that contain cells by CellBender[207]. Unique molecular identifier (UMI) counts (black, left axis), probability that a barcode index is a cell (red, right axis). Low probability cells were removed by the CellBender.
(B) Histogram of number of cells that an individual gene is expressed in (log scale). Genes expressed in fewer than 13 cells were removed from a given sample (red line).
(C) UMAP of salivary gland cells clustered using the Leiden algorithm (resolution = 5) for quality control and cell filtering.
(D-E) UMAPs **(D)** and violin plots **(E)** depicting total counts, total genes, doublet score (generated through scrublet[189], mitochondrial gene percentage, ribosomal gene percentages (log1p, indicates a pseudocount of 1 was used for log-scaling) for each cell and across each cluster. Lines in (E) indicate thresholds by which clusters with an average value above (red) or below (blue) were removed from further analysis. Removed clusters indicated in red on the bottom of (E).
(F) UMAP colored by cluster that were kept for further analysis (black) or removed due to quality control metrics depicted in (D-E).
(G) UMAP of ovary samples female 1 (dark purple) and female 2 (light purple) prior to merging.

bioRxiv preprint doi: https://doi.org/10.1101/2025.02.25.639765; this version posted February 25, 2025. The copyright holder for this preprint (which was not certified by peer review) is the author/funder, who has granted bioRxiv a license to display the preprint in perpetuity. It is made available under aCC-BY 4.0 International license.

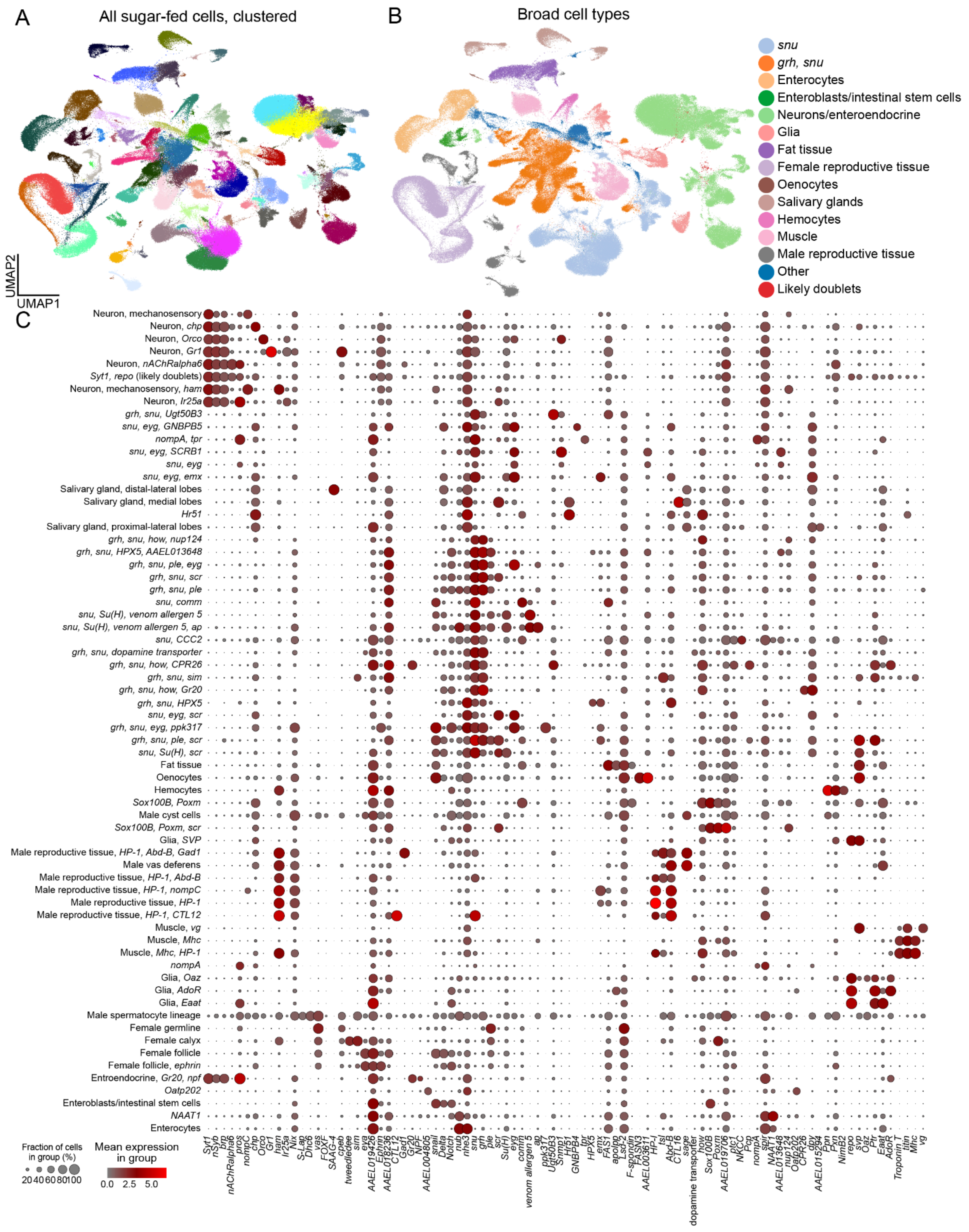

bioRxiv preprint doi: https://doi.org/10.1101/2025.02.25.639765; this version posted February 25, 2025. The copyright holder for this preprint (which was not certified by peer review) is the author/funder, who has granted bioRxiv a license to display the preprint in perpetuity. It is made available under aCC-BY 4.0 International license.

Figure S2. **Integrated mosquito cell atlas tissues and data, related to Figure 1.**
(A-B) UMAPs of integrated mosquito cell atlas data colored by clustering (Louvain algorithm, resolution = 1) (A) and broad cell-types based on manual annotation in Figure 1F (B).
(C) Dotplot of integrated mosquito cell atlas annotations and gene markers. Color scale indicates mean normalized expression of gene within cell type, size of dot indicates percent of cells expressing gene within the group. See Table S3 and Table S4 for gene IDs and thresholds.

bioRxiv preprint doi: https://doi.org/10.1101/2025.02.25.639765; this version posted February 25, 2025. The copyright holder for this preprint (which was not certified by peer review) is the author/funder, who has granted bioRxiv a license to display the preprint in perpetuity. It is made available under aCC-BY 4.0 International license.

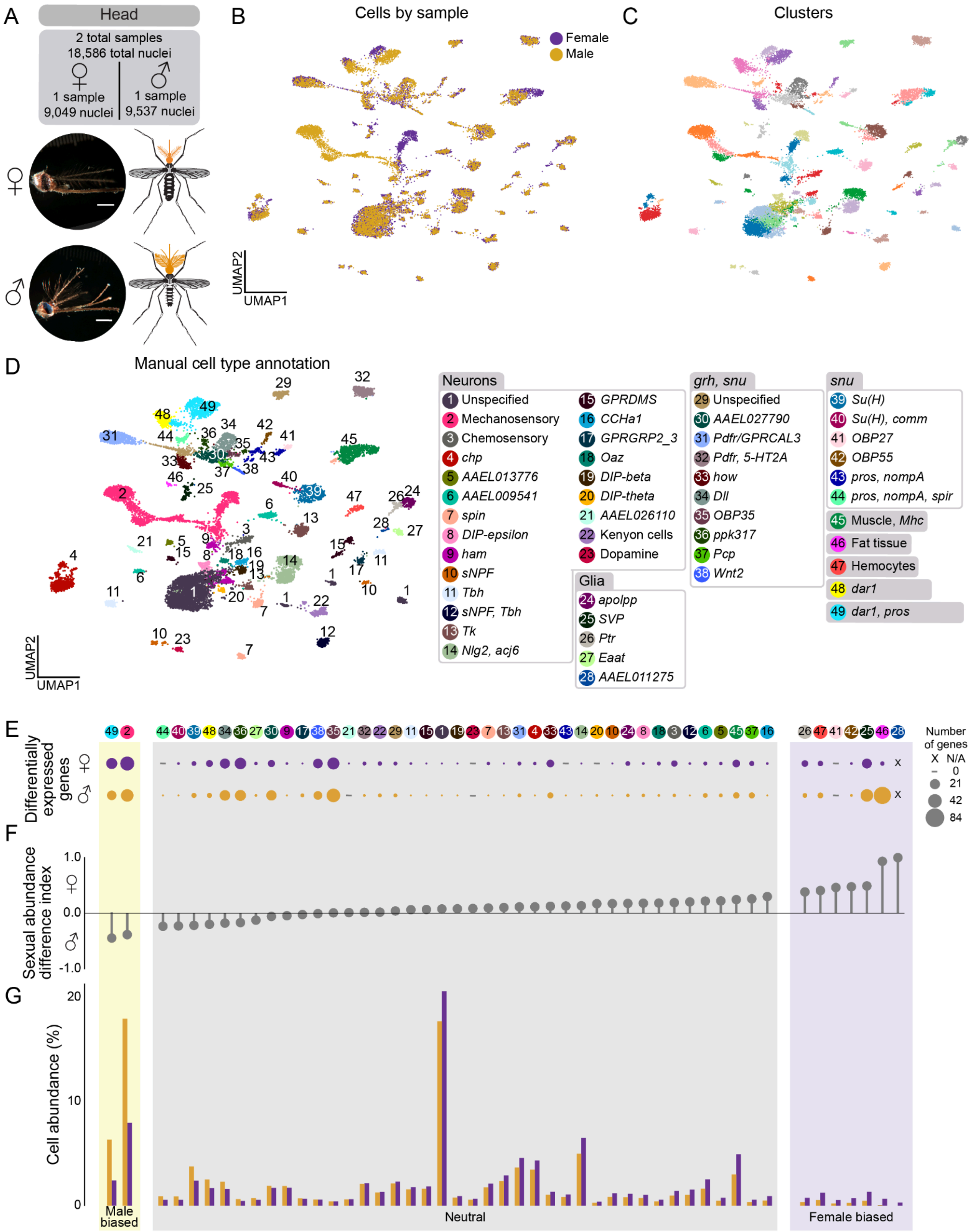

bioRxiv preprint doi: https://doi.org/10.1101/2025.02.25.639765; this version posted February 25, 2025. The copyright holder for this preprint (which was not certified by peer review) is the author/funder, who has granted bioRxiv a license to display the preprint in perpetuity. It is made available under aCC-BY 4.0 International license.

Figure S3. Annotation and differentially-expressed genes in male and female heads, related to Figure 1.
(A) Photo of dissected female (top) and male (bottom) heads with anatomical diagram of head dissection (in orange), and collected sample information. Scale bar: 500 µm.
(B) UMAP of head nuclei, colored by sample (female samples = 1, male samples = 1).
(C) UMAP of head cells clustered using the Leiden algorithm (resolution = 5) for annotation.
(D) UMAP of nuclei from both head samples, colored and numbered by manual annotation using selected marker genes as listed in legend at the right of the figure panel. See Table S3 and Table S4 for gene IDs and thresholds.
(E-G) Quantification of differences between male and female samples. Numbered and colored circles above (E) correspond to annotations in (D).
(E) Dot plot of number of differentially expressed genes between females and males in each cell type. Dots represent relative number of differentially expressed genes (DEG) upregulated in male or female with a |log fold change| > 1 and false discovery rate < 0.05, determined by MAST on normalized expression and converted to log_2 fold change.
(F) Sexual abundance difference index from data in (G). Each cell type was categorized based on the following index: Female biased (purple) if 0.3 < abundance index; Neutral (grey) if -0.3 =< abundance index =< 0.3; Male biased (yellow) if abundance index < -0.3.
(G) Bar plot of relative cell abundance, represented as the percent of each cell type of all nuclei collected from each sex for tissue. Separated by female (purple) and male (yellow).

bioRxiv preprint doi: https://doi.org/10.1101/2025.02.25.639765; this version posted February 25, 2025. The copyright holder for this preprint (which was not certified by peer review) is the author/funder, who has granted bioRxiv a license to display the preprint in perpetuity. It is made available under aCC-BY 4.0 International license.

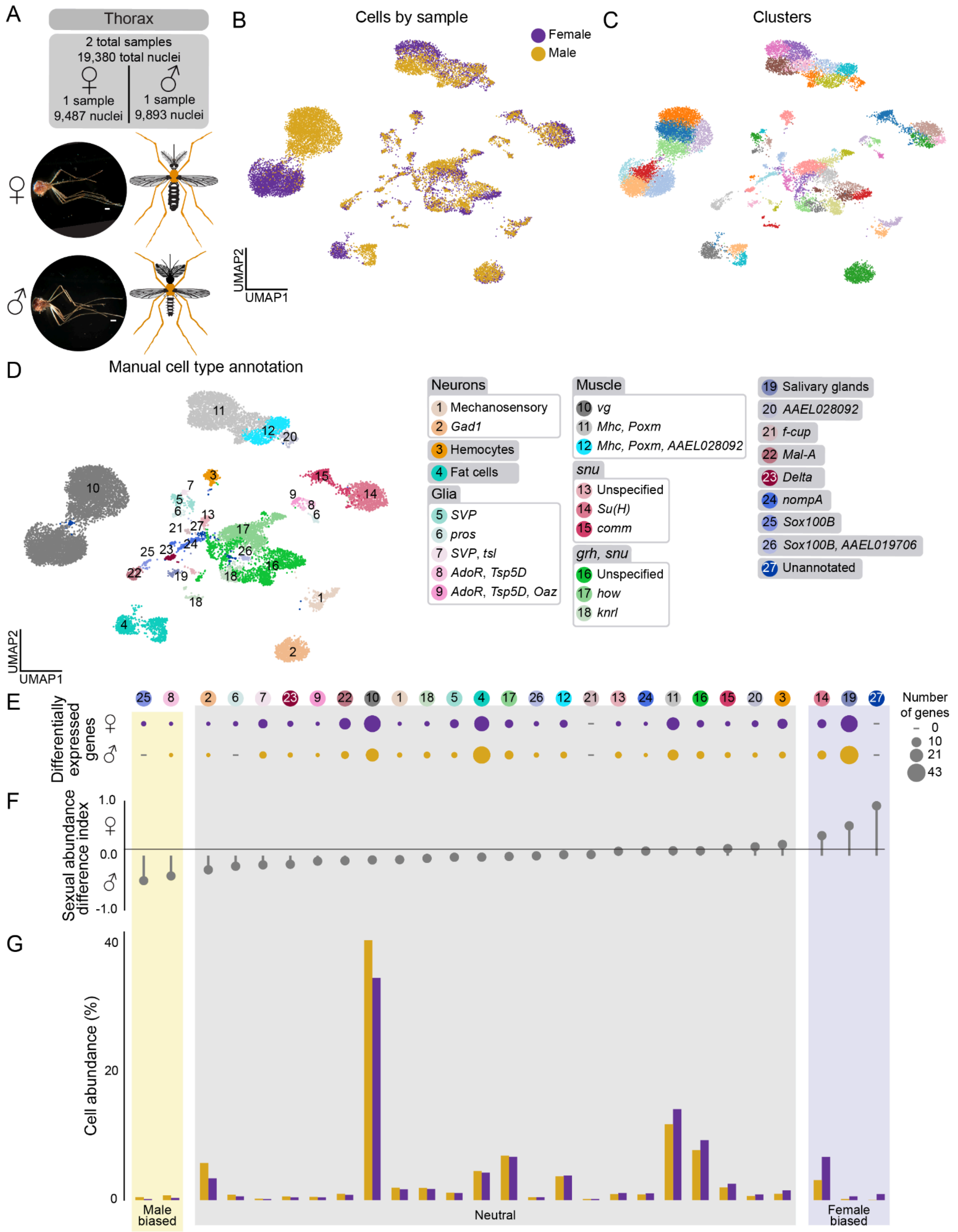

bioRxiv preprint doi: https://doi.org/10.1101/2025.02.25.639765; this version posted February 25, 2025. The copyright holder for this preprint (which was not certified by peer review) is the author/funder, who has granted bioRxiv a license to display the preprint in perpetuity. It is made available under aCC-BY 4.0 International license.

Figure S4. Annotation and differentially-expressed genes in male and female thorax, related to Figure 1.
(A) Photo of dissected female (top) and male (bottom) thoraxes with anatomical diagram of thorax dissection (in orange), and collected sample information. Scale bar: 500 µm.
(B) UMAP of thorax nuclei, colored by sample (female samples = 1, male samples = 1)
(C) UMAP of thorax nuclei clustered using the Leiden algorithm (resolution = 5) for annotation.
(D) UMAP of nuclei from both thorax samples, colored and numbered by manual annotation using selected marker genes as listed in legend at the right of the figure panel. See Table S3 and Table S4 for gene IDs and thresholds.
(E-G) Quantification of differences between male and female samples. Numbered and colored circles above (E) correspond to annotations in (D).
(E) Dot plot of number of differentially expressed genes between females and males in each cell type. Dots represent relative number of differentially expressed genes (DEG) upregulated in male or female with a |log fold change| > 1 and false discovery rate < 0.05, determined by MAST on normalized expression and converted to $\log_2$ fold change.
(F) Sexual abundance difference index from data in (G). Each cell type was categorized based on the following index: Female biased (purple) if 0.3 < abundance index; Neutral (grey) if -0.3 =< abundance index =< 0.3; Male biased (yellow) if abundance index < -0.3.
(G) Bar plot of relative cell abundance, represented as the percent of each cell type of all nuclei collected from each sex for tissue. Separated by female (purple) and male (yellow).

bioRxiv preprint doi: https://doi.org/10.1101/2025.02.25.639765; this version posted February 25, 2025. The copyright holder for this preprint (which was not certified by peer review) is the author/funder, who has granted bioRxiv a license to display the preprint in perpetuity. It is made available under aCC-BY 4.0 International license.

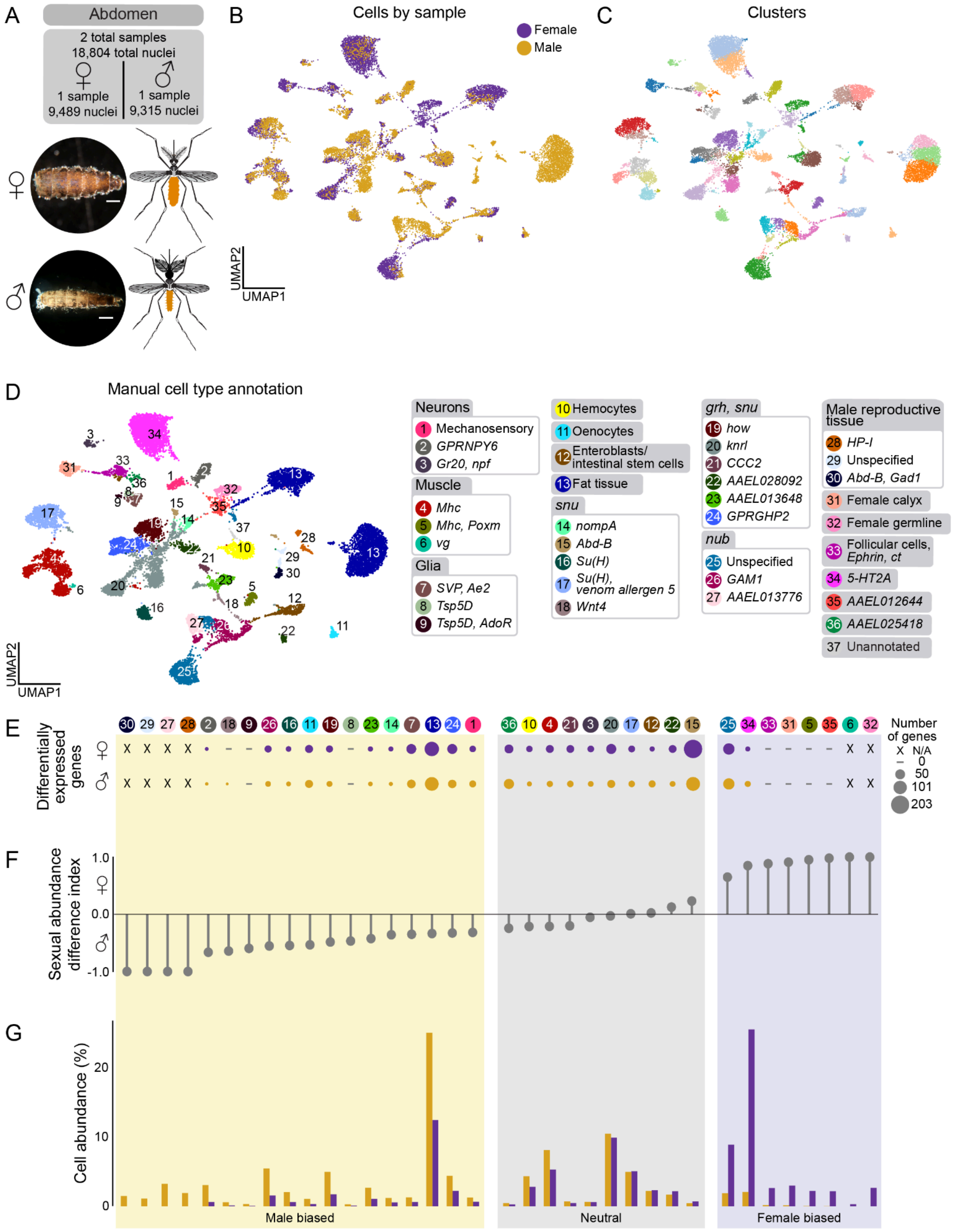

bioRxiv preprint doi: https://doi.org/10.1101/2025.02.25.639765; this version posted February 25, 2025. The copyright holder for this preprint (which was not certified by peer review) is the author/funder, who has granted bioRxiv a license to display the preprint in perpetuity. It is made available under aCC-BY 4.0 International license.

Figure S5. Annotation and differentially-expressed genes in male and female abdomen, related to Figure 1.
(A) Photo of dissected female (top) and male (bottom) abdomens with anatomical diagram of abdomen dissection (in orange), and collected sample information. Scale bar: 500 µm.
(B) UMAP of abdomen nuclei, colored by sample (female samples = 1, male samples = 1)
(C) UMAP of abdomen nuclei clustered using the Leiden algorithm (resolution = 5) for annotation.
(D) UMAP of nuclei from both abdomen samples, colored and numbered by manual annotation using selected marker genes as listed in legend at the right of the figure panel. See Table S3 and Table S4 for gene IDs and thresholds.
(E-G) Quantification of differences between male and female samples. Numbered and colored circles above (E) correspond to annotations in (D).
(E) Dot plot of number of differentially expressed genes between females and males in each cell type. Dots represent relative number of differentially expressed genes (DEG) upregulated in male or female with a |log fold change| > 1 and false discovery rate < 0.05, determined by MAST on normalized expression and converted to $\log_2$ fold change.
(F) Sexual abundance difference index from data in (G). Each cell type was categorized based on the following index: Female biased (purple) if 0.3 < abundance index; Neutral (grey) if -0.3 =< abundance index =< 0.3; Male biased (yellow) if abundance index < -0.3.
(G) Bar plot of relative cell abundance, represented as the percent of each cell type of all nuclei collected from each sex for tissue. Separated by female (purple) and male (yellow).

bioRxiv preprint doi: https://doi.org/10.1101/2025.02.25.639765; this version posted February 25, 2025. The copyright holder for this preprint (which was not certified by peer review) is the author/funder, who has granted bioRxiv a license to display the preprint in perpetuity. It is made available under aCC-BY 4.0 International license.

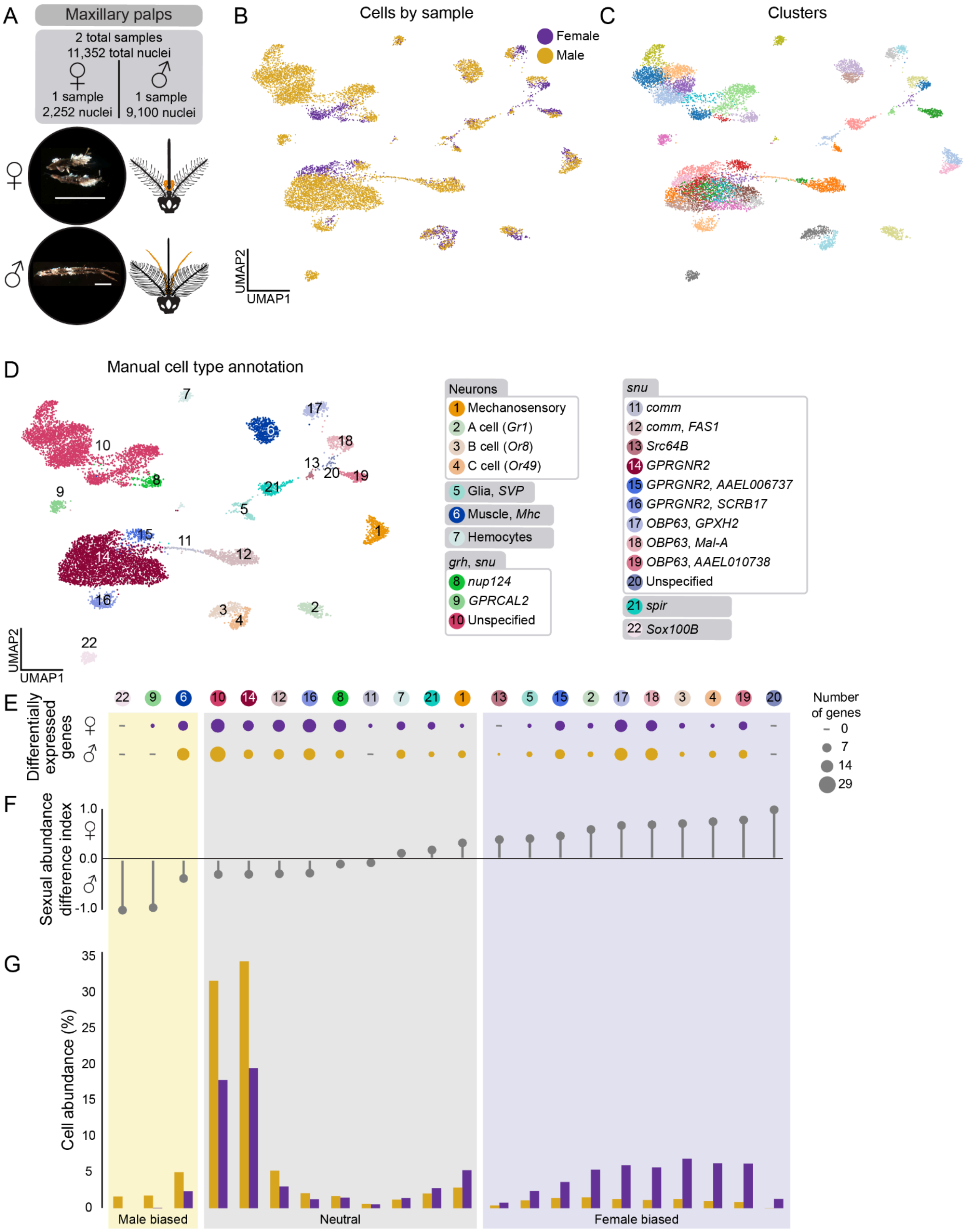

bioRxiv preprint doi: https://doi.org/10.1101/2025.02.25.639765; this version posted February 25, 2025. The copyright holder for this preprint (which was not certified by peer review) is the author/funder, who has granted bioRxiv a license to display the preprint in perpetuity. It is made available under aCC-BY 4.0 International license.

Figure S6. Annotation and differentially-expressed genes in male and female maxillary palps, related to Figure 1.
(A) Photo of dissected female (top) and male (bottom) maxillary palps with anatomical diagram of maxillary palps dissection (in orange), and collected sample information. Scale bar: 500 μm.
(B) UMAP of maxillary palp nuclei, colored by sample (female samples = 1, male samples = 1)
(C) UMAP of maxillary palp nuclei clustered using the Leiden algorithm (resolution = 5) for annotation.
(D) UMAP of nuclei from both maxillary palp samples, colored and numbered by manual annotation using selected marker genes as listed in legend at the right of the figure panel. See Table S3 and Table S4 for gene IDs and thresholds.
(E-G) Quantification of differences between male and female samples. Numbered and colored circles above (E) correspond to annotations in (D).
(E) Dot plot of number of differentially expressed genes between females and males in each cell type. Dots represent relative number of differentially expressed genes (DEG) upregulated in male or female with a |log fold change| > 1 and false discovery rate < 0.05, determined by MAST on normalized expression and converted to $\log_2$ fold change.
(F) Sexual abundance difference index from data in (G). Each cell type was categorized based on the following index: Female biased (purple) if 0.3 < abundance index; Neutral (grey) if -0.3 =< abundance index =< 0.3; Male biased (yellow) if abundance index < -0.3.
(G) Bar plot of relative cell abundance, represented as the percent of each cell type of all nuclei collected from each sex for tissue. Separated by female (purple) and male (yellow).

bioRxiv preprint doi: https://doi.org/10.1101/2025.02.25.639765; this version posted February 25, 2025. The copyright holder for this preprint (which was not certified by peer review) is the author/funder, who has granted bioRxiv a license to display the preprint in perpetuity. It is made available under aCC-BY 4.0 International license.

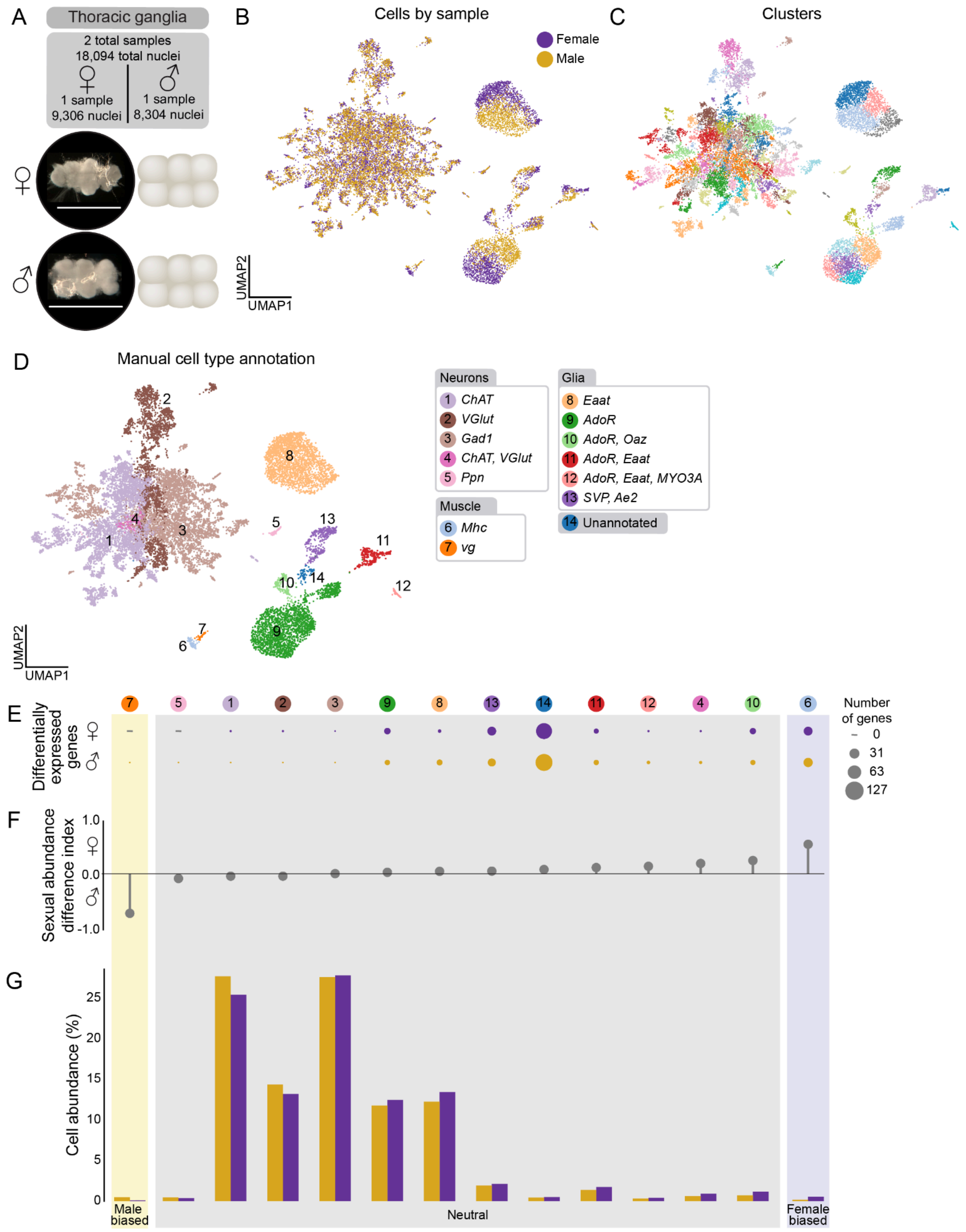

bioRxiv preprint doi: https://doi.org/10.1101/2025.02.25.639765; this version posted February 25, 2025. The copyright holder for this preprint (which was not certified by peer review) is the author/funder, who has granted bioRxiv a license to display the preprint in perpetuity. It is made available under aCC-BY 4.0 International license.

Figure S7. Annotation and differentially-expressed genes in male and female thoracic ganglia, related to Figure 1.
(A) Photo of dissected female (top) and male (bottom) thoracic ganglia with anatomical diagram, and collected sample information. Scale bar: 500 µm.
(B) UMAP of thoracic ganglia nuclei, colored by sample (female samples = 1, male samples = 1)
(C) UMAP of thoracic ganglia nuclei clustered using the Leiden algorithm (resolution = 5) for annotation.
(D) UMAP of nuclei from both thoracic ganglia samples, colored and numbered by manual annotation using selected marker genes as listed in legend at the right of the figure panel. See Table S3 and Table S4 for gene IDs and thresholds.
(E-G) Quantification of differences between male and female samples. Numbered and colored circles above (E) correspond to annotations in (D).
(E) Dot plot of number of differentially expressed genes between females and males in each cell type. Dots represent relative number of differentially expressed genes (DEG) upregulated in male or female with a |log fold change| > 1 and false discovery rate < 0.05, determined by MAST on normalized expression and converted to $\log_2$ fold change.
(F) Sexual abundance difference index from data in (G). Each cell type was categorized based on the following index: Female biased (purple) if 0.3 < abundance index; Neutral (grey) if -0.3 =< abundance index =< 0.3; Male biased (yellow) if abundance index < -0.3.
(G) Bar plot of relative cell abundance, represented as the percent of each cell type of all nuclei collected from each sex for tissue. Separated by female (purple) and male (yellow).

bioRxiv preprint doi: https://doi.org/10.1101/2025.02.25.639765; this version posted February 25, 2025. The copyright holder for this preprint (which was not certified by peer review) is the author/funder, who has granted bioRxiv a license to display the preprint in perpetuity. It is made available under aCC-BY 4.0 International license.

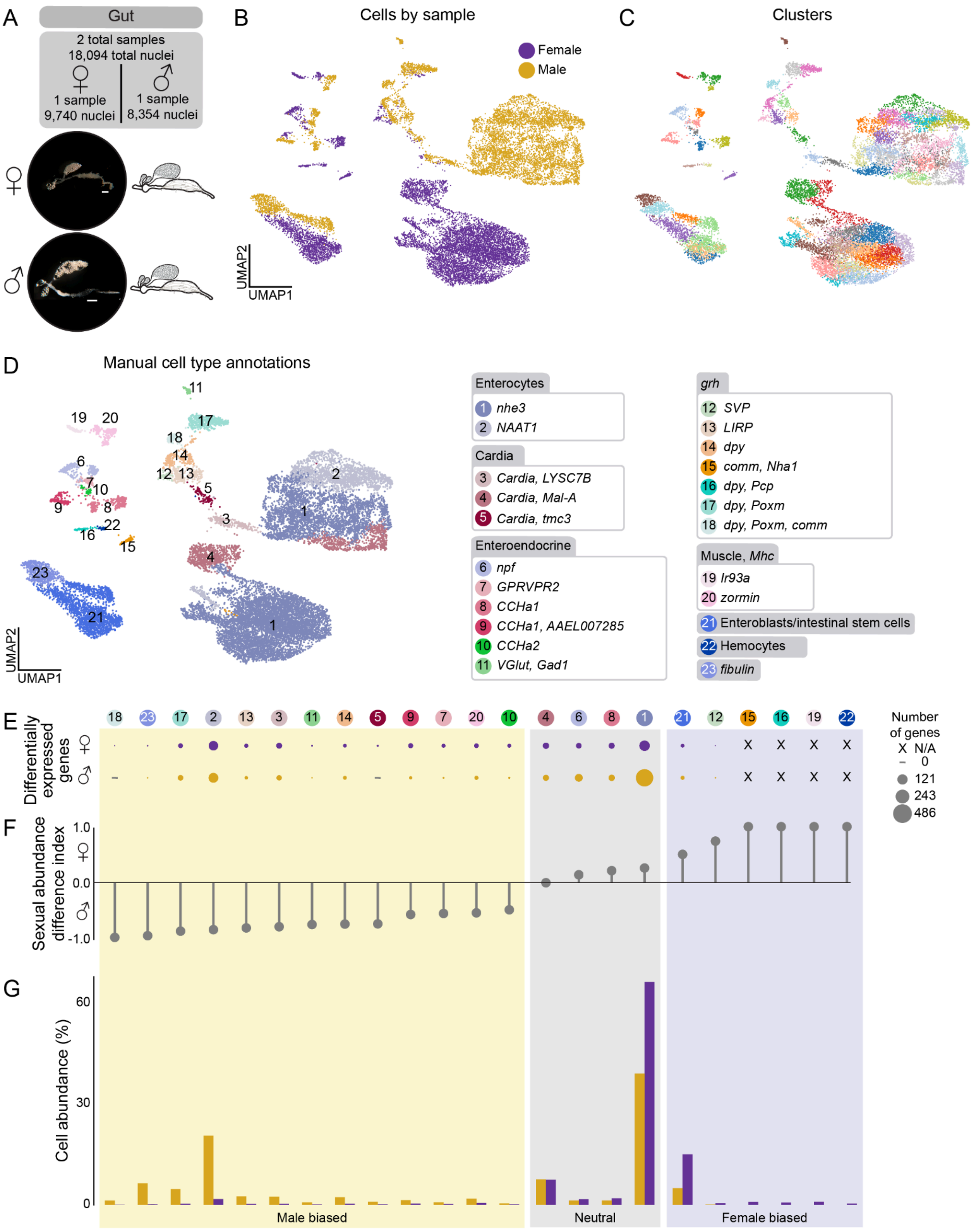

bioRxiv preprint doi: https://doi.org/10.1101/2025.02.25.639765; this version posted February 25, 2025. The copyright holder for this preprint (which was not certified by peer review) is the author/funder, who has granted bioRxiv a license to display the preprint in perpetuity. It is made available under aCC-BY 4.0 International license.

Figure S8. Annotation and differentially-expressed genes in male and female gut, related to Figure 1.
(A) Photo of dissected female (top) and male (bottom) gut with anatomical diagram, and collected sample information. Scale bar: 500 μm.
(B) UMAP of gut nuclei, colored by sample (female samples = 1, male samples = 1)
(C) UMAP of gut nuclei clustered using the Leiden algorithm (resolution = 5) for annotation.
(D) UMAP of nuclei from both gut samples, colored and numbered by manual annotation using selected marker genes as listed in legend at the right of the figure panel. See Table S3 and Table S4 for gene IDs and thresholds.
(E-G) Quantification of differences between male and female samples. Numbered and colored circles above (E) correspond to annotations in (D).
(E) Dot plot of number of differentially expressed genes between females and males in each cell type. Dots represent relative number of differentially expressed genes (DEG) upregulated in male or female with a |log fold change| > 1 and false discovery rate < 0.05, determined by MAST on normalized expression and converted to $\log_2$ fold change.
(F) Sexual abundance difference index from data in (G). Each cell type was categorized based on the following index: Female biased (purple) if 0.3 < abundance index; Neutral (grey) if -0.3 =< abundance index =< 0.3; Male biased (yellow) if abundance index < -0.3.
(G) Bar plot of relative cell abundance, represented as the percent of each cell type of all nuclei collected from each sex for tissue. Separated by female (purple) and male (yellow).

bioRxiv preprint doi: https://doi.org/10.1101/2025.02.25.639765; this version posted February 25, 2025. The copyright holder for this preprint (which was not certified by peer review) is the author/funder, who has granted bioRxiv a license to display the preprint in perpetuity. It is made available under aCC-BY 4.0 International license.

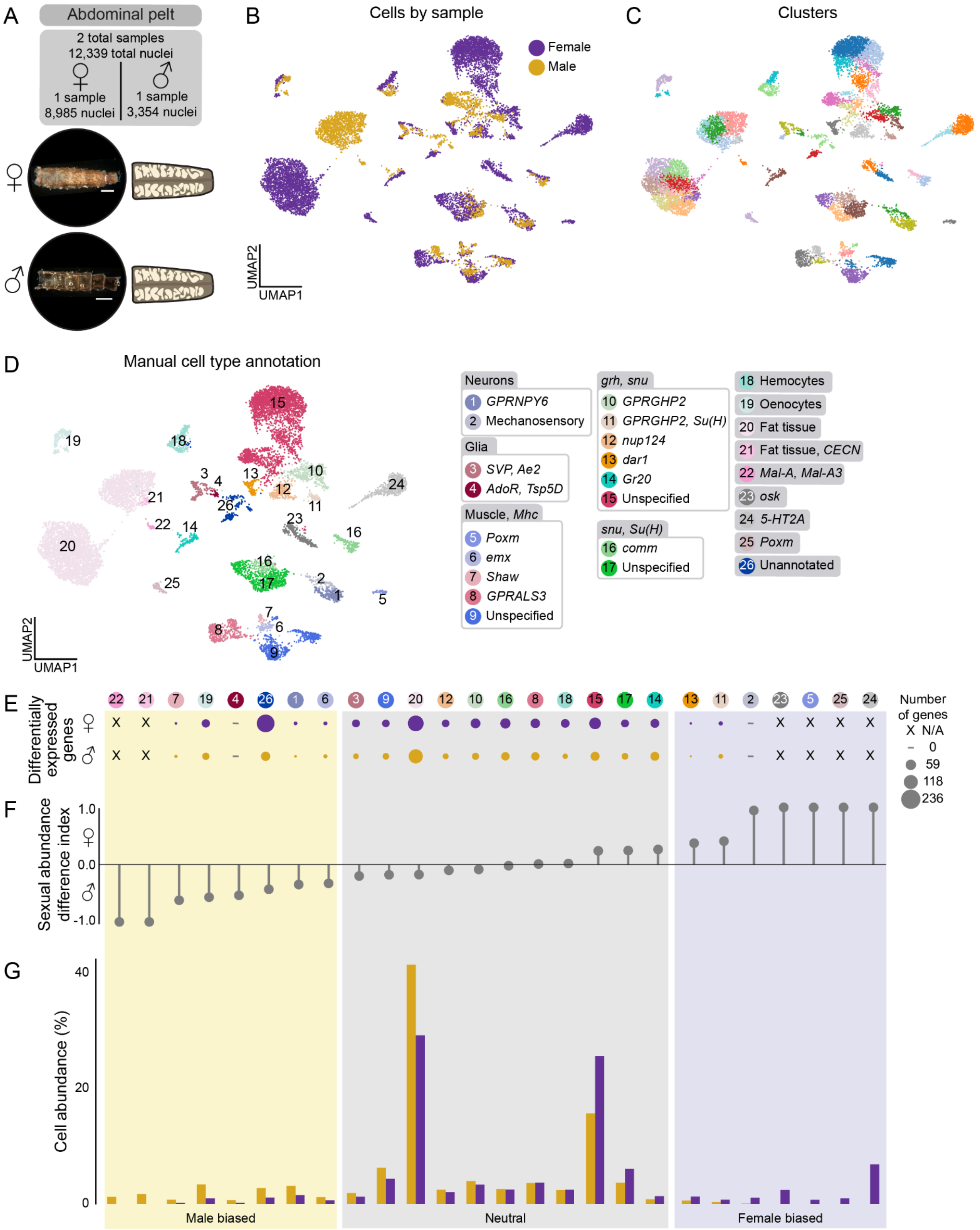

bioRxiv preprint doi: https://doi.org/10.1101/2025.02.25.639765; this version posted February 25, 2025. The copyright holder for this preprint (which was not certified by peer review) is the author/funder, who has granted bioRxiv a license to display the preprint in perpetuity. It is made available under aCC-BY 4.0 International license.

Figure S9. Annotation and differentially-expressed genes in male and female abdominal pelt, related to Figure 1.
(A) Photo of dissected female (top) and male (bottom) abdominal pelt with anatomical diagram, and collected sample information. Scale bar: 500 μm.
(B) UMAP of abdominal pelt nuclei, colored by sample (female samples = 1, male samples = 1)
(C) UMAP of abdominal pelt nuclei clustered using the Leiden algorithm (resolution = 5) for annotation.
(D) UMAP of nuclei from both abdominal pelt samples, colored and numbered by manual annotation using selected marker genes as listed in legend at the right of the figure panel. See Table S3 and Table S4 for gene IDs and thresholds.
(E-G) Quantification of differences between male and female samples. Numbered and colored circles above (E) correspond to annotations in (D).
(E) Dot plot of number of differentially expressed genes between females and males in each cell type. Dots represent relative number of differentially expressed genes (DEG) upregulated in male or female with a |log fold change| > 1 and false discovery rate < 0.05, determined by MAST on normalized expression and converted to $\log_2$ fold change.
(F) Sexual abundance difference index from data in (G). Each cell type was categorized based on the following index: Female biased (purple) if 0.3 < abundance index; Neutral (grey) if -0.3 =< abundance index =< 0.3; Male biased (yellow) if abundance index < -0.3.
(G) Bar plot of relative cell abundance, represented as the percent of each cell type of all nuclei collected from each sex for tissue. Separated by female (purple) and male (yellow).

bioRxiv preprint doi: https://doi.org/10.1101/2025.02.25.639765; this version posted February 25, 2025. The copyright holder for this preprint (which was not certified by peer review) is the author/funder, who has granted bioRxiv a license to display the preprint in perpetuity. It is made available under aCC-BY 4.0 International license.

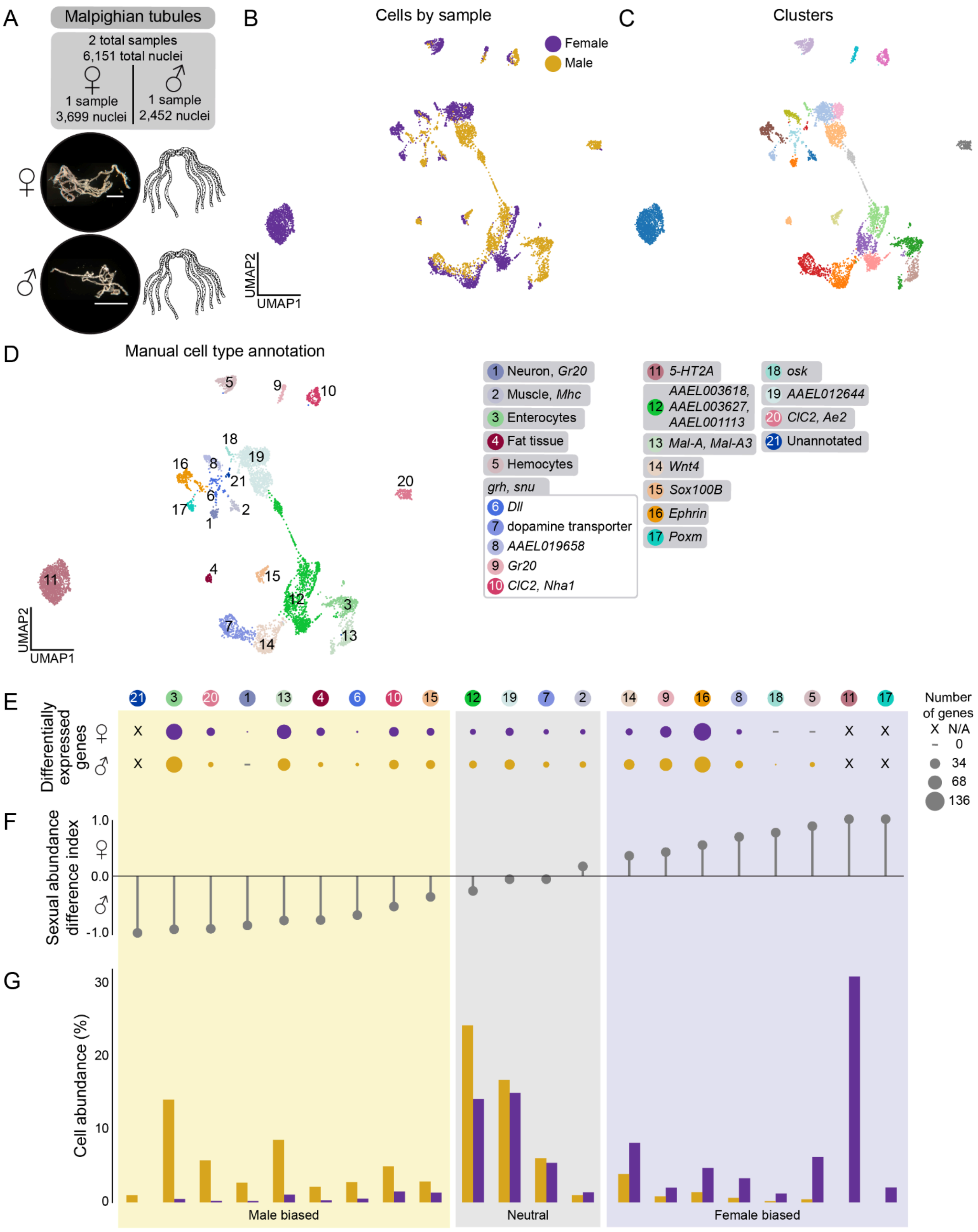

bioRxiv preprint doi: https://doi.org/10.1101/2025.02.25.639765; this version posted February 25, 2025. The copyright holder for this preprint (which was not certified by peer review) is the author/funder, who has granted bioRxiv a license to display the preprint in perpetuity. It is made available under aCC-BY 4.0 International license.

Figure S10. Annotation and differentially-expressed genes in male and female malpighian tubules, related to Figure 1.
(A) Photo of dissected female (top) and male (bottom) malpighian tubules with anatomical diagram, and collected sample information. Scale bar: 500 µm.
(B) UMAP of malpighian tubules nuclei, colored by sample (female samples = 1, male samples = 1)
(C) UMAP of malpighian tubules nuclei clustered using the Leiden algorithm (resolution = 5) for annotation.
(D) UMAP of nuclei from both malpighian tubules samples, colored and numbered by manual annotation using selected marker genes as listed in legend at the right of the figure panel. See Table S3 and Table S4 for gene IDs and thresholds.
(E-G) Quantification of differences between male and female samples. Numbered and colored circles above (E) correspond to annotations in (D).
(E) Dot plot of number of differentially expressed genes between females and males in each cell type. Dots represent relative number of differentially expressed genes (DEG) upregulated in male or female with a |log fold change| > 1 and false discovery rate < 0.05, determined by MAST on normalized expression and converted to $\log_2$ fold change.
(F) Sexual abundance difference index from data in (G). Each cell type was categorized based on the following index: Female biased (purple) if 0.3 < abundance index; Neutral (grey) if -0.3 =< abundance index =< 0.3; Male biased (yellow) if abundance index < -0.3.
(G) Bar plot of relative cell abundance, represented as the percent of each cell type of all nuclei collected from each sex for tissue. Separated by female (purple) and male (yellow).

bioRxiv preprint doi: https://doi.org/10.1101/2025.02.25.639765; this version posted February 25, 2025. The copyright holder for this preprint (which was not certified by peer review) is the author/funder, who has granted bioRxiv a license to display the preprint in perpetuity. It is made available under aCC-BY 4.0 International license.

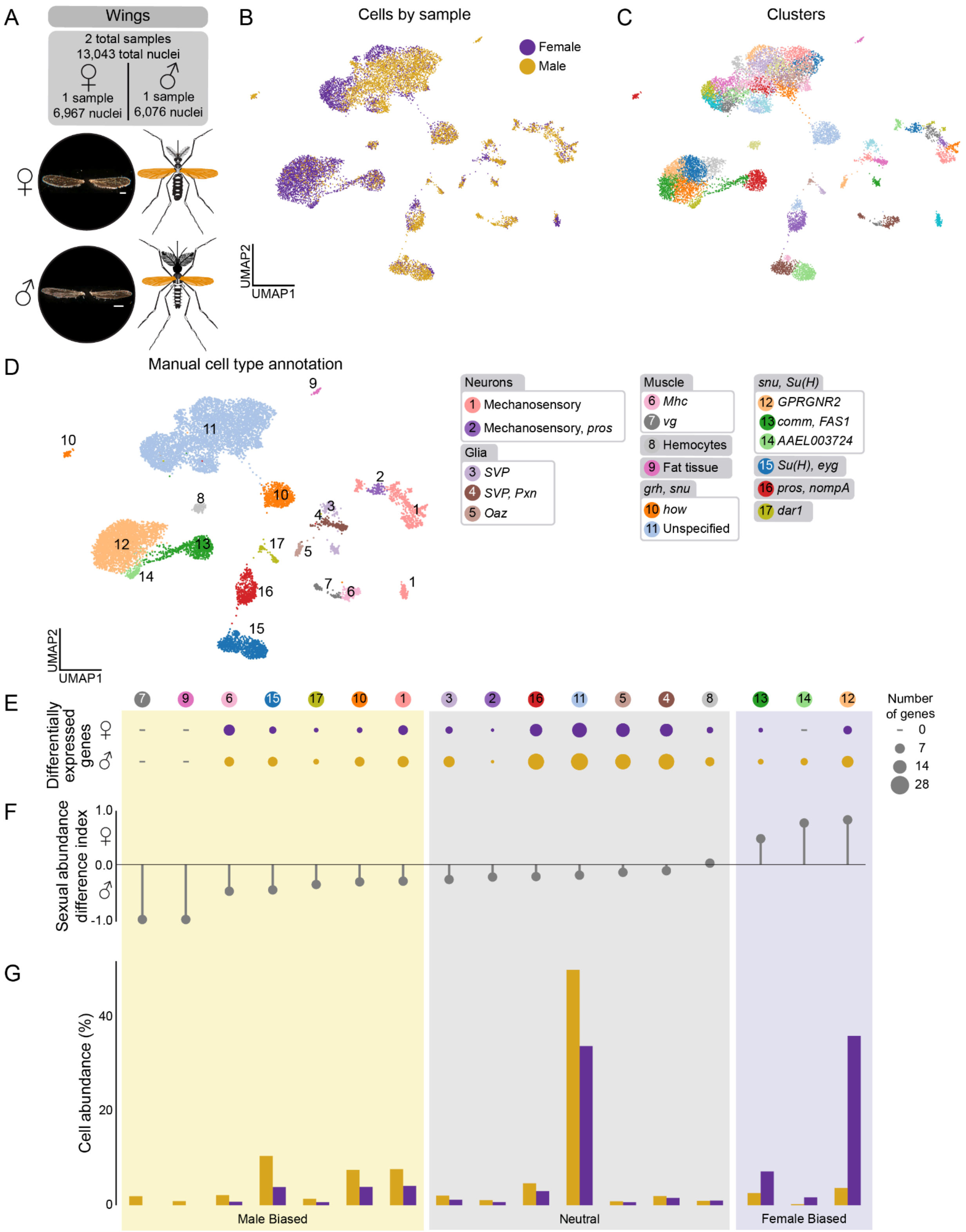

bioRxiv preprint doi: https://doi.org/10.1101/2025.02.25.639765; this version posted February 25, 2025. The copyright holder for this preprint (which was not certified by peer review) is the author/funder, who has granted bioRxiv a license to display the preprint in perpetuity. It is made available under aCC-BY 4.0 International license.

Figure S11. Annotation and differentially-expressed genes in male and female wings, related to Figure 1.
(A) Photo of dissected female (top) and male (bottom) wings with anatomical diagram of wings dissection (in orange), and collected sample information. Scale bar: 500 µm.
(B) UMAP of wing nuclei, colored by sample (female samples = 1, male samples = 1)
(C) UMAP of wing nuclei clustered using the Leiden algorithm (resolution = 5) for annotation.
(D) UMAP of nuclei from both wing samples, colored and numbered by manual annotation using selected marker genes as listed in legend at the right of the figure panel. See Table S3 and Table S4 for gene IDs and thresholds.
(E-G) Quantification of differences between male and female samples. Numbered and colored circles above (E) correspond to annotations in (D).
(E) Dot plot of number of differentially expressed genes between females and males in each cell type. Dots represent relative number of differentially expressed genes (DEG) upregulated in male or female with a |log fold change| > 1 and false discovery rate < 0.05, determined by MAST on normalized expression and converted to $\log_2$ fold change.
(F) Sexual abundance difference index from data in (G). Each cell type was categorized based on the following index: Female biased (purple) if 0.3 < abundance index; Neutral (grey) if -0.3 =< abundance index =< 0.3; Male biased (yellow) if abundance index < -0.3.
(G) Bar plot of relative cell abundance, represented as the percent of each cell type of all nuclei collected from each sex for tissue. Separated by female (purple) and male (yellow).

bioRxiv preprint doi: https://doi.org/10.1101/2025.02.25.639765; this version posted February 25, 2025. The copyright holder for this preprint (which was not certified by peer review) is the author/funder, who has granted bioRxiv a license to display the preprint in perpetuity. It is made available under aCC-BY 4.0 International license.

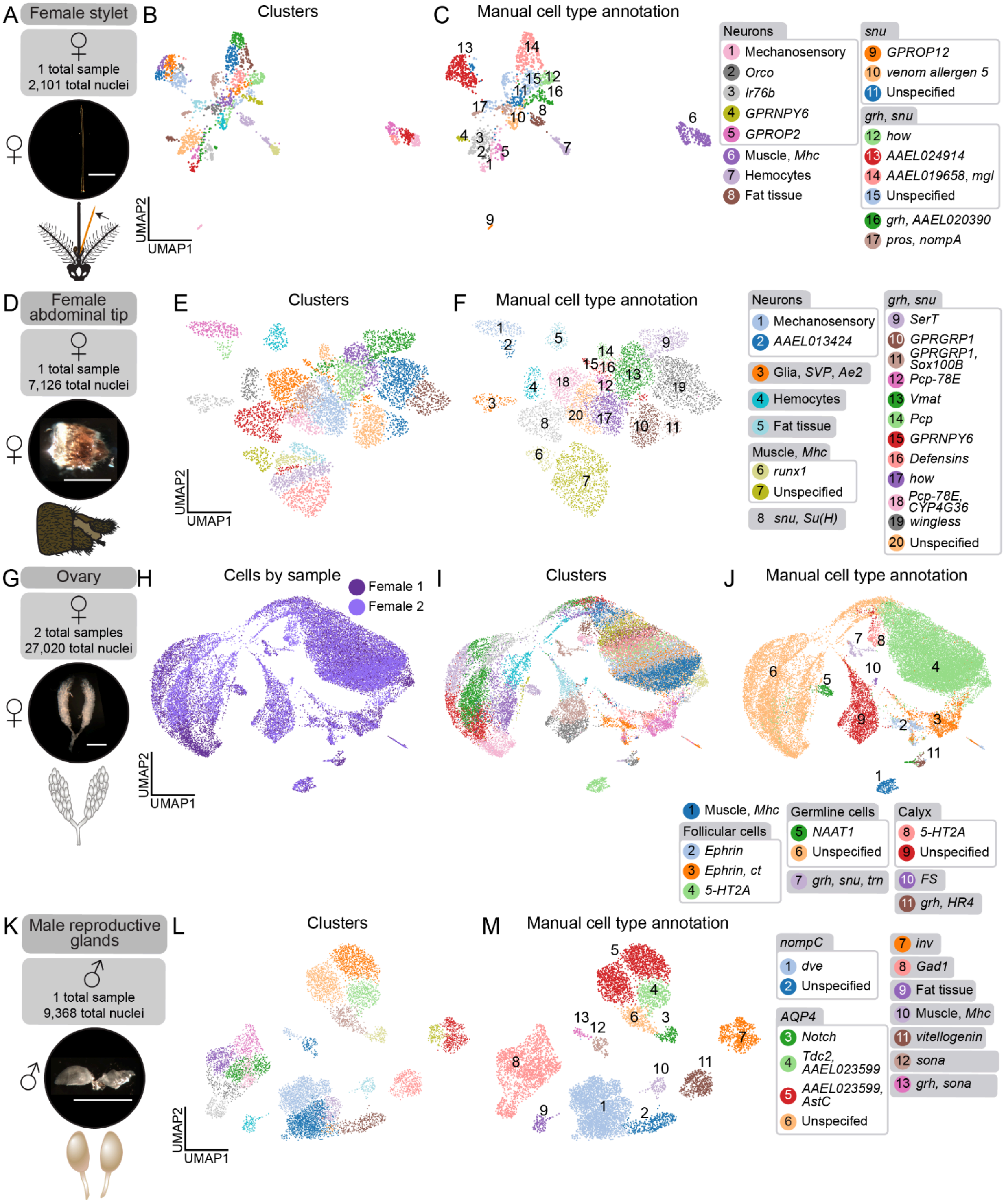

bioRxiv preprint doi: https://doi.org/10.1101/2025.02.25.639765; this version posted February 25, 2025. The copyright holder for this preprint (which was not certified by peer review) is the author/funder, who has granted bioRxiv a license to display the preprint in perpetuity. It is made available under aCC-BY 4.0 International license.

Figure S12. Annotation of female stylet, female abdominal tip, ovaries and male accessory gland, related to Figure 1.
(A) Photo of dissected female stylet with anatomical diagram of stylet dissection (in orange), and collected sample information. Scale bar: 500 µm.
(B) UMAP of female stylet nuclei clustered using the Leiden algorithm (resolution = 5) for annotation.
(C) UMAP of nuclei from female stylet sample, colored and numbered by manual annotation using selected marker genes as listed in legend at the right of the figure panel. See Table S3 and Table S4 for gene IDs and thresholds.
(D) Photo of dissected female abdominal tip with anatomical diagram, and collected sample information. Scale bar: 500 µm.
(E) UMAP of female abdominal tip nuclei clustered using the Leiden algorithm (resolution = 5) for annotation.
(F) UMAP of nuclei from female abdominal tip sample, colored and numbered by manual annotation using selected marker genes as listed in legend at the right of the figure panel. See Table S3 and Table S4 for gene IDs and thresholds.
(G) Photo of dissected female ovary with anatomical diagram, and collected sample information. Scale bar: 500 µm.
(H) UMAP of ovary nuclei, colored by sample (female samples = 2)
(I) UMAP of ovary nuclei clustered using the Leiden algorithm (resolution = 5) for annotation.
(J) UMAP of nuclei from ovary samples, colored and numbered by manual annotation using selected marker genes as listed in legend at the bottom of figure panel. See Table S3 and Table S4 for gene IDs and thresholds.
(K) Photo of dissected male reproductive glands with anatomical diagram, and collected sample information. Scale bar: 500 µm.
(L) UMAP of male reproductive gland nuclei clustered using the Leiden algorithm (resolution = 5) for annotation.
(M) UMAP of nuclei from male reproductive gland sample, colored and numbered by manual annotation using selected marker genes as listed in legend at the right of the figure panel. See Table S3 and Table S4 for gene IDs and thresholds.

bioRxiv preprint doi: https://doi.org/10.1101/2025.02.25.639765; this version posted February 25, 2025. The copyright holder for this preprint (which was not certified by peer review) is the author/funder, who has granted bioRxiv a license to display the preprint in perpetuity. It is made available under aCC-BY 4.0 International license.

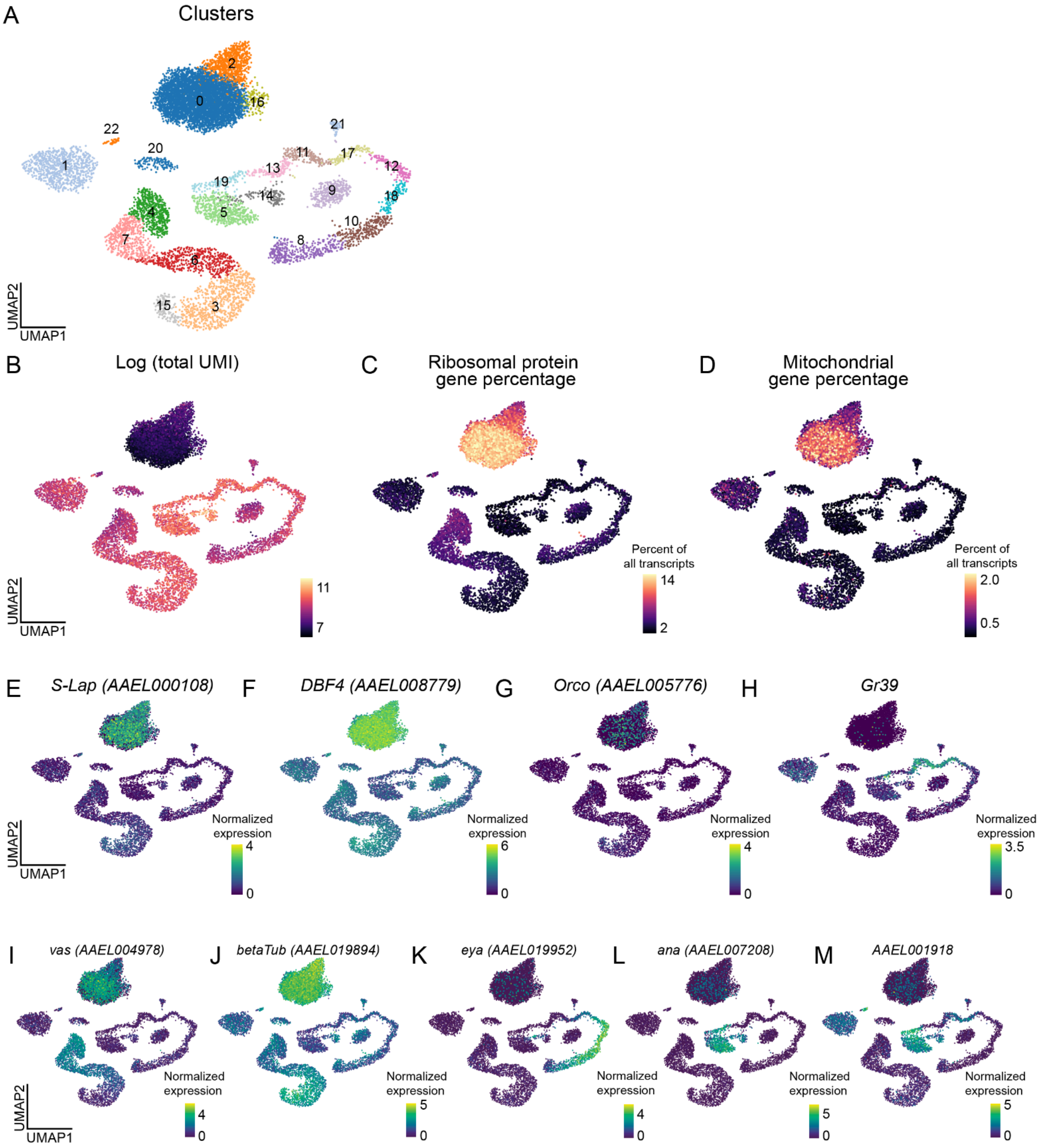

Figure S13. Localization of spermatid RNA transcripts in testes data, related to Figure 2.

(A) UMAP of all testes cells after quality control and filtering (Louvain algorithm, resolution = 1).

(B) UMAP of testes cells colored by log total UMI for each cell.

(C-D) UMAP of testes sample colored by percent of all transcripts in each cell for expression of ribosomal protein genes (C), expression of mitochondrial genes (D). Color bars trimmed 0.5% for visibility.

bioRxiv preprint doi: https://doi.org/10.1101/2025.02.25.639765; this version posted February 25, 2025. The copyright holder for this preprint (which was not certified by peer review) is the author/funder, who has granted bioRxiv a license to display the preprint in perpetuity. It is made available under aCC-BY 4.0 International license.

(E-H) UMAPs of testes normalized expression of *S-Lap* (*AAEL000108*) (E), *DBF4* (*AAEL008779*) (F), *Orco* (*AAEL005776*) (G), and *Gr39* (H).Normalized expression is ln([(raw count/total cell counts)x median total counts across cells]+1).
(I-M) UMAP of normalized gene expression of a subset of genes used to annotate testes data that were used in Figure 2C-G. Genes include include *vas* (*AAEL004978*) (I), *betaTub* (*AAEL019894*) (J), *eya (AAEL019952*) (K), *ana (AAEL007208)* (L), and *AAEL001918* (M). Note normalized signal of genes expressed in spermatids appears high relative to other cell types in normalized expression due to their overall low transcript count (Figure S13B).

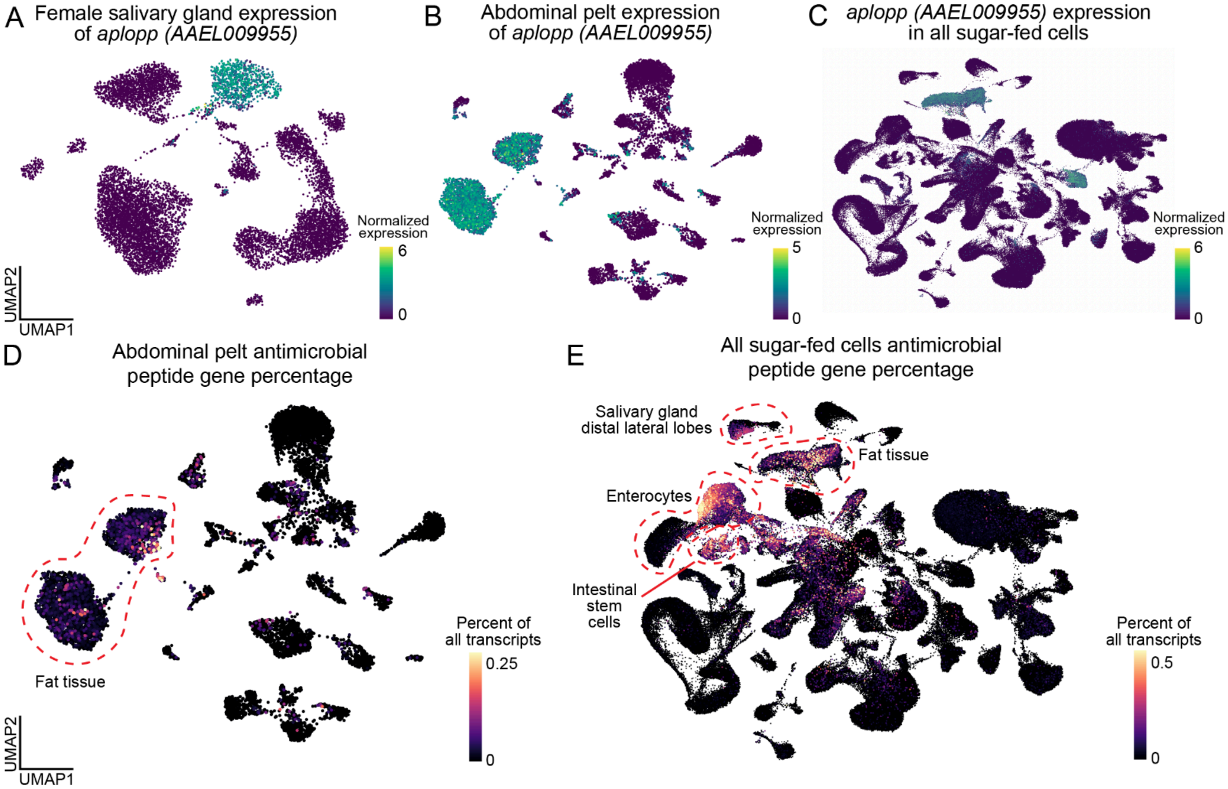

Figure S14. Antimicrobial genes in fat tissue, related to Figure 3.
(A-C) UMAPs of normalized expression of marker gene for fat tissue *aplopp (AAEL009955)* in the salivary gland (A), abdominal pelt (B), and all sugar-fed nuclei (C). Normalized expression is ln([(raw count/total cell counts)x median total counts across cells]+1).
(D-E) UMAP, colored by expression of antimicrobial peptides gene set (Table S2) for abdominal pelt (D) and all sugar-fed nuclei (E). Color represents fraction of total transcripts in each cell (color bar trimmed 0.1% for visibility). Relevant cell types labeled.

bioRxiv preprint doi: https://doi.org/10.1101/2025.02.25.639765; this version posted February 25, 2025. The copyright holder for this preprint (which was not certified by peer review) is the author/funder, who has granted bioRxiv a license to display the preprint in perpetuity. It is made available under aCC-BY 4.0 International license.

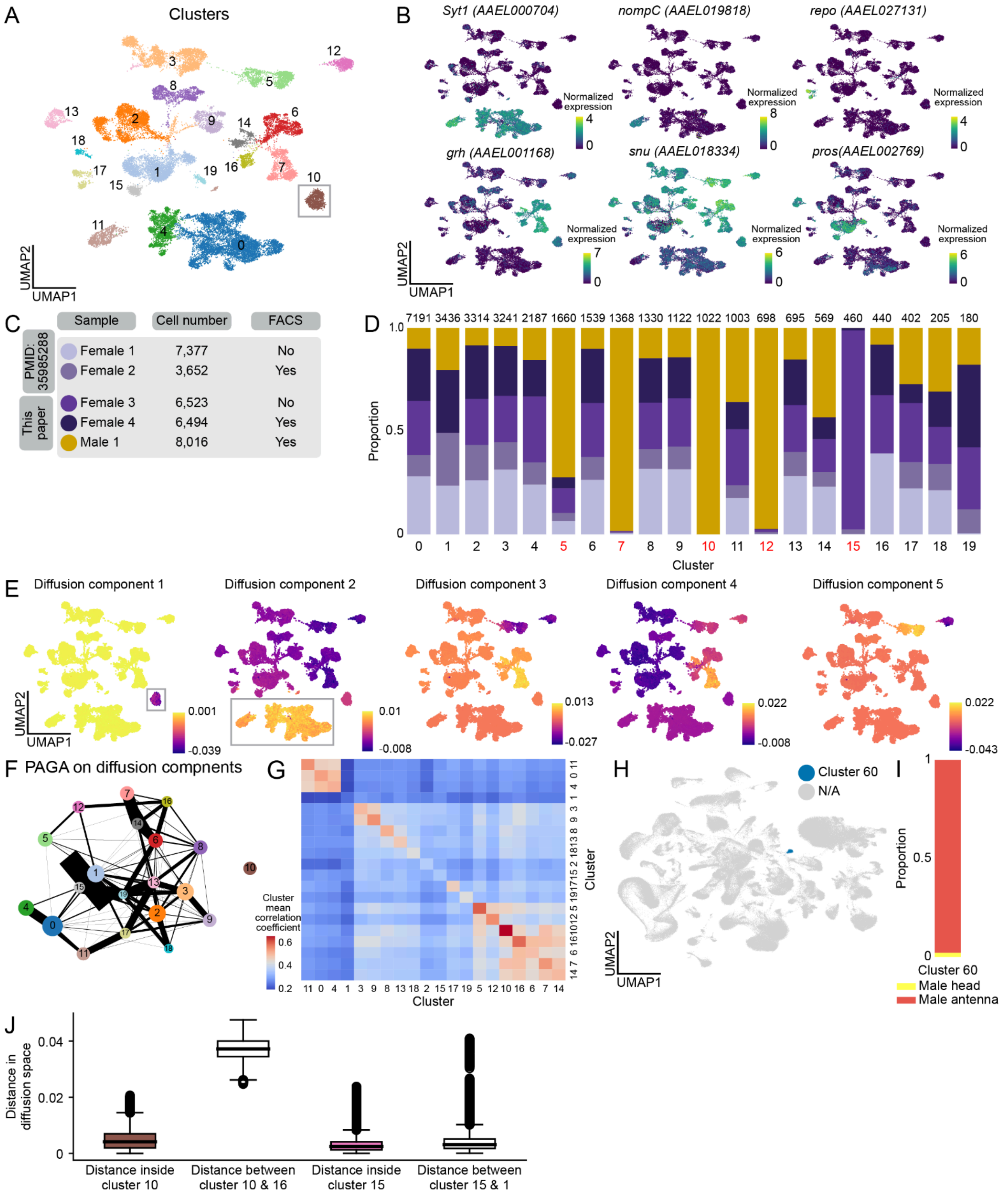

bioRxiv preprint doi: https://doi.org/10.1101/2025.02.25.639765; this version posted February 25, 2025. The copyright holder for this preprint (which was not certified by peer review) is the author/funder, who has granted bioRxiv a license to display the preprint in perpetuity. It is made available under aCC-BY 4.0 International license.

Figure S15. Identification of male-specific ppk317 cell type in Aedes aegypti antenna, related to Figure 4.
(A) UMAP of antenna nuclei clustered and numbered using the Leiden algorithm (resolution = 0.1). Cluster 10 (male-specific, *ppk317*-expressing cells) highlighted in gray.
(B) UMAPs of all antenna nuclei colored by normalized expression of *Syt1 (AAEL000704), nompC (AAEL019818), repo (AAEL027131), grh (AAEL001168), snu (AAEL018334),* and *pros (AAEL002769)*. Normalized expression is ln([(raw count/total cell counts)x median total counts across cells]+1).
(C) Number of cells in each sample (female = 4, male = 1), source, and if each sample underwent fluorescence-activated cell sorting.
(D) Bar plot of proportion that each sample makes up each cluster based on numbering from (A). Cluster numbers below bar plot. Clusters for which over 70% originate form a single sample indicated in red. Numbers above each bar indicate the number of cells in that cluster.
(E) UMAPs of diffusion components 1-5. Diffusion component 1 (first panel) maps to cluster 10 (*ppk317*-expressing cells, circled in red), suggesting a robust biological feature. Diffusion component 2 (second panel) maps to neurons, highlighted in gray.
(F) Partition-based graph abstraction (PAGA) calculated on diffusion components in (E). No edge threshold set.
(G) Correlation matrix heatmap, depicting pairwise correlation of gene expression matrices between each cluster (mean Pearson correlation coefficient). Diagonal values (cluster to itself) represents intra-cluster correlation values, which vary based on features such as cell number and gene heterogeneity.
(H) UMAP of integrated Mosquito Cell Atlas data. Antennal cells expressing *ppk317* belong to cluster 60 (Louvain algorithm, resolution = 0.1).
(I) Stacked bar plot of origin of cells from cluster 60. Cluster 60 (all *ppk317*-expressing cells in the male and female mosquito) come from the male antenna and male head sample.
(J) Pairwise Euclidean distances on diffusion embeddings from (E) to approximate phenotypic distance between and within clusters. Boxes represent first quartile to third quartile, and middle line represents median. Whiskers represent 1.5x interquartile range, with data points outside this range as outliers.

bioRxiv preprint doi: https://doi.org/10.1101/2025.02.25.639765; this version posted February 25, 2025. The copyright holder for this preprint (which was not certified by peer review) is the author/funder, who has granted bioRxiv a license to display the preprint in perpetuity. It is made available under aCC-BY 4.0 International license.

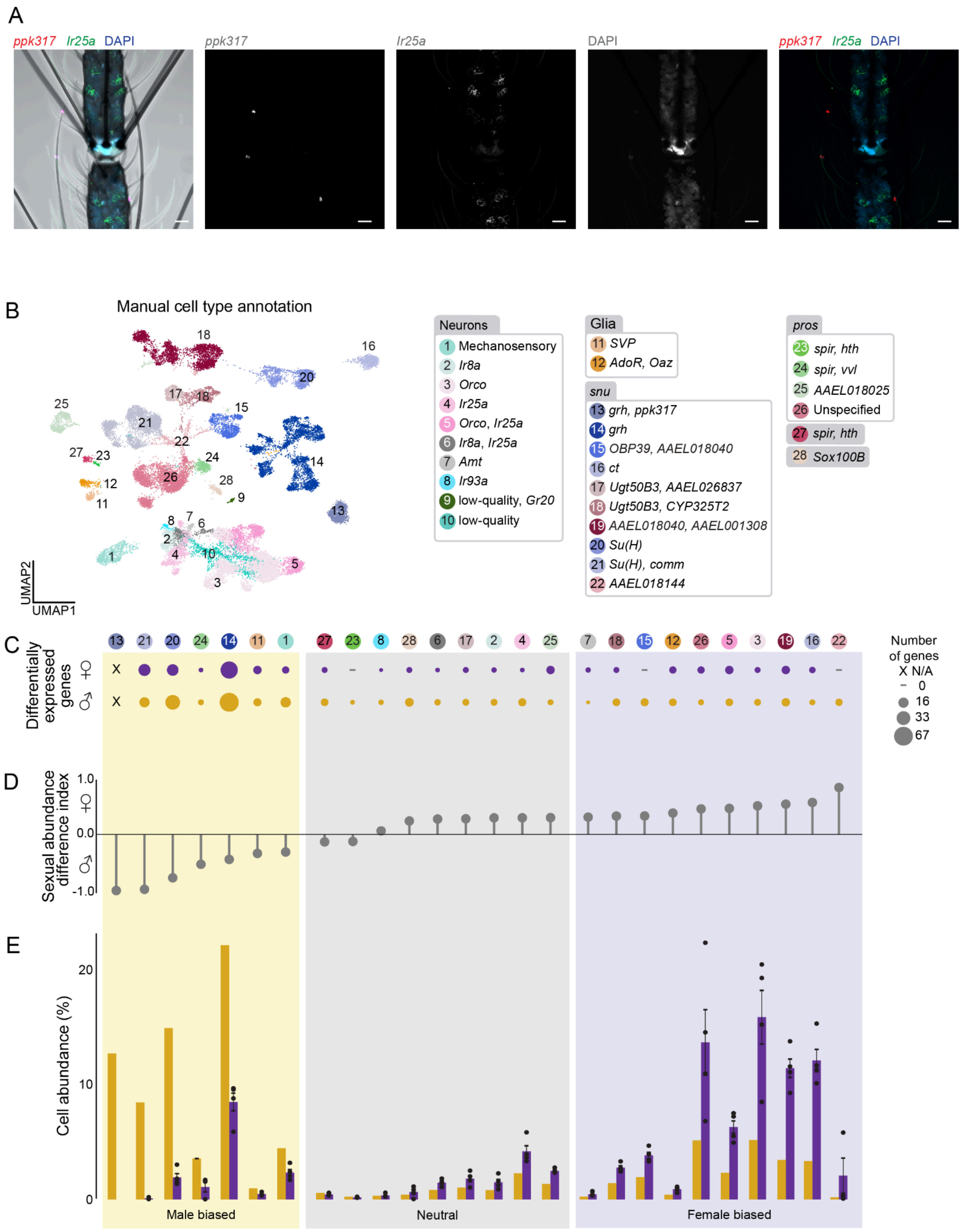

bioRxiv preprint doi: https://doi.org/10.1101/2025.02.25.639765; this version posted February 25, 2025. The copyright holder for this preprint (which was not certified by peer review) is the author/funder, who has granted bioRxiv a license to display the preprint in perpetuity. It is made available under aCC-BY 4.0 International license.

Figure S16. Antennae samples and annotation, related to Figures 1 and 4.
(A) Maximum-intensity projection of whole-mount female antennae with RNA *in situ* hybridization of *ppk317* probe (magenta), *Ir25a* (green) and nuclear staining (DAPI). Scale bar: 10 μm.
(B) UMAP of antenna nuclei clustered using the Leiden algorithm (resolution = 5).
(C) UMAP of nuclei from all antennae samples, colored and numbered by manual annotation using selected marker genes as listed in legend at the right of the figure panel. See Table S3 and Table S4 for gene IDs and thresholds.
(C-E) Quantification of differences between male and female samples. Numbered and colored circles above (C) correspond to annotations in (B).
(C) Dot plot of number of differentially expressed genes between females and males in each cell type. Dots represent relative number of differentially expressed genes (DEG) upregulated in male or female with a |log fold change| > 1 and false discovery rate < 0.05, determined by MAST on normalized expression and converted to $\log_2$ fold change.
(D) Sexual abundance difference index from data in (G). Each cell type was categorized based on the following index: Female biased (purple) if 0.3 < abundance index; Neutral (grey) if -0.3 =< abundance index =< 0.3; Male biased (yellow) if abundance index < -0.3.
(E) Bar plot of relative cell abundance, represented as the percent of each cell type of all nuclei collected from each sex for tissue. Separated by female (purple) and male (yellow). Values for each replicate shown as dots, and standard error indicated by black brackets.

bioRxiv preprint doi: https://doi.org/10.1101/2025.02.25.639765; this version posted February 25, 2025. The copyright holder for this preprint (which was not certified by peer review) is the author/funder, who has granted bioRxiv a license to display the preprint in perpetuity. It is made available under aCC-BY 4.0 International license.

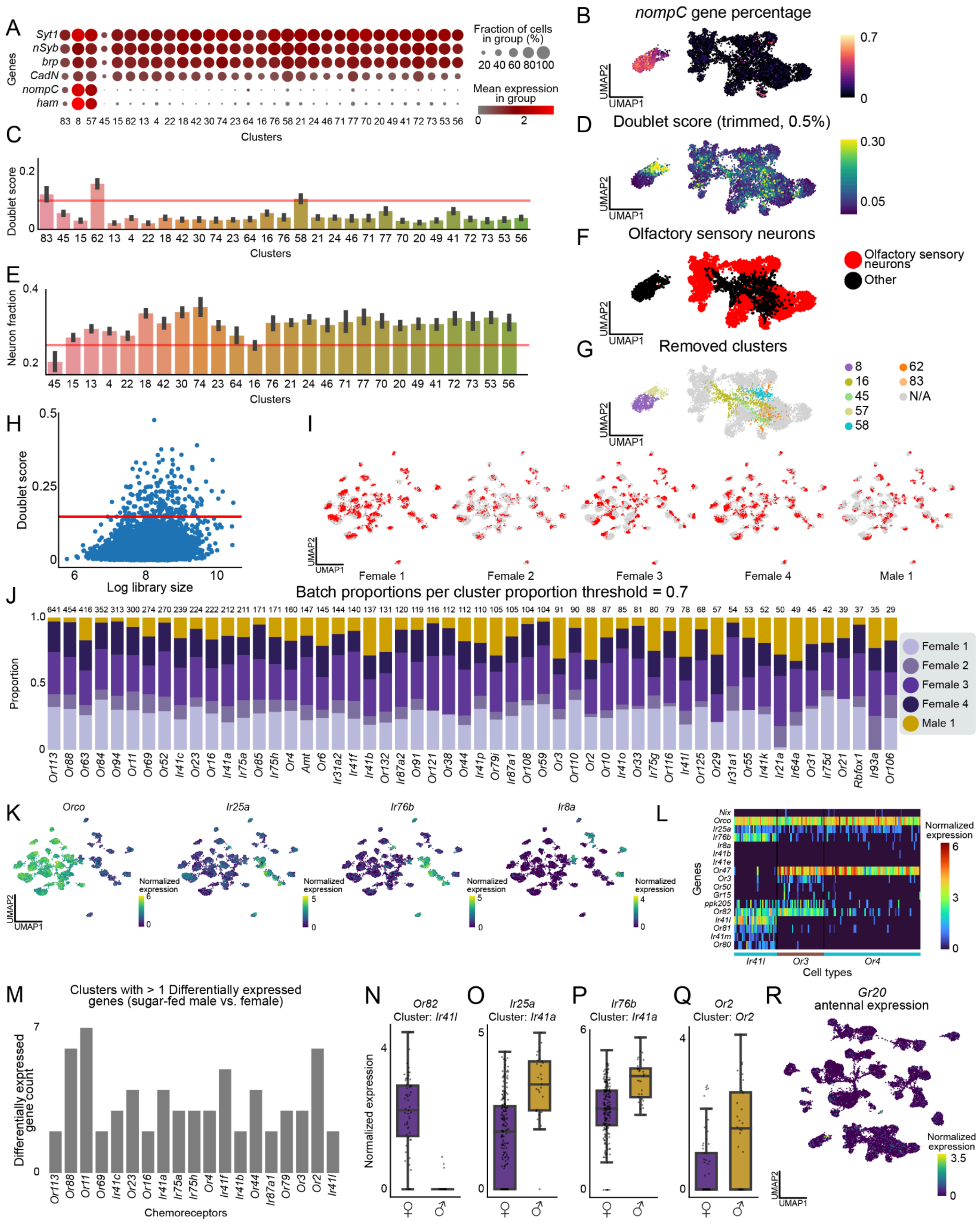

bioRxiv preprint doi: https://doi.org/10.1101/2025.02.25.639765; this version posted February 25, 2025. The copyright holder for this preprint (which was not certified by peer review) is the author/funder, who has granted bioRxiv a license to display the preprint in perpetuity. It is made available under aCC-BY 4.0 International license.

Figure S17. Antenna chemosensory cell type expressing *Orco* and *Ir25a* is sexually dimorphic for *Or82,* related to Figure 5.

(A) Dotplot of neuron marker genes (*Syt1 (AAEL000704), nSyb (AAEL024921), brp (AAEL018153), CadN (AAEL000597), nompC (AAEL019818),* and *ham (AAEL017229)*). Size of dot indicated the percent of cells in each group, color indicated mean expression, see legend in the lower right of the panel.

(B) UMAP of neurons from antenna samples with expression of *nompC* as percent of all transcripts in each cell. UMAPs for (B,D,F,G) in this figure UMAP coordinates of neurons from Figure 5A.

(C) Mean doublet score across cells, with error bars indicating 95% confidence interval calculated from bootstrapping. Generated through scrublet[189]. Clusters with an average score above 0.15 were removed from further analysis (red line).

(D) UMAP of neurons from antenna samples depicting doublet score.

(E) Average percentage of neuronal genes in (A), with error bars indicating 95% confidence interval calculated from bootstrapping. Clusters with an average score below 0.25 were removed from further analysis (red line).

(F) UMAP of neurons from antenna samples, demonstrating which clusters (Leiden, resolution = 5) were kept for downstream analysis (red) or removed based on filtering parameters (black).

(G) UMAP of neurons from antenna samples, demonstrating clusters removed from downstream analysis. UMAP cropped for space, cluster 83 not shown.

(H) Log(library size) versus calculated doublet score for neurons filtered in (A-G). Cells with a score above 0.15 were removed from further analysis (red line).

(I) UMAPs of antenna olfactory sensory neurons (filtered *nompC*-negative sensory neuron population), colored by sample.

(J) Proportion plot for annotated *nompC*-negative sensory neuron population. Bar plot of proportion that each sample makes up each cell type. Numbers above each bar indicate the number of cells in that cluster.

(K) UMAP of normalized gene expression of olfactory co-receptor genes *Orco, Ir25a, Ir76b,* and *Ir8a*. Normalized expression is ln([(raw count/total cell counts)x median total counts across cells]+1).

(L) Heatmap of cells from female samples 3 and 4 (see Figure S15C) from annotated clusters *Ir41l*, *Or3*, and *Or4*. Selected genes are indicated in rows and cells indicated in columns. Cell types indicated below heatmap. Heatmap colors represent normalized expression.

(M) Bar plot of number of differentially expressed genes between females and males in each olfactory sensory neuron cell type. Clusters shown contain at least 1 differentially expressed genes (DEG) with a |log fold change| > 1 and false discovery rate < 0.05 (determined by MAST on normalized expression).

(N-Q) Boxplot illustrating distribution of gene expression of differentially expressed olfactory receptor genes across male and female cells in a particular cluster. *Or82* expression in cluster *Ir41l* (N), *Ir25a* in cluster *Ir41a* (O), *Ir76b* in cluster *Ir41a* (P), *Or2* expression in cluster *Or2* (Q). Boxes represent first quartile to third quartile, and middle line represents median. Whiskers represent 1.5x interquartile range, with data points outside this range as outliers.

(R) UMAP of normalized gene expression of *Gr20*.

bioRxiv preprint doi: https://doi.org/10.1101/2025.02.25.639765; this version posted February 25, 2025. The copyright holder for this preprint (which was not certified by peer review) is the author/funder, who has granted bioRxiv a license to display the preprint in perpetuity. It is made available under aCC-BY 4.0 International license.

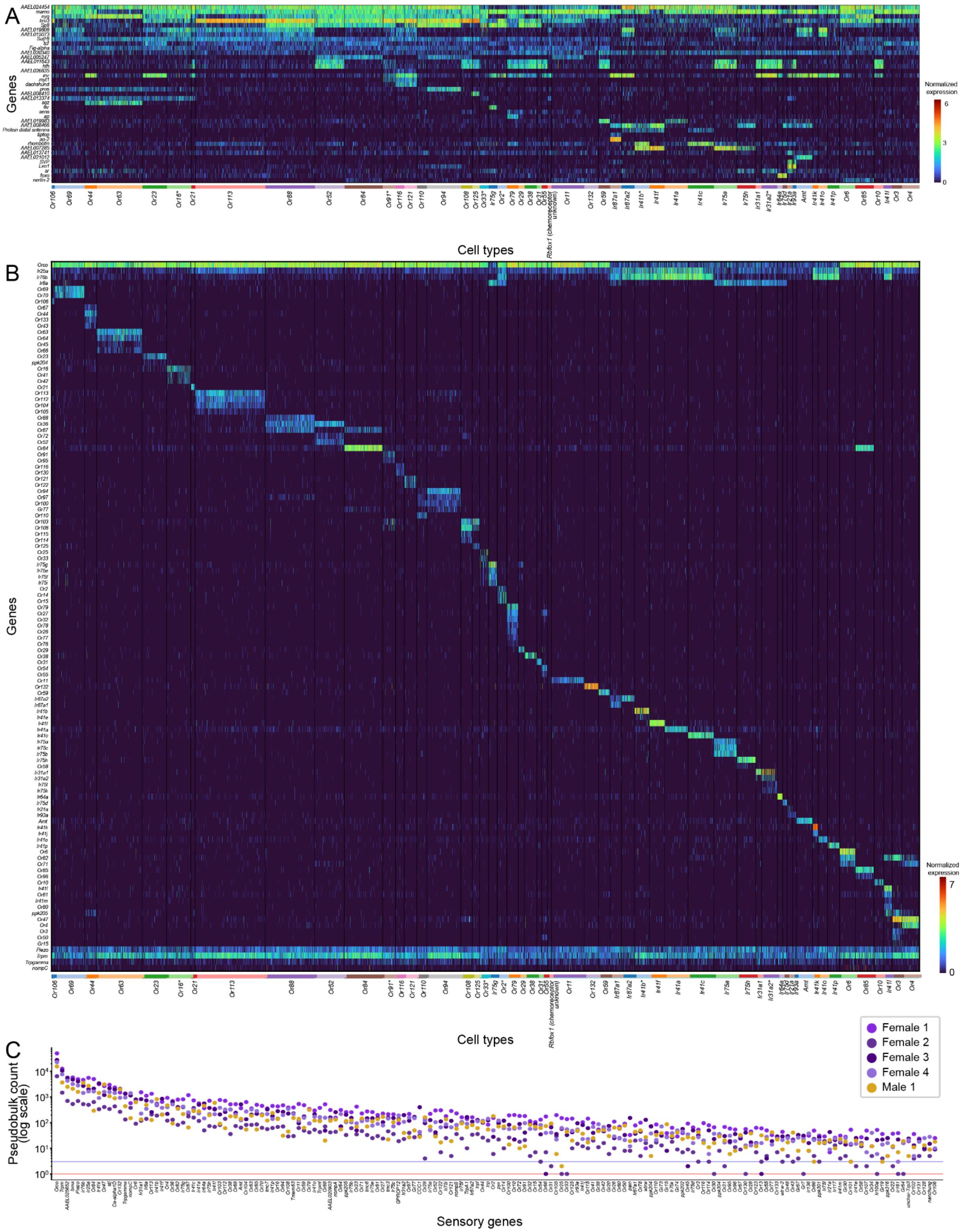

bioRxiv preprint doi: https://doi.org/10.1101/2025.02.25.639765; this version posted February 25, 2025. The copyright holder for this preprint (which was not certified by peer review) is the author/funder, who has granted bioRxiv a license to display the preprint in perpetuity. It is made available under aCC-BY 4.0 International license.

Figure S18. Antenna chemosensory neuron chemoreceptor and putative transcription factor factor expression profiles, related to Figure 5.
(A-B) Heatmap of genes expressed across all antenna olfactory sensory neurons (*nompC*-negative) for putative selected transcription factors (A) and selected sensory genes (B). Selected genes are indicated in rows and cells indicated in columns. Cells are grouped by annotation in Figure 5C and indicated below heatmap. Asterisks (*) on cell type annotation indicates that a group may represent a co-clustering of cells with multiple unique sensory gene expression patterns, as depicted in Figure 5C-D. Heatmap colors represent normalized expression. Normalized expression is ln([(raw count/total cell counts)x median total counts across cells]+1).
(C) Scatterplot of summed raw counts of sensory genes within all olfactory sensory neurons within each antenna sample. Red line indicates count of 1, blue line is a count of 5. For a complete look up table of gene symbols to gene IDs used in this manuscript, see Table S3. 158 out of 403 putative sensory genes (Table S4) with the highest counts shown for space (full plot available in Zenodo Supplemental Data).

bioRxiv preprint doi: https://doi.org/10.1101/2025.02.25.639765; this version posted February 25, 2025. The copyright holder for this preprint (which was not certified by peer review) is the author/funder, who has granted bioRxiv a license to display the preprint in perpetuity. It is made available under aCC-BY 4.0 International license.

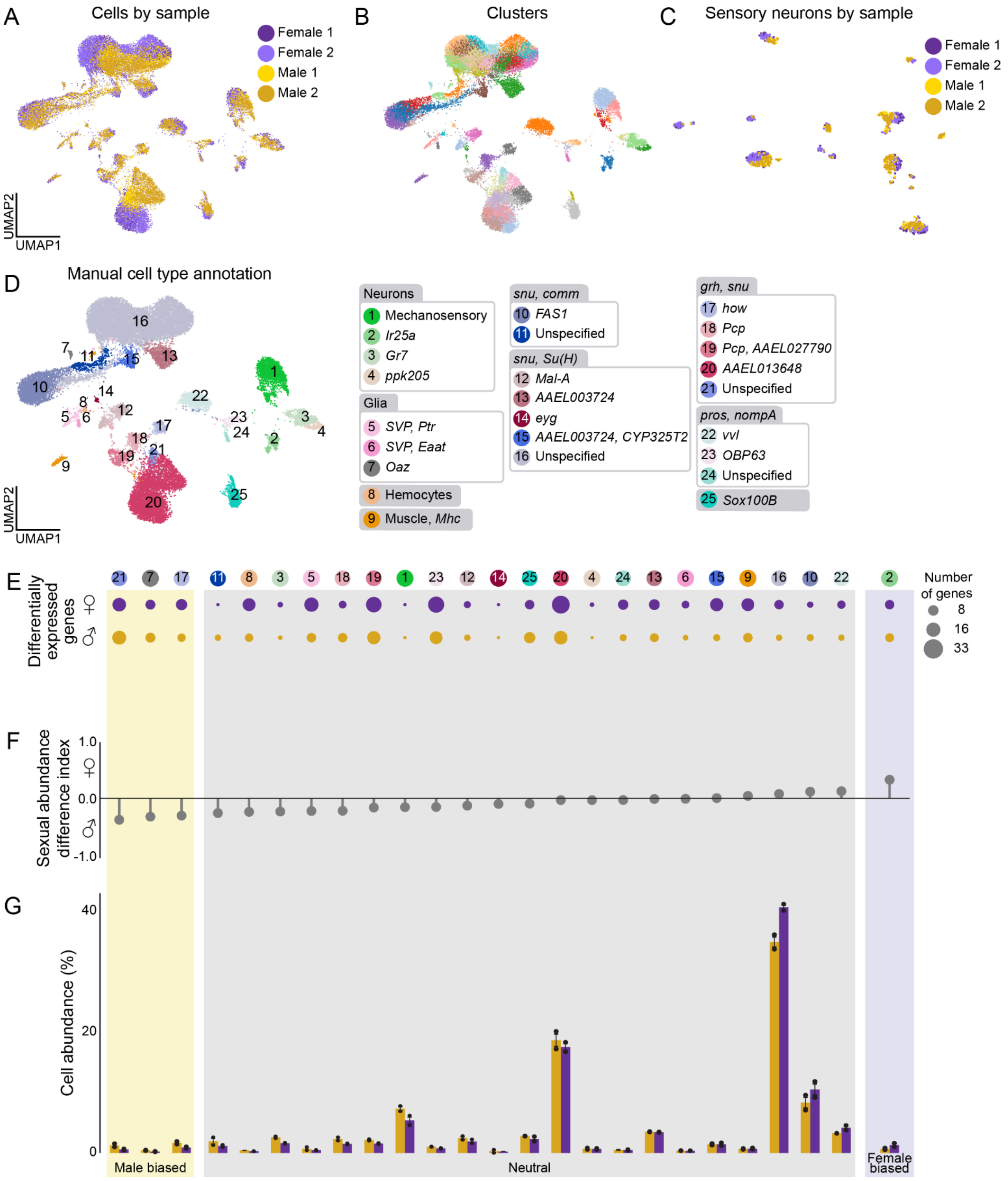

bioRxiv preprint doi: https://doi.org/10.1101/2025.02.25.639765; this version posted February 25, 2025. The copyright holder for this preprint (which was not certified by peer review) is the author/funder, who has granted bioRxiv a license to display the preprint in perpetuity. It is made available under aCC-BY 4.0 International license.

Figure S19. Tarsi samples and annotation, related to Figures 1, 5 and 6.
(A) UMAP of tarsi nuclei, colored by sample (female samples = 2, male samples = 2)
(B) UMAP of tarsi nuclei clustered using the Leiden algorithm (resolution = 5).
(C) UMAP of *nompC*-negative tarsi sensory neuron nuclei, colored by sample (female samples = 2, male samples = 2), related to Figure 6C.
(D) UMAP of nuclei from all tarsi samples, colored and numbered by manual annotation using selected marker genes as listed in legend at the right of the figure panel. See Table S3 and Table S4 for gene IDs and thresholds.
(E-G) Quantification of differences between male and female samples. Numbered and colored circles above (E) correspond to annotations in (D).
(E) Dot plot of number of differentially expressed genes between females and males in each cell type. Dots represent relative number of differentially expressed genes (DEG) upregulated in male or female with a |log fold change| > 1 and false discovery rate < 0.05, determined by MAST on normalized expression and converted to $\log_2$ fold change.
(F) Sexual abundance difference index from data in (G). Each cell type was categorized based on the following index: Female biased (purple) if 0.3 < abundance index; Neutral (grey) if -0.3 =< abundance index =< 0.3; Male biased (yellow) if abundance index < -0.3.
(G) Bar plot of relative cell abundance, represented as the percent of each cell type of all nuclei collected from each sex for tissue. Separated by female (purple) and male (yellow). Values for each replicate are shown as dots, and standard error indicated by black brackets.

bioRxiv preprint doi: https://doi.org/10.1101/2025.02.25.639765; this version posted February 25, 2025. The copyright holder for this preprint (which was not certified by peer review) is the author/funder, who has granted bioRxiv a license to display the preprint in perpetuity. It is made available under aCC-BY 4.0 International license.

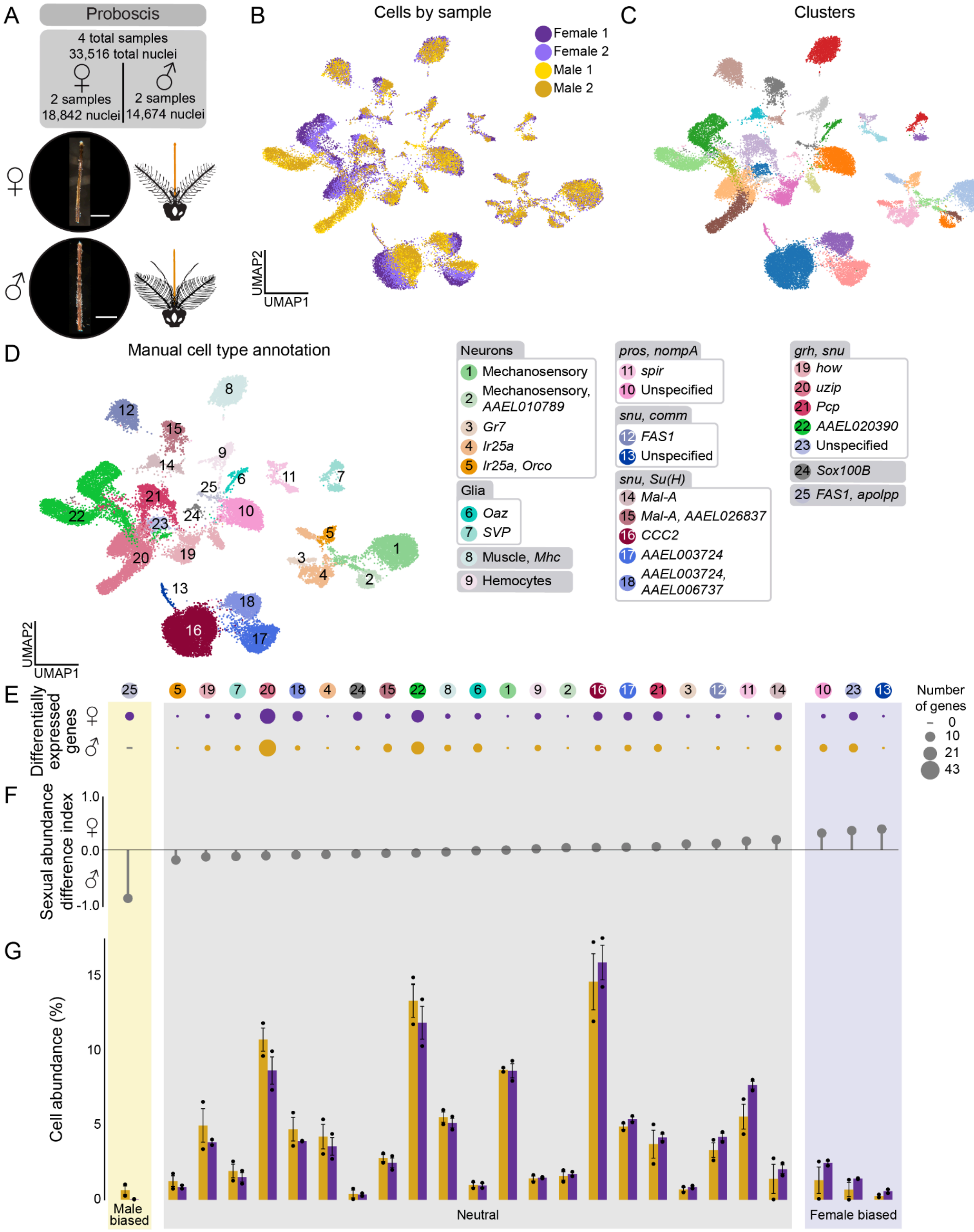

bioRxiv preprint doi: https://doi.org/10.1101/2025.02.25.639765; this version posted February 25, 2025. The copyright holder for this preprint (which was not certified by peer review) is the author/funder, who has granted bioRxiv a license to display the preprint in perpetuity. It is made available under aCC-BY 4.0 International license.

Figure S20. Proboscis samples and annotation, related to Figures 1, 5 and 6.
(A) Photo of dissected female (top) and male (bottom) proboscis with anatomical diagram of proboscis dissection (in orange), and collected sample information. Scale bar: 500 µm.
(B) UMAP of proboscis nuclei, colored by sample (female samples = 2, male samples = 2)
(C) UMAP of proboscis nuclei clustered using the Louvain algorithm (resolution = 1).
(D) UMAP of nuclei from all proboscis samples, colored and numbered by manual annotation using selected marker genes as listed in legend at the right of the figure panel. See Table S3 and Table S4 for gene IDs and thresholds.
(E-G) Quantification of differences between male and female samples. Numbered and colored circles above (E) correspond to annotations in (D).
(E) Dot plot of number of differentially expressed genes between females and males in each cell type. Dots represent relative number of differentially expressed genes (DEG) upregulated in male or female with a |log fold change| > 1 and false discovery rate < 0.05, determined by MAST on normalized expression and converted to $\log_2$ fold change.
(F) Sexual abundance difference index from data in (G). Each cell type was categorized based on the following index: Female biased (purple) if 0.3 < abundance index; Neutral (grey) if -0.3 =< abundance index =< 0.3; Male biased (yellow) if abundance index < -0.3.
(G) Bar plot of relative cell abundance, represented as the percent of each cell type of all nuclei collected from each sex for tissue. Separated by female (purple) and male (yellow). Values for each replicate shown as dots, and standard error indicated by black brackets.

bioRxiv preprint doi: https://doi.org/10.1101/2025.02.25.639765; this version posted February 25, 2025. The copyright holder for this preprint (which was not certified by peer review) is the author/funder, who has granted bioRxiv a license to display the preprint in perpetuity. It is made available under aCC-BY 4.0 International license.

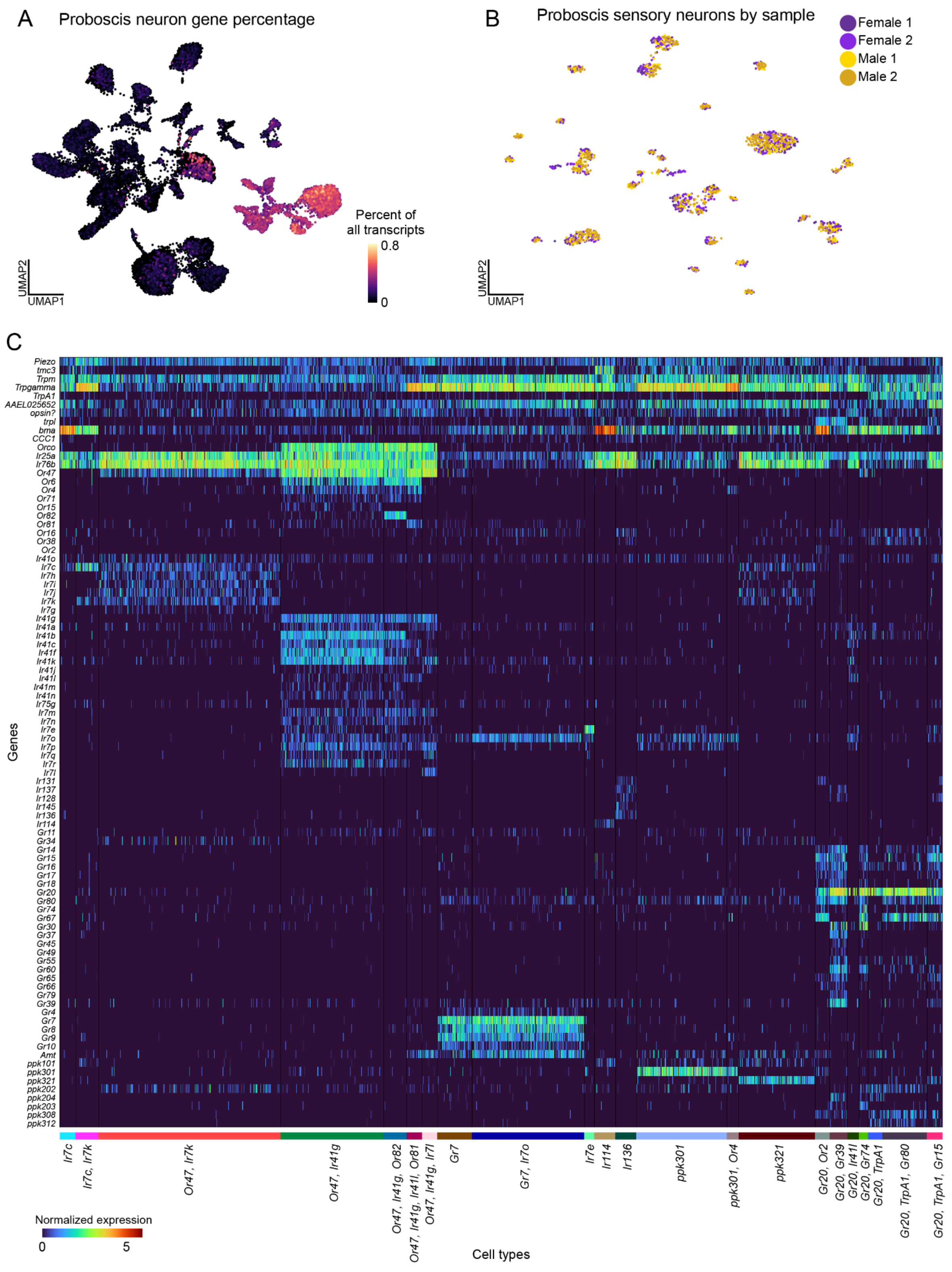

bioRxiv preprint doi: https://doi.org/10.1101/2025.02.25.639765; this version posted February 25, 2025. The copyright holder for this preprint (which was not certified by peer review) is the author/funder, who has granted bioRxiv a license to display the preprint in perpetuity. It is made available under aCC-BY 4.0 International license.

Figure S21. Proboscis sensory gene analysis, related to Figures 5 and 6.
(A) UMAP of all proboscis cells with expression of neuron marker genes (*Syt1 (AAEL000704), nSyb (AAEL024921), brp (AAEL018153), CadN (AAEL000597)* as percent of all transcripts in each cell. For *nompC* gene percentage, see Figure S22B.
(B) UMAP of reclustered sensory cells colored by sample (female = 2, male = 2).
(C) Heatmap of cells from all annotated clusters. Sensory genes are indicated in rows and cells indicated in columns. Cell types indicated below heatmap and respective sensory function indicated above. Heatmap colors represent normalized expression. Normalized expression is ln([(raw count/total cell counts)x median total counts across cells]+1).

bioRxiv preprint doi: https://doi.org/10.1101/2025.02.25.639765; this version posted February 25, 2025. The copyright holder for this preprint (which was not certified by peer review) is the author/funder, who has granted bioRxiv a license to display the preprint in perpetuity. It is made available under aCC-BY 4.0 International license.

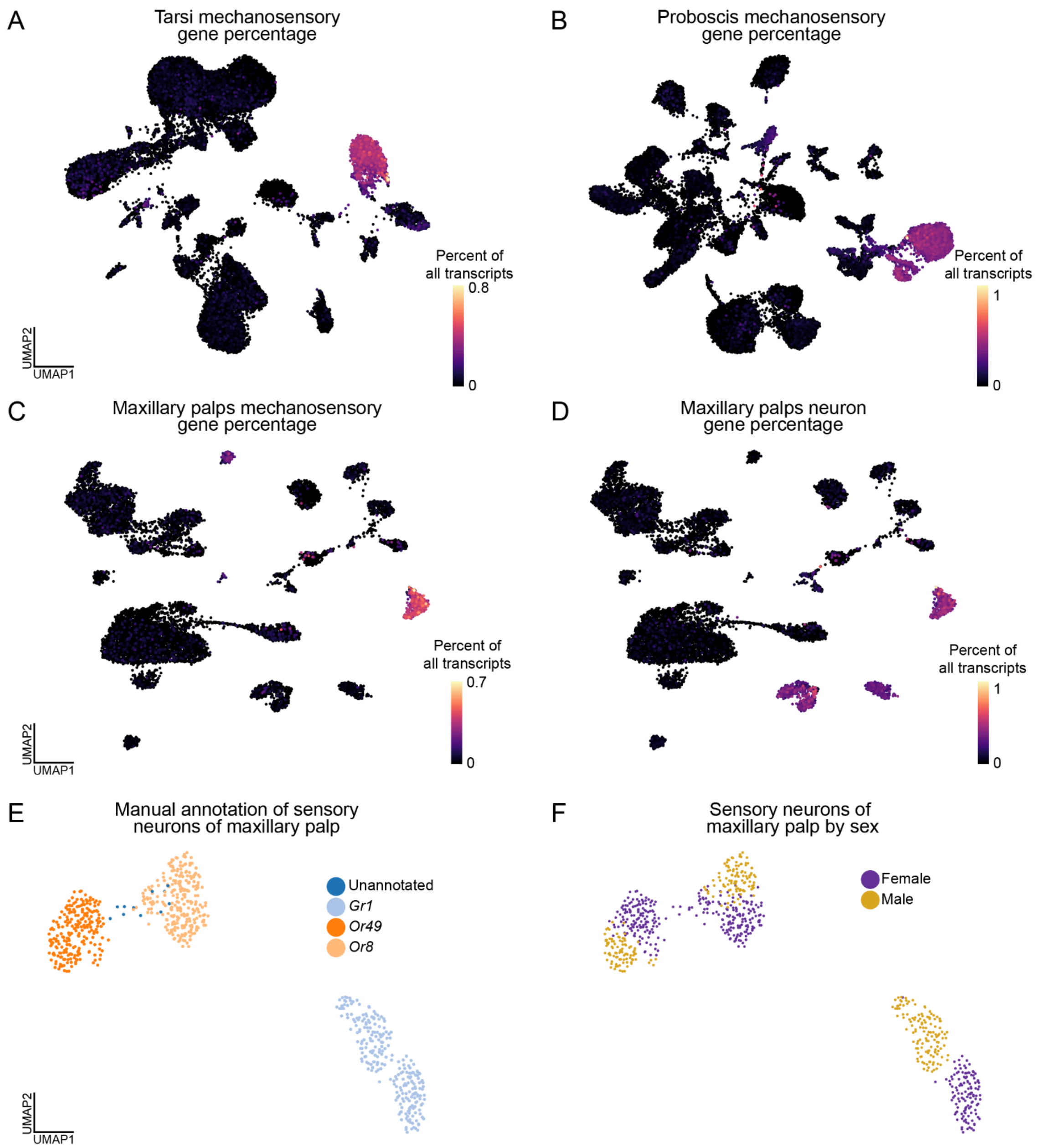

Figure S22. Sensory neuron filtering and analysis, related to Figures 5 and 6.
(A-C) UMAP of neurons from tarsi (A), proboscis (B), and maxillary palp (C) data with expression of *nompC* (*AAEL019818*) as percent of all transcripts in each cell.
(D) UMAP of all maxillary palp cells with expression of neuron marker genes (*Syt1 (AAEL000704), nSyb (AAEL024921), brp (AAEL018153), CadN (AAEL000597)*) as percent of all transcripts in each cell.
(E-F) UMAP of *nompC*-negative sensory neurons from maxillary palp, colored by manual annotation (E) and sample (F) (female = 1, male = 1). Female sample previously published[43].

bioRxiv preprint doi: https://doi.org/10.1101/2025.02.25.639765; this version posted February 25, 2025. The copyright holder for this preprint (which was not certified by peer review) is the author/funder, who has granted bioRxiv a license to display the preprint in perpetuity. It is made available under aCC-BY 4.0 International license.

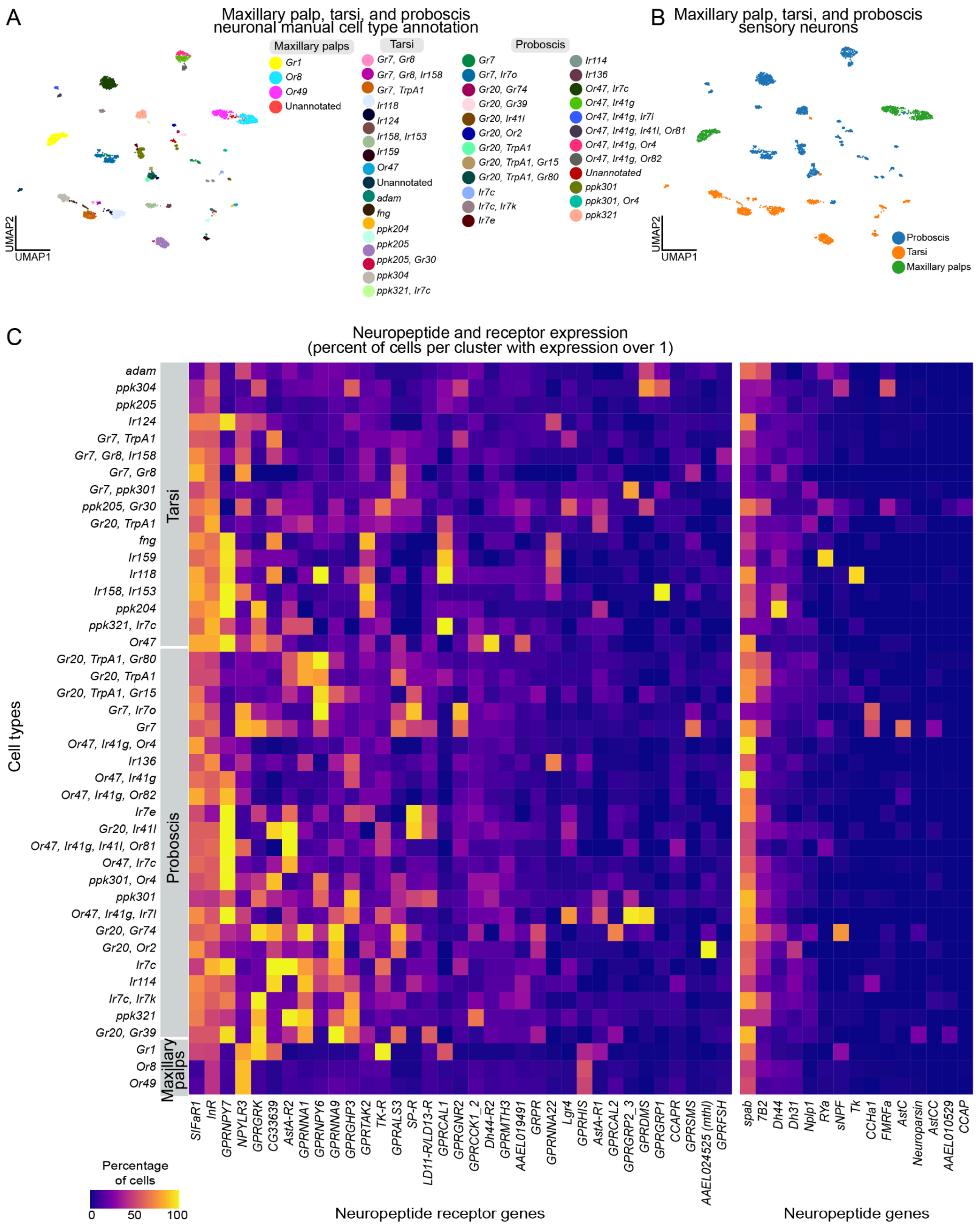

bioRxiv preprint doi: https://doi.org/10.1101/2025.02.25.639765; this version posted February 25, 2025. The copyright holder for this preprint (which was not certified by peer review) is the author/funder, who has granted bioRxiv a license to display the preprint in perpetuity. It is made available under aCC-BY 4.0 International license.

Figure S23. Neuropeptide receptor and synthesis gene analysis, related to Figures 5 and 6.
(A-B) UMAP of combined *nompC*-negative sensory neurons from maxillary palp, tarsi and proboscis samples, colored by manual cell-type annotation as listed in legend at the right of the figure panel (A) and original tissue (B).
(C) Heatmap of expression of neuropeptide receptor genes (left) and neuropeptide synthesis genes (right) within manually annotated *nompC*-negative sensory neurons in the maxillary palp, tarsi and proboscis. Color scale indicates percentage of cells expressing a gene above threshold (normalized expression value of 1). Sensory genes are indicated in columns and cell types indicated in rows. Genes were included if they were expressed above threshold in over 20% of cells in at least one cell type. Genes filtered from lists in Table S4. Normalized expression is ln([(raw count/total cell counts)x median total counts across cells]+1).

bioRxiv preprint doi: https://doi.org/10.1101/2025.02.25.639765; this version posted February 25, 2025. The copyright holder for this preprint (which was not certified by peer review) is the author/funder, who has granted bioRxiv a license to display the preprint in perpetuity. It is made available under aCC-BY 4.0 International license.

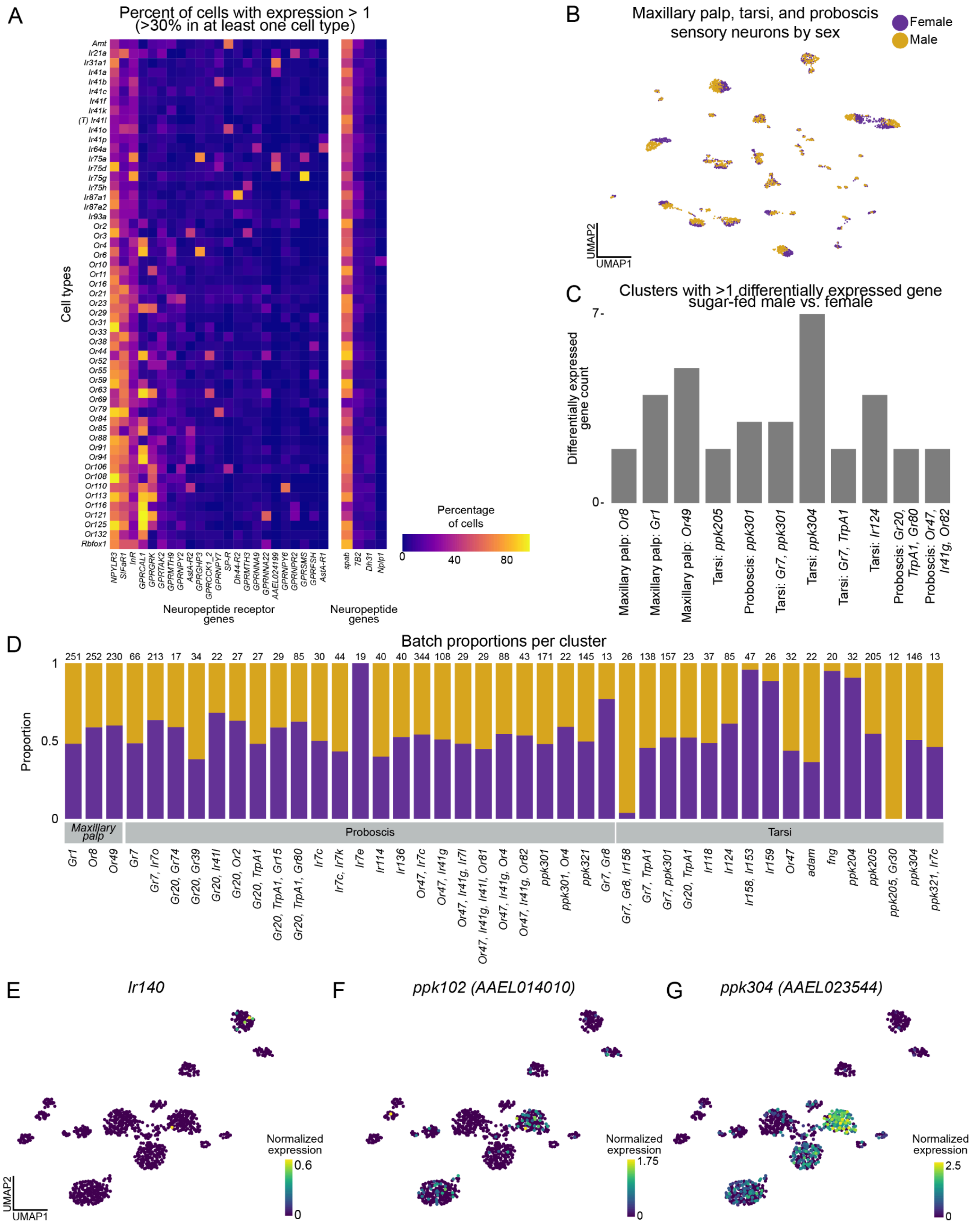

bioRxiv preprint doi: https://doi.org/10.1101/2025.02.25.639765; this version posted February 25, 2025. The copyright holder for this preprint (which was not certified by peer review) is the author/funder, who has granted bioRxiv a license to display the preprint in perpetuity. It is made available under aCC-BY 4.0 International license.

Figure S24. Neuropeptide receptor and synthesis gene analysis, related to Figures 5 and 6.

(A) Heatmap of expression of neuropeptide receptor genes (left) and neuropeptide synthesis genes (right) within manually annotated olfactory sensory neurons (*nompC*-negative) in the antenna. Color scale indicates percentage of cells expressing a gene above threshold (normalized expression value of 1). Sensory genes are indicated in columns and cell types indicated in rows. Genes were included if they were expressed above threshold in over 20% of cells in at least one cell type. Gnees filtered from lists in Table S2. Normalized expression is ln([(raw count/total cell counts)x median total counts across cells]+1).

(B) UMAP of combined *nompC*-negative sensory neurons from maxillary palp, tarsi and proboscis samples, colored by sex.

(C) Bar plot of number of differentially expressed genes between females and males in each olfactory sensory neuron cell type. Clusters shown contain at least 1 differentially expressed genes (DEG) with a |log fold change| > 1 and false discovery rate < 0.05 (determined by MAST on normalized expression).

(D) Proportion plot for annotated *nompC*-negative sensory neurons from maxillary palp, tarsi and proboscis. Bar plot of proportion cells from each sex that makes up each cell type. Numbers above each bar indicate the number of cells in that cluster. Cell types that are more than 70% from a single sex indicated in red.

(E-G) In tarsi *nompC*-negative sensory neurons, UMAP of normalized gene expression of *Ir140* (E), *ppk102 (AAEL014010)* (F), and *ppk304 (AAEL023544)* (G). Color bar trimmed 0.1% for visibility.

bioRxiv preprint doi: https://doi.org/10.1101/2025.02.25.639765; this version posted February 25, 2025. The copyright holder for this preprint (which was not certified by peer review) is the author/funder, who has granted bioRxiv a license to display the preprint in perpetuity. It is made available under aCC-BY 4.0 International license.

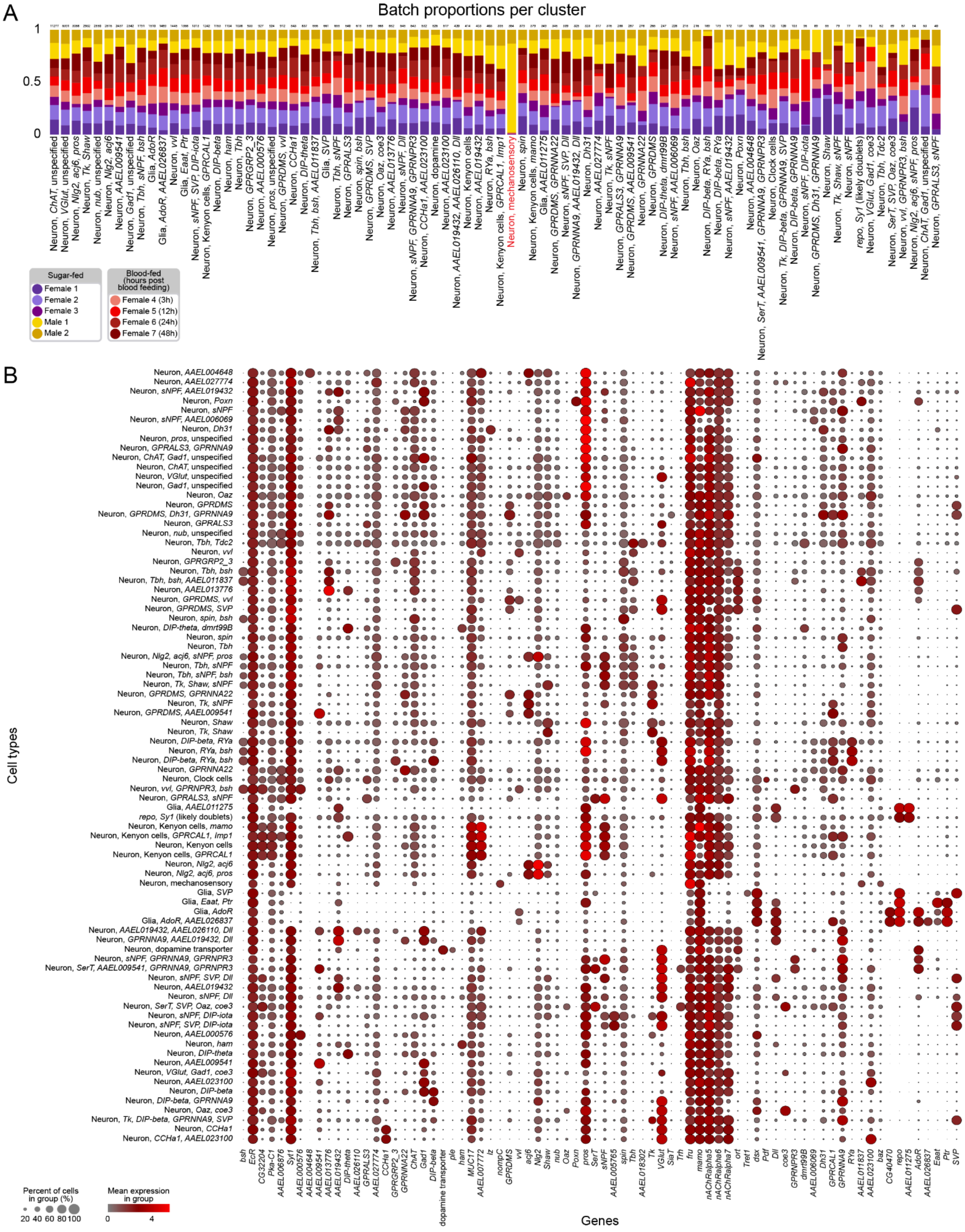

bioRxiv preprint doi: https://doi.org/10.1101/2025.02.25.639765; this version posted February 25, 2025. The copyright holder for this preprint (which was not certified by peer review) is the author/funder, who has granted bioRxiv a license to display the preprint in perpetuity. It is made available under aCC-BY 4.0 International license.

Figure S25. Brain Cell types, related to Figures 7 and 8.
(A) Proportion plot for annotated brain nuclei. Bar plot of proportion that each sample makes up each cell type. Numbers above each bar indicate the number of cells in that cluster. Cell types that are more than 70% from a single sample indicated in red.
(B) Dotplot of brain nuclei annotations and gene markers. Color scale indicates mean normalized expression of gene within cell type, size of dot indicates percent of cells expressing gene within the group. See Table S3 and Table S4 for gene IDs and thresholds.

bioRxiv preprint doi: https://doi.org/10.1101/2025.02.25.639765; this version posted February 25, 2025. The copyright holder for this preprint (which was not certified by peer review) is the author/funder, who has granted bioRxiv a license to display the preprint in perpetuity. It is made available under aCC-BY 4.0 International license.

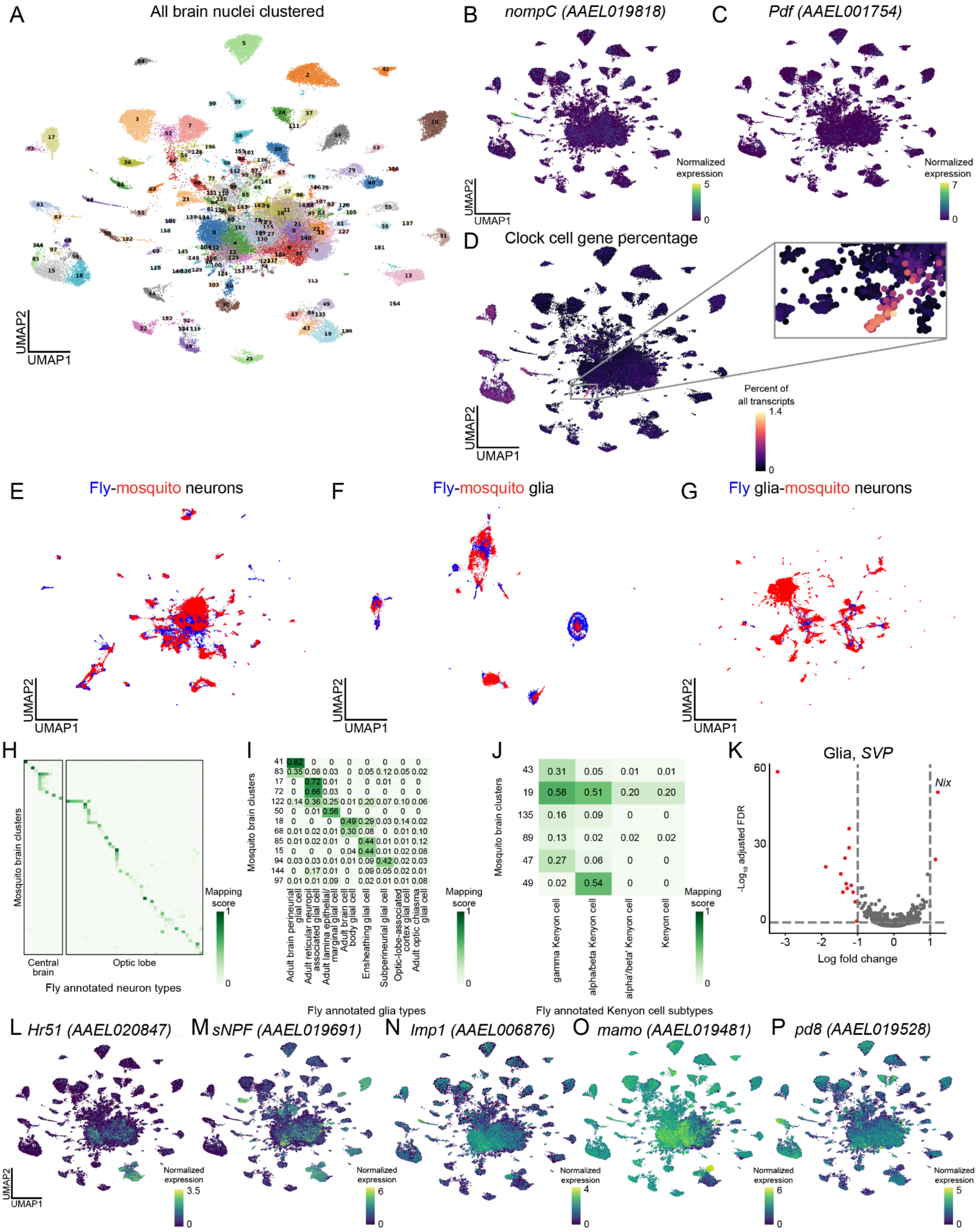

bioRxiv preprint doi: https://doi.org/10.1101/2025.02.25.639765; this version posted February 25, 2025. The copyright holder for this preprint (which was not certified by peer review) is the author/funder, who has granted bioRxiv a license to display the preprint in perpetuity. It is made available under aCC-BY 4.0 International license.

Figure S26. SAMap analysis and identification of clock cells and kenyon cells, related to Figure 7.
(A) UMAP of brain nuclei clustered using the Leiden algorithm (resolution = 5).
(B-C) Normalized gene expression UMAP of *nompC (AAEL019818)* (B) and *Pdf (AAEL001754)* (C) in all brain nuclei. Normalized expression is ln([(raw count/total cell counts)x median total counts across cells]+1).
(D) Normalized expression UMAP of gene set of 10 putative clock cell gene markers (Table S4). Expression of gene set shown as a fraction of total transcripts in each cell. Cluster with high expression highlighted in gray box, enlarged in inset.
(E-G) UMAP of manifold integration of snRNA-seq data from *Aedes aegypti* mosquito brain with published *Drosophila melanogaster* fly head[25]. Plots show integration of fly neurons with mosquito neurons (E), fly glia with mosquito glia (F), and as a control, fly glia with mosquito neuron (G). Alignment scores are 0.64, 0.64 and 0.47, respectively.
(H-J) Correlation matrices of mapping scores between *Drosophila melanogaster* head annotations and *Aedes aegypti* of clusters (Leiden, resolution = 5) for neuronal cell types **(H)**, glial cell types (I), and Kenyon cell subtypes (J). Calculated by SAMap. For numerical values, see Tables S7-S8.
(K) Volcano plot of differentially expressed genes in the "*SVP*" glia cell cluster by sex. All significant genes (indicated in red) a |log fold change| > 1 and false discovery rate < 0.05, determined by MAST on normalized expression. Male biased genes on right, as indicated by *Nix* (*AAEL022912*).
(L-P) Normalized gene expression UMAP of *Hr51 (AAEL020847)* (L), *sNPF (AAEL019691)* (M), *Imp1 (AAEL006876)* (N), *mamo (AAEL019481)* (O), *Pde8 (AAEL019528)* (P) in all brain nuclei.

bioRxiv preprint doi: https://doi.org/10.1101/2025.02.25.639765; this version posted February 25, 2025. The copyright holder for this preprint (which was not certified by peer review) is the author/funder, who has granted bioRxiv a license to display the preprint in perpetuity. It is made available under aCC-BY 4.0 International license.

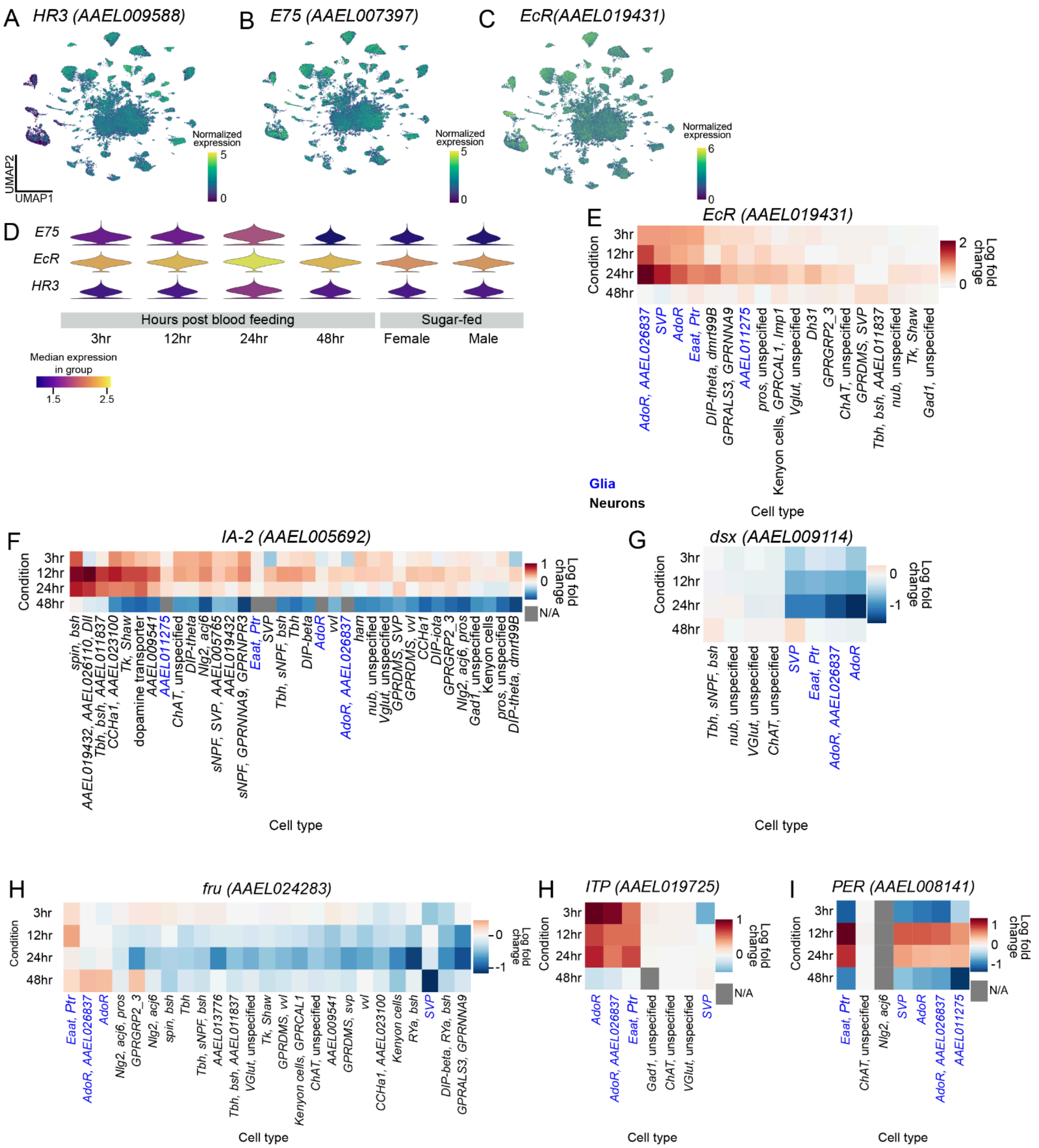

bioRxiv preprint doi: https://doi.org/10.1101/2025.02.25.639765; this version posted February 25, 2025. The copyright holder for this preprint (which was not certified by peer review) is the author/funder, who has granted bioRxiv a license to display the preprint in perpetuity. It is made available under aCC-BY 4.0 International license.

Figure S27. Blood feeding changes in brain, related to Figure 8.
(A-C) Normalized gene expression UMAP of *E75* (*AAEL007397*) (A), *EcR* (*AAEL019431*) (B) and *HR3* (*AAEL009588*) (C) in all brain nuclei. Normalized expression is ln([(raw count/total cell counts)x median total counts across cells]+1).
(D) Violin plot of gene expression of *E75*, *EcR* and *HR3* across all brain nuclei in each timepoint.
(E-J) Heatmaps of log fold change genes across blood feeding conditions compared to sugar-fed female brain by cell type. Genes shown: *EcR (AAEL019431)* (E), *IA-2 (AAEL005692)* (F), *dsx* (*AAEL009114*) (G), *fru* (*AAEL0124283*) (H), *ITP* (*AAEL019725*) (I), and *PER* (*AAEL008141*) (J). Log fold change is determined by MAST on normalized expression. Cell types are sorted by the total log fold change across all timepoints and colored as glia (blue) or neurons (black). Cell types included have over 10 cells in each timepoint, and at least one timepoint where change from sugar-fed condition had a false discovery rate < 0.05. Grey boxes indicate log fold change data is not available, due to zero expression within cell type in either sugar fed or blood fed condition. Normalized expression is ln([(raw count/total cell counts)x median total counts across cells]+1).

Supplemental table legends

Table S1. Dissection and sample processing information by sample.
Sample information including number of mosquitoes dissected, FASTQ prefixes, number of cells, number and percent of cells filtered, whether FACS was used, sample processing parameters, Cell Ranger statistics, CellBender parameters, quality control filtering thresholds and percent of cells removed, final cell count, and additional notes.

Table S2. Processed data objects on UCSC Cell Browser and Zenodo Supplemental Data.
Information on processed data files for each tissue and filtered sensory neuron population. Includes number of samples per tissue, number of cells, annotation and visualization parameters, clustering resolution and algorithm used for cell type annotations were assigned, and filtering parameters for sensory neuron data objects (if applicable).

Table S3. Gene identifiers, symbols, descriptions and *Drosophila melanogaster* orthology.
Look up table of all genes used in this manuscript. Includes gene symbol used within this manuscript, and corresponding Vector Base gene ID (AAEL)[57], NCBI gene symbol (LOC)[208], descriptions, references, and *Drosophila melanogaster* homology information from BioMart (Ensembl Genomes release 56[55]).

Table S4. Annotation thresholds and gene lists.
Gene lists for salivary gland secreted proteins, antimicrobial peptides, Kenyon Cell markers, clock cell markers, sensory proteins, neuropeptides and neuropeptide receptors. Also includes genes used to annotate all cell types of each tissue, including gene thresholds.

Table S5. Thresholded differentially expressed genes for antenna olfactory

bioRxiv preprint doi: https://doi.org/10.1101/2025.02.25.639765; this version posted February 25, 2025. The copyright holder for this preprint (which was not certified by peer review) is the author/funder, who has granted bioRxiv a license to display the preprint in perpetuity. It is made available under aCC-BY 4.0 International license.

sensory neurons, between male and female cells across each cell type.
Differentially expressed genes between females and males for each antenna olfactory sensory neuron cell type. Filtered for genes with |log fold change| > 1 and false discovery rate < 0.05 (determined by MAST on normalized expression).

Table S6. Thresholded differentially expressed genes for *nompC*-negative sensory neurons in maxillary palp, proboscis, and tarsi, between male and female cells across each cell type.
Differentially expressed genes between females and males for each *nompC*-negative sensory neuron cell type in maxillary palp, proboscis, and tarsi. Filtered for genes with |log fold change| > 1 and false discovery rate < 0.05 (determined by MAST on normalized expression).

Table S7. Manifold integration mapping scores of neurons from *Aedes aegypti* brain and *Drosophila melanogaster* head.
Correlation matrices of mapping scores between neurons using annotated *Drosophila melanogaster* head cell types and *Aedes aegypti* brain clusters (Leiden, resolution = 5). Includes corresponding annotations from this paper.

Table S8. Manifold integration mapping scores of glia from *Aedes aegypti* brain and *Drosophila melanogaster* head.
Correlation matrices of mapping scores between glia using annotated *Drosophila melanogaster* head cell types and *Aedes aegypti* brain clusters (Leiden, resolution = 5). Includes corresponding annotations from this paper.

Table S9. Thresholded differentially expressed genes for annotated brain cell types, between male and female cells across each cell type.
Differentially expressed genes between females and males for each annotated brain cell type. Filtered for genes with |log fold change| > 1 and false discovery rate < 0.05 (determined by MAST on normalized expression).

Table S10. Thresholded differentially expressed genes for annotated brain cell types, between post-bloodfeeding timepoints compared to sugarfed female cells across each cell type.
Differentially expressed genes between each post-bloodfeeding timepoint compared to sugar-fed female cells for each annotated brain cell type. Filtered for genes with |log fold change| > 1 and false discovery rate < 0.05 (determined by MAST on normalized expression).

Table S11. RNA in situ hybridization probe information.
Table includes targeted gene names, HCR Amplifier, probe sequences and probe lot numbers for ordering.
